# Exploring Regional Variation in Roost Selection by Bats: Evidence from a Meta-Analysis

**DOI:** 10.1371/journal.pone.0139126

**Published:** 2015-09-29

**Authors:** François Fabianek, Marie Anouk Simard, André Desrochers

**Affiliations:** 1 Centre d’Étude de la Forêt (CEF), and Faculté de Foresterie, de Géographie et de Géomatique, Université Laval, Québec, Quebec, Canada; 2 Ministère des Forêts, de la Faune et des Parcs, Québec, Quebec, Canada; 3 Centre de la Sciences de la Biodiversité du Québec, and Faculté de Foresterie, de Géographie et de Géomatique, Université Laval, Québec, Quebec, Canada; University of Western Ontario, CANADA

## Abstract

**Background and Aims:**

Tree diameter, tree height and canopy closure have been described by previous meta-analyses as being important characteristics in roost selection by cavity-roosting bats. However, size and direction of effects for these characteristics varied greatly among studies, also referred to as heterogeneity. Potential sources of heterogeneity have not been investigated in previous meta-analyses, which are explored by correlating additional covariates (moderator variables). We tested whether effect sizes from 34 studies were consistent enough to reject the null hypothesis that trees selected by bats did not significantly differ in their characteristics from randomly selected trees. We also examined whether heterogeneity in tree diameter effect sizes was correlated to moderator variables such as sex, bat species, habitat type, elevation and mean summer temperature.

**Methods:**

We used Hedges’ g standardized mean difference as the effect size for the most common characteristics that were encountered in the literature. We estimated heterogeneity indices, potential publication bias, and spatial autocorrelation of our meta-data. We relied upon meta-regression and multi-model inference approaches to evaluate the effects of moderator variables on heterogeneity in tree diameter effect sizes.

**Results:**

Tree diameter, tree height, snag density, elevation, and canopy closure were significant characteristics of roost selection by cavity-roosting bats. Size and direction of effects varied greatly among studies with respect to distance to water, tree density, slope, and bark remaining on trunks. Inclusion of mean summer temperature and sex in meta-regressions further explained heterogeneity in tree diameter effect sizes.

**Conclusions:**

Regional differences in roost selection for tree diameter were related to mean summer temperature. Large diameter trees play a central role in roost selection by bats, especially in colder regions, where they are likely to provide a warm and stable microclimate for reproductive females. Records of summer temperature fluctuations inside and outside tree cavities that are used by bats should be included in future research.

## Introduction

### Roosts selected by bats

Descriptions of roosts that are used by insectivorous bats in North American forests were mostly anecdotal prior to the mid-1990s. Technical developments in telemetry have been instrumental for our current understanding of habitat-species interactions with small mammals, such as bats [1].We now know that cavity- and bark-roosting bats rely upon living and standing dead trees (*i*.*e*., snags) in intermediate stages of decay [2, 3] for roosting [4, 5]. They have been reported roosting under exfoliating bark, inside trunk crevices, and within the cavities of both living and dead trees during the summer [2, 5–7]. The occurrence of several snags in a given stand likely indicates available roosts to bats [8, 9]. Bats are faithful to their roosting sites [10–12] and switch regularly from primary roosts (which are used more frequently) to alternate roosts [13, 14]. They therefore rely upon networks of clustered roost trees [15] that share similar characteristics, such as a large diameter and an intermediate stage of decay [2, 16], perhaps to minimize predation risk or to reduce commuting costs. Furthermore, snags are an ephemeral resource [17–19], which may explain—in addition to the aforementioned reasons—why bats favour a high density of snags near roosts [2, 6, 16, 20, 21].

Like roost availability, distance to resources (*e*.*g*., water and insect availability) appears to drive roost selection by bats. Roosts are commonly found in close proximity (*i*.*e*., < 10 km) to ponds and riparian habitats [1, 22–24], which provide good foraging conditions for bats [25–27]. Habitat complexity (*i*.*e*., level of vegetation clutter) and acoustic interferences are reduced over calm water bodies (*i*.*e*., ponds), increasing both prey detectability and capture success for bats [28, 29]. Moreover, the abundance and diversity of prey is generally greater over and near ponds [30–32]. Bats, especially lactating females [33], must rehydrate at dusk after roosting [33–35].

Sexual differences in the choice of roosts are often reported [36–39], with reproductive females (*i*.*e*., pregnant and lactating females) selecting for trees with larger diameters [36, 40] compared to males and non-reproductive females. Large diameter trees offer greater thermal inertia compared to trees with smaller diameters [40–42]. The thermoregulatory advantage of warm and stable temperatures [43–45] is commonly accepted as a major driver of roost selection by bats [46], especially in the case of reproductive females [5, 46–48].

Like males and non-reproductive females, reproductive females may also use torpor (*i*.*e*., state of reduced body temperature and metabolic rate) to reduce energy expenditures [49], but this comes at the cost of reduced milk production [50], and delayed fetal development and juvenile growth [5, 47]. To counteract these costs, lactating females may enter torpor for shorter bouts [48, 51] or adopt other behavioural strategies, such as social thermoregulation [52]. Sticking together to stay warm requires large tree cavities [53, 54], which underscores a central role that tree diameter plays in roost selection.

Like many birds and other small mammals [55], bats probably use passive rewarming to reduce energy expenditure during arousal, which requires an external heat source in the afternoon [43]. Several studies [16, 20, 56–58] proposed that canopy emergents and tall trees that are located within canopy openings or within stands of low tree density are more accessible to bats and also benefit from greater heat transfer by solar radiation [59]. Slope, slope aspect and elevation have also been associated with longer periods of external heating provided by solar radiation [7, 60, 61].

As has been suggested by Lacki, Cox [7], bats might favour trees that are located at lower elevations to benefit from warmer microclimates relative to those located at higher elevations. However, without temperature measurements on the field, it is difficult to establish a causal link between microclimate and roost selection by bats. For example, preference in elevation could also be related to variation in tree species composition [60, 62, 63]. Lacki, Baker [8] suggested that stands at lower elevations provide better roosting characteristics to bats (*i*.*e*., taller canopies, higher snag densities) than higher elevation stands.

### Previous narrative and quantitative reviews

The increasing number of published radio-telemetry studies has led to three systematic reviews [5, 47, 64] and three meta-analyses [1, 7, 65] that summarize habitat use by bats in both unmanaged and managed forests. In a previous systematic review, Miller, Arnett [64] suggested that most studies had small sample sizes and suffered from pseudo-replication, but the authors did not account for these caveats quantitatively. Unlike a systematic synthesis or a narrative review, a meta-analysis provides a statistical synthesis of literature by pooling effect sizes from several studies. Effect size reflects the strength of the difference between experimental and control group means [66]. Standardized effect sizes are commonly used in meta-analyses to compare results among studies independent of the scale of measurement [66].

By performing a meta-analysis, Lacki and Baker [65], and Kalcounis-Rueppell, Psyllakis [1] confirmed that tree diameter, tree height and canopy closure were important characteristics explaining roost selection by bats, despite notable differences in size and direction of effects (*i*.*e*., negative or positive effects) among studies. In a more recent meta-analysis of two bat species, Lacki, Cox [7] found that roosting requirements of Indiana bat (*Myotis sodalis*) and northern long-eared bat (*M*. *septentrionalis*) overlapped, except for tree diameter and variation in the type of roosts that were used. The authors concluded that the northern long-eared bat showed greater plasticity than the Indiana bat in the choice of roosting sites.

None of these meta-analyses (*i*.*e*., [1, 7, 65]) have tried to explain differences in effect sizes and in the direction of effects among studies, which is referred to as heterogeneity [66]. Heterogeneity is likely to be encountered in meta-analyses, since individual studies are conducted under various field conditions, use different methodologies and attempt to answer different questions [66, 67]. Meta-regression approaches are increasingly employed in meta-analyses to explore whether the heterogeneity may be correlated with additional covariates, which are referred to as moderator variables [67, 68]. Moderator variables may be included to test whether heterogeneity is associated with differences in study methods [66], or in the present case, with differences in roost selection that could be related to sex [36], bat species [7] or large-scale environmental factors [48].

### Aims and hypotheses

The growing awareness of global environmental issues has encouraged researchers to focus upon large-scale patterns in ecology, which are often extrapolated from small-scale studies with a limited sample size [69]. Detecting regional variation in roost selection using a meta-analysis may reveal large-scale patterns that cannot be explored locally. For example, based on observations from Britzke, Harvey [70] and Britzke, Hicks [71], Lacki, Cox [7] suggested that Indiana bats avoided roosting in upland habitats in regions near the northern end of the species distribution, with cooler climate and shorter growing season, with the converse occurring in southern populations (*sensu* Lacki, Cox [7]). In the same vein, Boland, Hayes [72] suggested that in the northern range of Keen’s myotis (*M*. *keenii*), reproductive females should select for trees with larger diameters, which likely provide warmer temperatures than smaller trees, due to relatively cold and short summers in Alaska compared to southern regions. Such large-scale hypotheses typically may be tested using meta-analysis coupled with a meta-regression approach [67].

A decade of research has passed since the last meta-analysis on North American bats was conducted [1] and the number of studies on roost selection by bats has doubled (S1 Table). There are now enough studies to investigate for regional differences in roost selection using meta-regression approaches and test large-scale hypotheses based on previous knowledge of bat roosting ecology during the summer.

Our first aim was to test whether the results for the most common characteristics in the literature were consistent enough among studies to reject the null hypothesis that trees selected by bats are not significantly different in their characteristics from randomly selected trees. We predicted that the effect sizes would be significantly different from zero and that the direction of effects would be consistent enough among studies (*i*.*e*., homogeneous) to reject the null hypothesis (*i*.*e*., no significant difference in characteristics from random trees) for each characteristic that we intended to test. After having identified the most consistent characteristics of roost selection by bats (*i*.*e*., with the strongest effect size), our second aim was to explain heterogeneity in tree diameter effect sizes by incorporating moderator variables such as habitat type, bat species, mean summer temperature, and elevation into a set of alternative meta-regression models. According to the microclimate hypothesis (*sensu* Boyles [46]), we predicted that reproductive females should select larger tree diameters (relative to random trees) in northern regions and at higher altitudes, because of lower mean summer temperatures, compared to southern regions and lower altitudes. We predicted that reproductive females and larger species of bats would require trees with larger diameters, compared to non-reproductive females and males [36, 40] or smaller species of bats [6]. We also predicted that larger tree diameters would be found in unmanaged (*i*.*e*., national parks) and riparian areas, compared to managed areas (*i*.*e*., where logging activity still occurs).

## Material and Methods

### Selection of studies

We searched for published bat-roost selection studies that were available online in Google Scholar and the Web of Science. Those included journal articles, government reports, Ph.D. and M.Sc. theses, book chapters, and symposia. We included most of the studies that were presented in Miller, Arnett [64], Barclay and Kurta [5], Kalcounis-Rueppell, Psyllakis [1], Lacki and Baker [65], and Lacki, Cox [7]. We retained only studies that reported comparisons between random and selected trees (*i*.*e*., case/control design). Because of distinct roosting ecologies [4, 14], we did not include studies on foliage-roosting bats, but retained those that dealt with bark- and cavity-roosting bats.

### Dataset extraction and preparation

Studies that compared different treatments or sites, or differences in roost selection among bat species, and between sexes, had more than one dataset. We regarded each dataset as a sample unit for our meta-analysis (expressed as *n* unless otherwise stated). We examined 20 candidate characteristics for explaining roosting preferences of cavity-roosting bats, but retained only nine for which we found a minimum of 10 studies (*i*.*e*., 19 datasets): tree diameter (cm; S1 Table), tree height (m; S2 Table), snag density (stems/0.1 ha; S3 Table), elevation (m; S4 Table), canopy closure (%; S5 Table), distance to water (m; S6 Table), tree density (stems/0.1 ha; S7 Table), slope (%; S8 Table), and bark remaining on trunks (%; S9 Table). We extracted means, standard errors, standard deviations, and sample sizes for each dataset. We converted standard errors to standard deviations by multiplying the standard error of the mean by the square-root of the sample size, *i*.*e*., the number of trees. We converted all measurements of size, density and distance to the same units. For each of the nine characteristics, we calculated Hedges’ g Standardized Mean Difference (SMD) as an estimate of the effect size between trees that had been selected by bats (*i*.*e*., experimental group) and random trees (*i*.*e*., control group), as suggested by Borenstein, Hedges [68].

We excluded studies with an effect size greater than 4 times the mean group standard deviation to meet criteria of effect size normality and variance homogeneity [68]. We computed prediction intervals, fixed-effects and random-effects models (*meta* package, R Development Core Team 2015 [73]) for comparison purposes, but used only random-effects models in our meta-analysis (S1–S9 Tables). Random-effects models assume that heterogeneity not only depends upon sampling variance but also random population effect sizes [68], which is the case in our meta-analysis involving numerous bat species, together with potential variation between sexes and among habitat types.

### Publication bias and heterogeneity

Testing for publication bias supposes that there is a tendency for publishing studies with significant findings. If such bias is present, studies should be unbalanced towards positive results with only a few published studies supporting the null hypothesis. Publication bias is considered null when studies are well balanced (*e*.*g*., when roughly the same number of studies have reported significant findings versus those supporting the null hypothesis). We used funnel plots (*i*.*e*., effect size plotted against its standard error) to assess potential publication bias [74] for each of the nine characteristics. We tested for funnel plot asymmetry using the conventional weighted linear regression method [75], which is provided in the package *meta* [76].

We used l’Abbé plots to display meta-data visually and to investigate potential patterns of heterogeneity. In l’Abbé plots, the experimental group is plotted against the control group and the resulting regression line and its associated 95% CI is compared visually with the equality line (1:1), for which the mean difference is null [77]. We used the maximum likelihood approach (package *meta*, R Development Core Team 2015) to estimate heterogeneity (*τ* ^2^) in the population effect sizes. We further quantified heterogeneity using Higgins' I^2^ index [78] (expressed as a percentage) and used the classification scheme that was given by the authors to interpret the severity of heterogeneity (see Higgins, Thompson [78] for further details).

### Moderator variables and meta-regressions

We geo-located study sampling sites by using GPS coordinates or the locations that were mentioned in the reviewed manuscripts. We registered these locations in ArcGIS (version 10.1, Environmental Systems Research Institute, Redlands, CA, USA), around which we drew 1 km-radius buffer zones to compensate for imprecision. We further integrated into ArcGIS raster maps of elevation (digital elevation model) and monthly mean temperature (from June to August) that were provided by WorldClim 1.4 [79]. We averaged the pixel values that overlapped the 1 km-radius buffer zones to generate summer mean temperature and elevation values. Monthly mean temperature and elevation raster maps that were provided by WorldClim 1.4 were generated through interpolation on a 30 arc-second resolution grid (*i*.*e*., 1 km^2^ spatial resolution) [79]. Monthly mean temperatures are based on daily minimum and maximum temperature fluctuations from 1950 to 2000 [80].

We extracted additional moderator variables from the reviewed manuscripts, such as sex (male, female, and combined), habitat type (managed areas, riparian areas and protected areas such as national parks), and bat species. Given the limited number of datasets (*n* = 63), we grouped bat species with fewer than 5 datasets by genus, resulting in only six classes of bat species. To interpret our meta-regression results correctly, we verified *a priori* that random-tree diameter was not correlated with latitude (*r* ^2^ = 0.00; *P* < 0.9). We verified that mean summer temperature was negatively correlated with elevation (*r* ^2^ = 0.21; *P* < 0.001) and latitude (*r* ^2^ = 0.77; *P* < 0.001). We decided to exclude latitude from our set of moderator variables since it was strongly correlated (*i*.*e*., *r* ≥ 0.7; [81]) with mean summer temperature.

**Fig 1:**
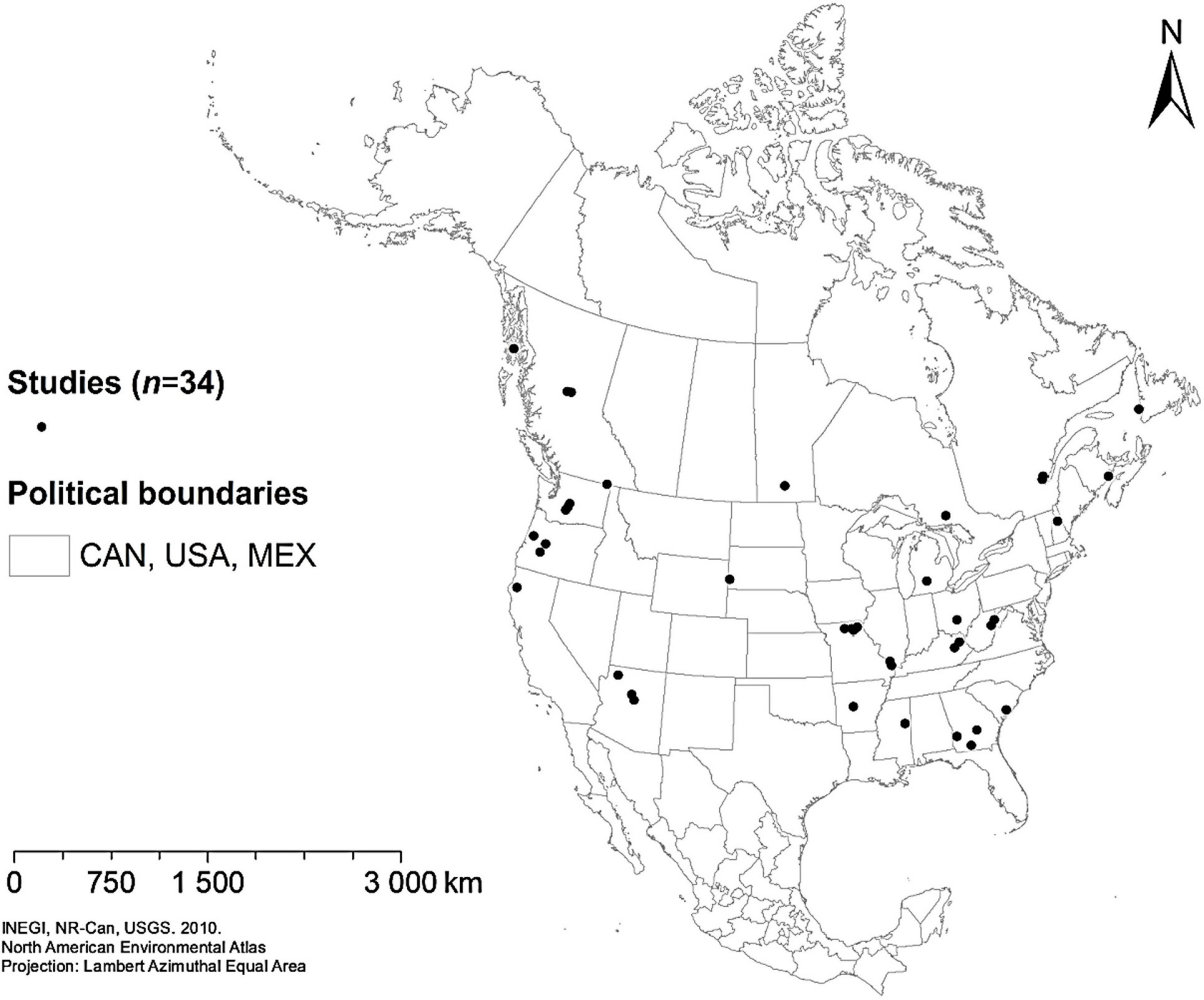
Locations of the 34 studies (66 datasets) that were included in meta-analysis of roost selection by North American bats. Map source: North American Environmental Atlas (INEGI, NR-Can, USGS, 2010).

Due to the apparent spatial proximity of several studies (Fig 1), we verified that our SMD estimates and our best meta-regression model residuals were not dependent upon the effect of spatial scale (*i*.*e*., they were not autocorrelated). We predicted that studies that are close to each other would not share similar SMD (and model residual) values and the converse for distant studies. In other words, we tested the null hypothesis of spatial randomness, for which SMD (and model residual) values would not depend upon values at neighbouring locations [82]. We choose *K* = 4 nearest studies as distance-based neighbours among studies. Once our neighbourhood of studies was created, we assigned spatial weights for each pair of neighbours, which was the inverse Euclidean distance among studies [82]. We performed a global Moran’s I test of spatial autocorrelation under randomization on the resulting Inverse Distance Weight (IDW) matrices [82]. We also used Moran's I test for residual spatial autocorrelation, which was provided in the package *spdep* [83].

We compared 17 candidate meta-regression models (package *metafor*, R Development Core Team 2015) to examine whether the heterogeneity in tree diameter effect sizes was explained by the aforementioned moderator variables. We constructed five subsets of candidate meta-regression models. The first set combined habitat type (*i*.*e*., management level), microclimate (*i*.*e*., mean summer temperature and elevation) and bat-related (*i*.*e*., bat species and sex) moderator variables. The second set combined both microclimate, and bat-related moderator variables. The third, fourth and fifth sets included only microclimate, bat-related or habitat type as moderator variables, respectively. We ranked the candidate set of models using the second-order Akaike’s Information Criterion for small samples (AICc). We calculated ∆AICc values (∆_*i*_) and Akaike weights (ω_*i*_) to determine the importance of the candidate set of models relative to the best explanatory model (∆_*i*_ = 0). Models were considered equivalent when they had a ∆AICc ≤ 2 [84]. We also included the pseudo-*R* ^2^ statistic provided by the package *metafor* [85], which estimates the amount of heterogeneity (%) accounted for by each candidate meta-regression model.

## Results

### Selected studies

**Fig 2:**
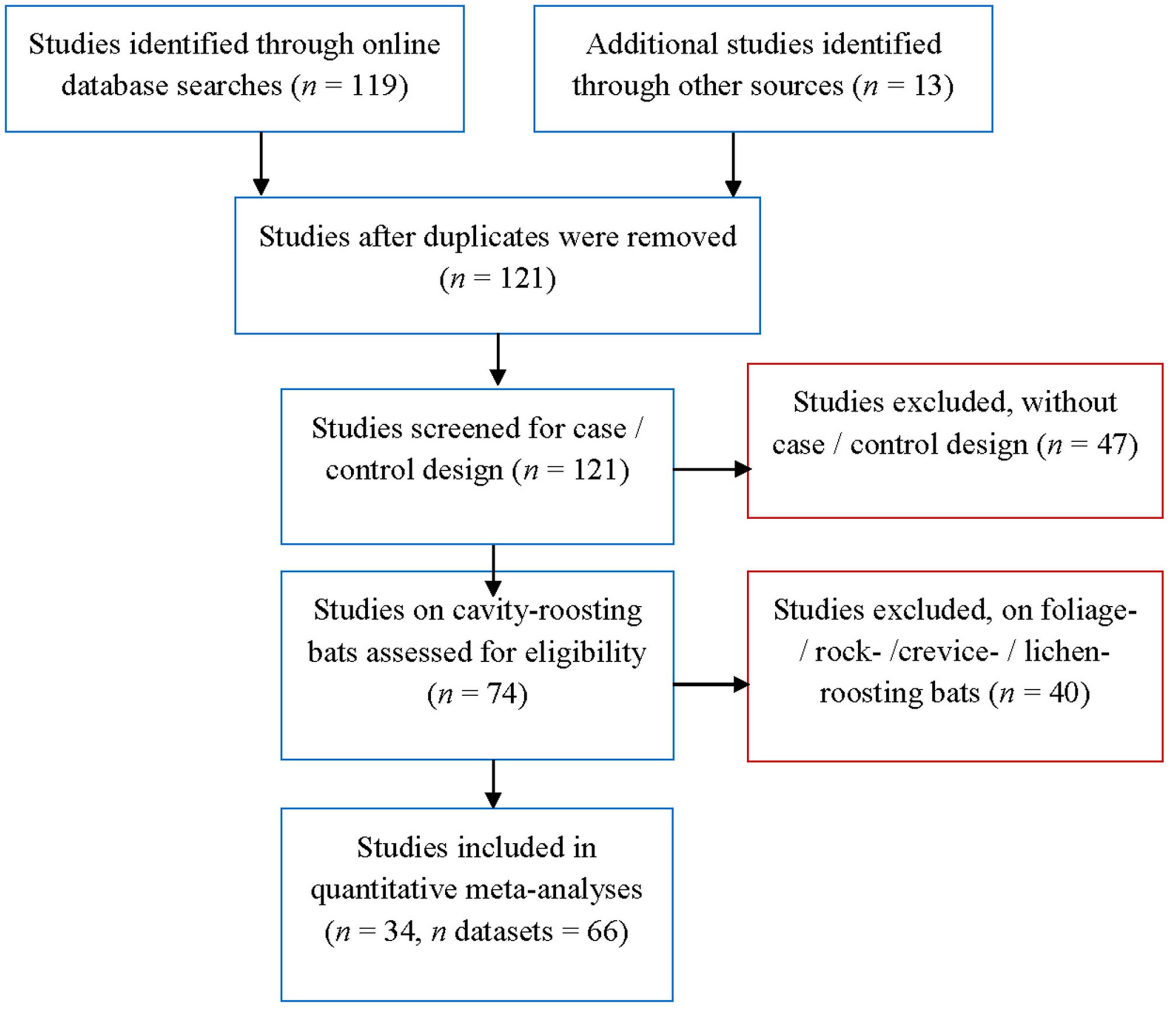
Flow diagram for identification and selection of studies of roost selection by North American bats for meta-analysis.

Of the 121 potential studies that we identified for roost selection by bats across North America, 74 studies compared roost trees that were selected by bats to random trees, and 40 of them studied rock-, lichen- or foliage-roosting bats (Fig 2). From this screening, we retained 34 studies on bark- and cavity-roosting bats for our meta-analysis, which corresponded to 66 datasets (Fig 2). We found 49 datasets in published manuscripts, 14 in unpublished Ph.D. theses, 2 in research symposia, and 1 in a governmental report. Datasets ranged from Prince of Wales Island in Alaska (northwest; Fig 1) to Baker County, on the Coastal Plain of Georgia (southeast; Fig 1). The datasets included 4 genera and 12 species of cavity-roosting bats: big brown bat (*Eptesicus fuscus*), silver-haired bat (*Lasionycteris noctivagans*), southeastern myotis (*M*. *austroriparius*), California bat (*M*. *californicus*), western long-eared bat (*M*. *evotis*), Keen’s myotis, little brown bat (*M*. *lucifugus*), northern long-eared bat, Indiana bat, fringed myotis (*M*. *thysanodes*), long-legged myotis (*M*. *volans*), and evening bat (*Nycticeius humeralis*). Most studies were performed on females (62.1%, *n* = 41), while 18.2% (*n* = 13) were conducted on males, and 19.7% (*n* = 12) combined both sexes. Bats using both snags and living trees as roosts represented 47% (*n* = 31) of the datasets, while 47% (*n* = 31) reported only snags, and 6% (*n* = 4) reported only living trees. Studies were mostly performed in managed areas (50%, *n* = 33), followed by 30.4% (*n* = 20) in protected areas, and 13.6% (*n* = 9) in riparian areas; 6% of the remaining datasets (*n* = 4) considered other treatment effects, such as fire (3%, *n* = 2) or vegetation types (3%, *n* = 2).

### Standardized mean differences

We found significant SMD for five of the nine characteristics (Table 1). Roost trees had significantly larger DBH (*n* = 66, SMD = 0.71, *P* < 0.0001) and were significantly taller (*n* = 47, SMD = 0.51, *P* < 0.0001) than random trees. Roost trees were mostly located in stands with a higher snag density (*n* = 34, SMD = 0.47, *P* < 0.0001), at a lower elevation (*n* = 19, SMD = -0.35, *P* < 0.0001), and with lower canopy closure (*n* = 33, SMD = -0.32, *P* = 0.006) compared to random stands. We found no significant difference between roost and random trees with respect to distance to water (*n* = 22, SMD = -0.16, *P* = 0.05), tree density (*n* = 24, SMD = 0.06, *P* = 0.58), slope (*n* = 21, SMD = 0.03, *P* = 0.78), and bark remaining on trunks (*n* = 26, SMD = 0.05, *P* = 0.78).

**Table 1:** Summary of the random effects meta-analysis of roost selection by North American bats, with heterogeneity indices and publication biases for each characteristic.

Characteristic	*K*	SMD	95% CI		*Z*	*P*	*τ* ^*2*^ *	I^2^ (%)	95% CI (%)		*t*	*df*	*P*
**Tree diameter**	63	0.71	0.57	0.86	9.87	<0.0001	0.24	0.76	0.70	0.81	1.84	61	0.07
**Tree height**	47	0.51	0.34	0.69	5.67	<0.0001	0.30	0.85	0.81	0.88	-0.84	45	0.40
**Number of snags**	34	0.47	0.33	0.62	6.49	<0.0001	0.11	0.69	0.55	0.78	-0.25	32	0.80
**Elevation**	19	-0.35	-0.51	-0.18	-4.11	<0.0001	0.07	0.58	0.31	0.75	-0.51	17	0.61
**Canopy closure**	33	-0.32	-0.54	-0.09	-2.77	0.006	0.36	0.83	0.77	0.87	-1.70	31	0.10
**Distance to water**	22	-0.16	-0.33	0.00	-1.95	0.05	0.10	0.68	0.50	0.79	1.61	20	0.12
**Tree density**	24	0.06	-0.15	0.27	0.55	0.58	0.20	0.76	0.64	0.84	2.13	22	0.05
**Slope**	21	0.03	-0.16	0.21	0.30	0.78	0.12	0.72	0.56	0.82	-0.50	19	0.62
**Bark remaining on trunks**	26	0.05	-0.31	0.41	0.28	0.78	0.80	0.96	0.95	0.97	-0.17	24	0.86

K = number of datasets and SMD = standardized mean difference; τ^2^ and I^2^ indices indicate the severity of between-studies heterogeneity; t-tests are for funnel-plot asymmetry, with associated degrees-of-freedom and P-values. All values are rounded upward to two decimal places.

* Estimated by maximum likelihood

### Publication bias and heterogeneity

**Fig 3:**
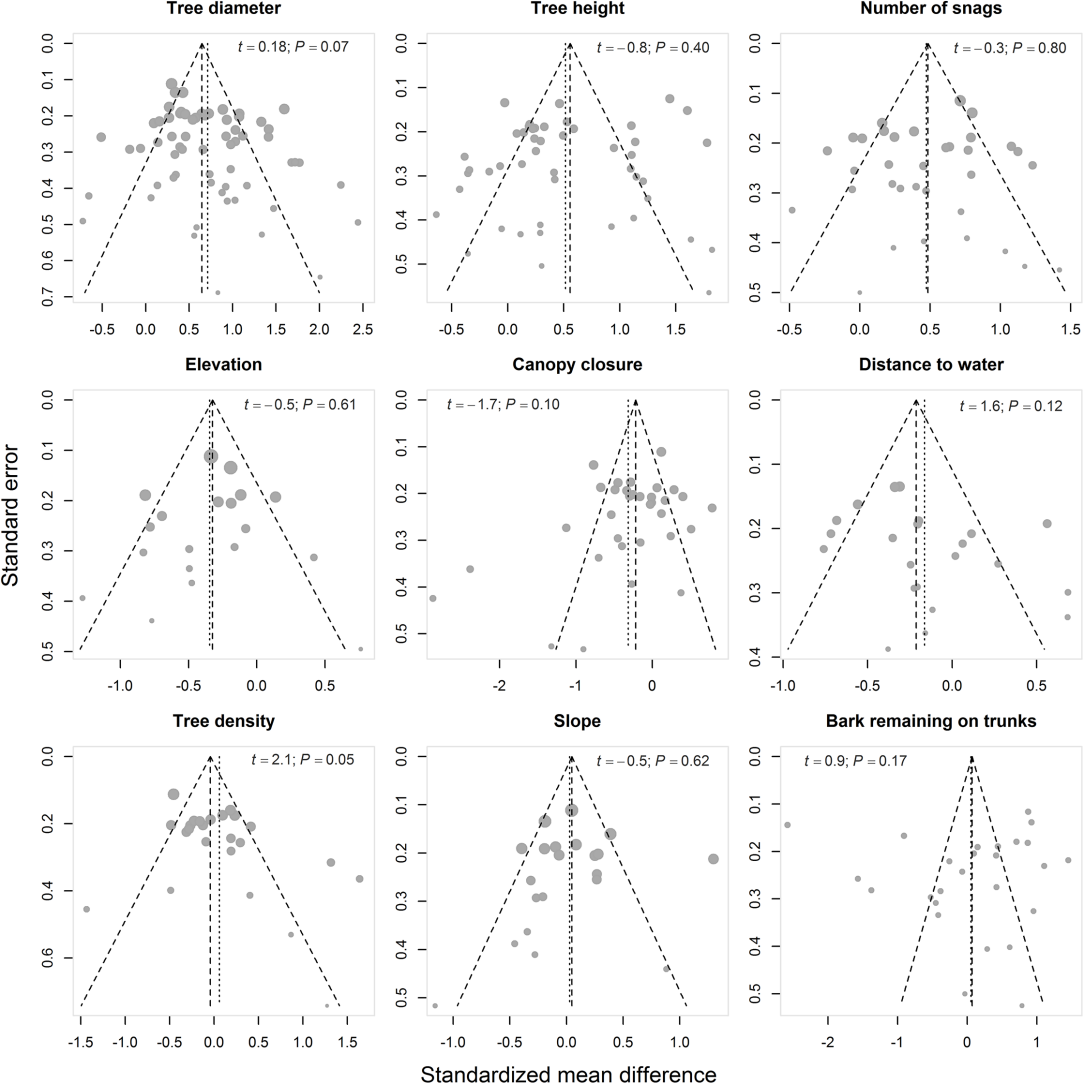
Funnel plots showing publication bias for each of the nine characteristics that were included in our quantitative meta-analysis. For each dataset, the effect size on the horizontal axis (standardized mean difference) is plotted against its standard error on the vertical axis. Dotted lines define the 95% CI limits around the mean effect size (vertical dotted line). The size of the circle varies according to the assigned random weight (inverse variance of the standardized mean differences) of each dataset. Funnel plot asymmetry *t*-test results and associated *P*-values are shown in each plot. In the absence of publication bias, studies should follow a symmetric funnel shape. Deviation from this shape may indicate publication bias.

**Fig 4:**
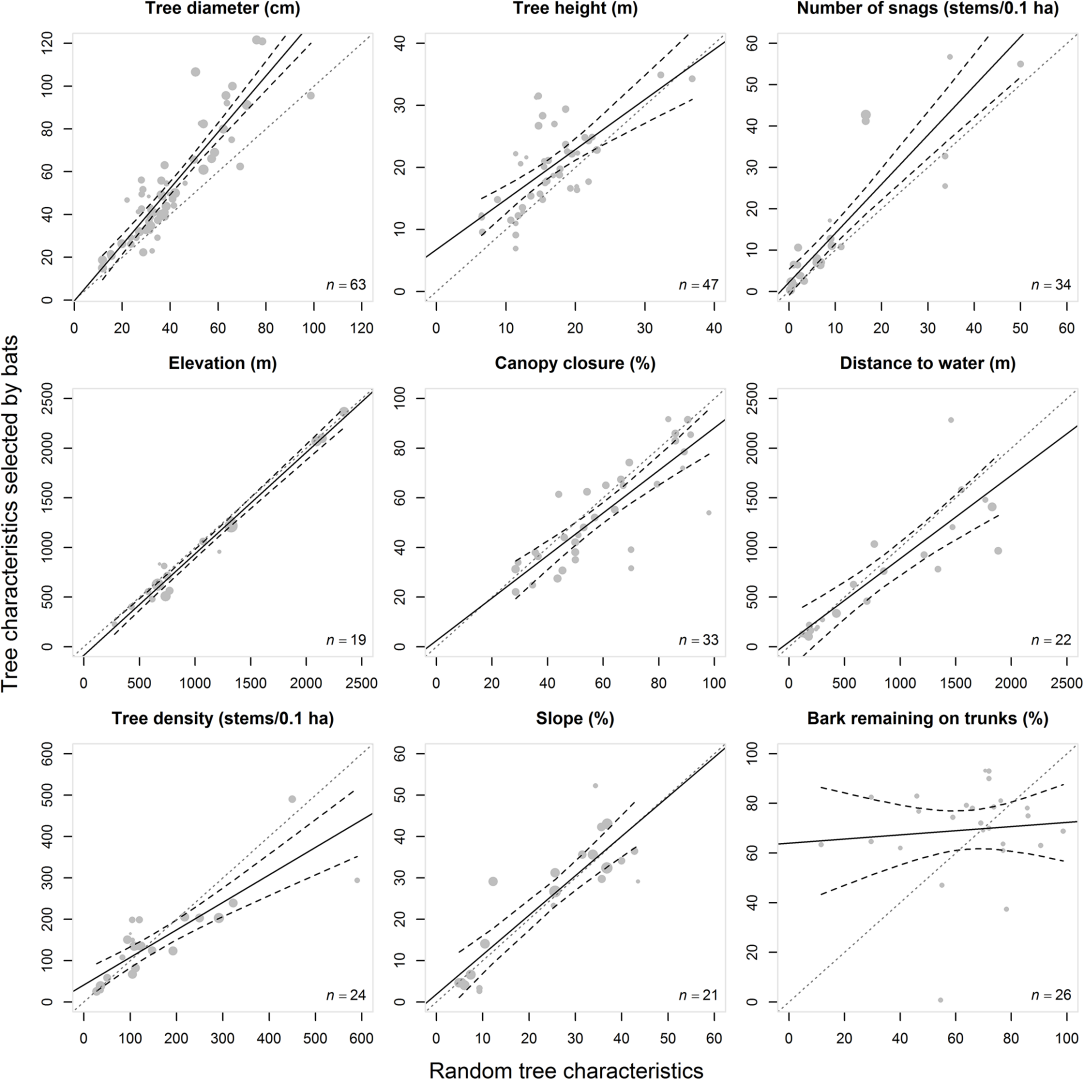
L’Abbé plots of the tree characteristics selected by bats (experimental groups) against the random tree characteristics (control group) with the 95% CI (black dashed lines) for each dataset, and for each characteristic (tree diameter, tree height, snag density, bark remaining on trunks, distance to water, canopy closure, elevation, slope, and stand density). The size of the circle varies according to the assigned random weight (inverse variance of the standardized mean differences) of each dataset. The diagonal (x = y) grey dotted line is the equality line (1:1) between both means (*i*.*e*., the zero effect line, for which the mean difference = 0). Above the x = y line, the experimental group mean is higher than the control group mean. Below the x = y line, the experimental group mean is lower than the control group mean. Tau-squared (*τ* ^2^) and Higgins’ I^2^ heterogeneity indices are shown in each plot. Higgins' I^2^ index is expressed in percentage and is used to interpret the severity of heterogeneity.

Funnel plots were well balanced (Fig 3); therefore, asymmetry tests did not reveal any significant publication bias (Table 1). Higgins’ I^2^ heterogeneity index indicated considerable levels of heterogeneity (*i*.*e*., I^2^ indices ranging from 50% to 100%) for each characteristic of roost selection by bats (Table 1; Fig 4).

### Spatial autocorrelation and meta-regressions

SMD values and squared residuals of our best regression model (*i*.*e*., tree diameter effect sizes vs. mean summer temperature) were not spatially autocorrelated. Moran’s I test for spatial autocorrelation did not reject the null hypothesis of spatial randomness either for our SMD values (Moran I standard deviate = -0.29, *P* = 0.62) or for our best regression model residuals (Moran I standard deviate = -1.07, *P* = 0.28).

According to ∆AICc (Table 2), the two meta-regression models that best explained heterogeneity in tree diameter effect sizes, included (i) mean summer temperature (pseudo-*R* ^*2*^ = 13.26%; AICc *ω* = 0.42) and (ii) mean summer temperature + sex (pseudo-*R* ^2^ = 17.40%; AICc *ω* = 0.19) as moderator variables. Tree diameter effect sizes for female bats increased significantly with decreasing mean summer temperature and increasing latitude. However, elevation (pseudo-*R* ^2^ = 1.94%; AICc *ω* = 0.02), sex (pseudo-*R* ^2^ = 2.58%; AICc *ω* = 0.02) and habitat type (pseudo-*R* ^2^ = 4.34%; AICc *ω* = 0), alone had little effect on heterogeneity in tree diameter effect sizes. These single predictor models were ranked among the poorest AICc models (Table 2).

**Table 2:** Meta-regression model number, number of estimated parameters (K), pseudo-R^2^ (ps-R^2^) estimating the amount of heterogeneity (%) accounted for by each model, differences between model AICc and those of the best model (*∆*_*i*_), and Akaike weights (*ω*_*i*_), for 17 meta-regression models.

#	Meta-regression model	*K*	ps-*R* ^2^	Δ_*i*_	*ω*_*i*_
13	Temperature	3	13.26	0.00	0.43
10	Temperature + sex	5	17.40	1.58	0.20
12	Temperature + elevation	4	13.31	2.24	0.14
7	Temperature + elevation + sex	6	17.46	3.96	0.06
11	Temperature + elevation + temperature x elevation	5	13.93	4.24	0.05
16	Bat species	7	20.08	5.87	0.02
9	Temperature + bat species	8	23.11	5.97	0.02
14	Elevation	3	1.94	6.16	0.02
15	Bat species + sex	9	26.28	6.30	0.02
17	Sex	4	2.58	6.67	0.02
8	Temperature + bat species + sex	10	29.09	7.15	0.01
6	Temperature + bat species + elevation	9	23.11	8.69	0.01
5	Temperature + elevation + bat species + sex	11	29.19	10.00	0.00
1	Habitat type + Temperature + elevation + sex	11	23.32	13.31	0.00
4	Habitat type	7	4.34	14.86	0.00
3	Habitat type + elevation	8	6.75	16.12	0.00
2	Habitat type + elevation + sex	10	11.09	17.41	0.00

All values are rounded upward to two decimal places.

The model that explained the most heterogeneity in tree diameter effect sizes (pseudo-*R* ^2^ = 29.19%) included mean summer temperature, elevation, bat species and sex as moderator variables. This model had a high ∆AICc (*∆*AICc = 10; AICc *ω* = 0), compared to the two best AICc models (*i*.*e*., with ∆AICc = 0 and = 1.58), which included only mean summer temperature and sex as moderator variables.

## Discussion

### Meta-analysis and heterogeneity

Our meta-analysis included a larger number of characteristics, and increased the scope to a wider range of bat species and forest habitats throughout North America than previous quantitative reviews [1, 7, 65]. Despite an overall high level of heterogeneity among studies, five characteristics showed strong general trends in roost selection by bats. Cavity-roosting bats selected larger and taller roosts compared to random trees. They also roosted in stands with a larger number of surrounding snags, at lower elevations, and with less canopy closure compared to random stands. These results are consistent with those found by Lacki and Baker [65], and Kalcounis-Rueppell, Psyllakis [1]. Other characteristics, such as distance to water, slope, and bark remaining on trunks, did not significantly differ from random trees because of strong differences in size and direction of effects among studies. With respect to distance to water, our results slightly differed from those of Kalcounis-Rueppell, Psyllakis [1], since we included a larger set of studies [59, 72, 86] with a positive effect size (*i*.*e*., random trees that were closer to water). Water is an important resource for bats [33–35], especially in arid regions [34, 35]. Only two studies included in our meta-analysis were located in arid regions and reported distance to water [87, 88]. It would be interesting to investigate if studies located in arid areas show roosts being at shorter distances to water than studies where the availability of water to bats and precipitation are important.

### Characteristics likely related to temperature

We found considerable heterogeneity in slope effect sizes. Further, it was difficult to identify a general trend from the literature, since slope appears to be related to the topographical context of the study. Unlike slope, we found greater consistency between results from different studies for elevation. Heterogeneity in elevation was even the lowest compared to the other characteristics explaining roost selection that we tested. Studies were conducted at a specific elevation (*i*.*e*., where roosts and random trees are in the same elevation zone), and short distances between roosts and matching random trees are typically taken in the field [64], which likely minimized the effect size for this characteristic. Despite the fact that studies are conducted at a specific elevation, we showed that elevation differences between selected and random trees is a consistent pattern among studies. Bats might select trees located at lower elevations to benefit from warmer microclimate and greater insect availability near roosts, relative to trees that are located at higher elevations [89]. Several studies found sexual segregation in bats with reproductive females less likely to occur in stands at higher elevation [90]. Russo [91] and Arnold [92] obtained similar results with Daubenton’s bat (*Myotis daubentonii*) and the northern long-eared bat, respectively. Cryan, Bogan [93] showed an inverse relationship between habitat elevation and the presence of reproductive females in South Dakota.

### Tree decay and bark remaining on trunks

Most bat species that we included in our meta-analysis seek shelter inside trunk cavities [7, 72] and under the exfoliating bark of snags with an intermediate stage of decay [16, 20, 56, 58]. Only 3 studies have reported the exclusive use of cavities within living trees [3, 59, 60] and two of these were associated with southeastern myotis [59, 60]. Although bark remaining on trunks was the most heterogeneous characteristic among those that we studied, a clear preference was exhibited by bats towards snags with about 70% of bark remaining on trunks (Fig 4). An intermediate stage of decay should offer the best compromise between an appropriate tree height and enough bark remaining on the trunk to provide a roost [2, 3]. Another interesting aspect of snags is that they offer less buffering capacity against external temperature variation, compared to living trees [42, 94]. However, they likely provide more available cavities [95] compared to living trees. Thus, selection of roosts by bats might be driven by a trade-off between the availability of potential roost trees in a given stand [59], their related benefits in terms of warm microclimates, and their relatively short distances to feeding sites [96]. More studies are clearly needed to better understand the thermal capacity of trees and its implications in bat behaviour [41, 48].

### Moderator variables and tree diameter effect sizes

Tree diameter was the strongest characteristic explaining roost selection by cavity-roosting bats, since positive effect sizes (*i*.*e*., trees selected with a larger diameter than random trees) were a common finding in several studies [6, 20, 59, 72]. The main hypothesis invoked by these studies was that trees with a larger diameter offered greater thermal inertia against external temperature variation [41, 42, 94, 97], compared to trees with a smaller diameter. For reproductive female bats, the importance of stable and warm temperatures has been discussed in detail by Barclay and Kurta [5]. Reproductive females are thought to benefit from warm and stable microclimates that minimize thermoregulation costs and which maximize their fitness [5, 47]. However, these assumptions have rarely been tested empirically in North American bat research [48]. Most studies that have measured temperature variation in roosts of bats and other mammals have been conducted in Europe [44, 45, 98] and New Zealand [41, 99, 100]. To our knowledge, only Park and Broders [40], have shown reductions in temperature fluctuations within roosts that were used by lactating northern long-eared bats in Newfoundland. Lacki, Johnson [43] also showed reductions in temperature fluctuations within roosts that were used by long-legged myotis and which were located beneath the exfoliating bark of trees, in Idaho and Oregon.

Surprisingly, moderator variables such as elevation, bat species, and habitat types were not included in our best model explaining heterogeneity in tree diameter effect sizes. When sex was combined with mean summer temperature, the two predictors explained further heterogeneity. Otherwise, sex alone performed poorly. Subsequent tests for subgroup differences indicated that intra-study heterogeneity for female bats was greater than inter-study heterogeneity, when considering all groups (*i*.*e*., males, females and combined). The variability that could be attributed to sex, although present [36, 40], was masked by other moderator variables having a greater influence on tree diameter effect sizes. It was interesting to note that the model explaining the most heterogeneity included mean summer temperature, elevation, bat species, and sex as moderator variables. This model had a lower AICc ranking since it was less parsimonious (*i*.*e*., *K* = 11 parameters to estimate) than the two best models [101, 102], which include only mean summer temperature (*K* = 3) and mean summer temperature + sex (*K* = 5) as moderator variables.

Mean summer temperature and sex were the two moderator variables that best explained heterogeneity in tree diameter effect sizes. Our main finding was that, in the case of female bats, regional differences in selection for tree diameter were correlated to mean summer temperatures of the location where the studies were performed. In northern regions with lower mean summer temperatures, female bats showed greater selectivity towards large trees, compared to southern regions, which benefit from higher mean summer temperatures [72].

This study confirmed a relation between regional differences in roost selection by bats and differences in the climatic conditions (*i*.*e*., temperature) occurring across a broad spatial scale [7]. Most of the studies that we included in our analyses were from the Pacific Northwest of the US, the southeastern US, and southeastern Canada/northeastern US. Although the studies within these three regions appeared clustered (Fig 1), SMD estimates from these studies were not spatially dependent. In light of these results, the challenge of retaining trees with large diameters seems critical to ensuring the survival of bats, particularly in northern and mountainous regions with low mean summer temperatures and short growing seasons [2, 72].

### Limitations and research perspective

We expected a high degree of heterogeneity because the studies that we included in our meta-analysis were conducted in various habitats, had included numerous bat species, and attempted to answer different questions. Despite the inclusion of moderator variables, most heterogeneity in tree diameter effect sizes remained unexplained. We are aware that we have used a relatively coarse measure of daily summer temperature that likely underscored regional temperature fluctuations. More accurate moderator variables could likely capture more heterogeneity in tree diameter effect sizes. It is likely that the differences in results among studies were also influenced by measurement methods [64]. We agree with Miller, Arnett [64] that random sites that are located in close proximity to selected roosts by bats might increase the lack of independence, and therefore, minimize the true effect sizes for several distance-based characteristics, such as elevation and distance to water. We were not able to estimate this potential bias since the authors rarely mentioned distances between trees that were selected by bats and random trees. Including this information in future research should greatly improve the interpretation of the results.

Ambient temperature, exposure to solar radiation, and thermal properties of trees appear to play a central role in roost selection by bats. These aspects of the roost microclimate hypothesis, as described by Boyles [46], have been rarely investigated and should be included in future research. Driven by a forest management perspective, the majority of studies have focused their research on tree and stand characteristics (*e*.*g*., tree diameter, tree height, density of trees and canopy closure) that provide indirect links to microclimate. Studies that we reviewed also rarely mentioned stand age, although it may be correlated with the most important covariates of roost selection, such as tree diameter and tree height [103, 104], canopy closure [105], tree density [106], snag density [18, 107], and the number of available cavities [108]. The lack of published studies and available reports in northern Canada, in the desert southwest and the Midwest-West prairies in the US, and in Mexico has also limited our analyses to the southeastern US, the US Pacific Northwest and southeastern Canada/northeastern US. It would be interesting to include studies on roost selection by bats that were performed in northern regions to challenge our hypothesis.

## Supporting Information

S1 Table Meta-analysis on diameter at breast height (cm). **Number of selected and random trees is provided for each dataset with corresponding mean, standard deviation (SD), standardized mean difference (SMD) with 95% CI, fixed weight (W), and random weight. Fixed effect and random effects SMD with 95% CI, and prediction intervals are provided at the end of the table**. All values are rounded upward to two decimal places.
(DOCX)

S2 Table Meta-analysis on tree height (m). **Number of selected and random trees is provided for each dataset with corresponding mean, standard deviation (SD), standardized mean difference (SMD) with 95% CI, fixed weight (W), and random weight. Fixed effect and random effects SMD with 95% CI, and prediction intervals are provided at the end of the table.** All values are rounded upward to two decimal places.
(DOCX)

S3 Table Meta-analysis on snag density (stems/0.1 ha). **Number of selected and random trees is provided for each dataset with corresponding mean, standard deviation (SD), standardized mean difference (SMD) with 95% CI, fixed weight (W), and random weight. Fixed effect and random effects SMD with 95% CI, and prediction intervals are provided at the end of the table.** All values are rounded upward to two decimal places.
(DOCX)

S4 Table Meta-analysis on elevation (m). **Number of selected and random trees is provided for each dataset with corresponding mean, standard deviation (SD), standardized mean difference (SMD) with 95% CI, fixed weight (W), and random weight. Fixed effect and random effects SMD with 95% CI, and prediction intervals are provided at the end of the table.** All values are rounded upward to two decimal places.
(DOCX)

S5 Table Meta-analysis on canopy closure (%). **Number of selected and random trees is provided for each dataset with corresponding mean, standard deviation (SD), standardized mean difference (SMD) with 95% CI, fixed weight (W), and random weight. Fixed effect and random effects SMD with 95% CI, and prediction intervals are provided at the end of the table.** All values are rounded upward to two decimal places.
(DOCX)

S6 Table Meta-analysis on distance to water (m). **Number of selected and random trees is provided for each dataset with corresponding mean, standard deviation (SD), standardized mean difference (SMD) with 95% CI, fixed weight (W), and random weight. Fixed effect and random effects SMD with 95% CI, and prediction intervals are provided at the end of the table.** All values are rounded upward to two decimal places.
(DOCX)

S7 Table Meta-analysis tree density (%). **Number of selected and random trees is provided for each dataset with corresponding mean, standard deviation (SD), standardized mean difference (SMD) with 95% CI, fixed weight (W), and random weight. Fixed effect and random effects SMD with 95% CI, and prediction intervals are provided at the end of the table**. All values are rounded upward to two decimal places.
(DOCX)

S8 Table Meta-analysis on slope (%). **Number of selected and random trees is provided for each dataset with corresponding mean, standard deviation (SD), standardized mean difference (SMD) with 95% CI, fixed weight (W), and random weight. Fixed effect and random effects SMD with 95% CI, and prediction intervals are provided at the end of the table.** All values are rounded upward to two decimal places.
(DOCX)

S9 Table Meta-analysis on bark remaining on trunks (%). **Number of selected and random trees is provided for each dataset with corresponding mean, standard deviation (SD), standardized mean difference (SMD) with 95% CI, fixed weight (W), and random weight. Fixed effect and random effects SMD with 95% CI, and prediction intervals are provided at the end of the table.** All values are rounded upward to two decimal places.
(DOCX)
