# Expression of Concern: Exploring Regional Variation in Roost Selection by Bats: Evidence from a Meta-Analysis

**DOI:** 10.1371/journal.pone.0316243

**Published:** 2024-12-18

**Authors:** The PLOS ONE Editors

Following publication of this article [1], concerns were raised about reporting of the methodology and discrepancies in the datasets. After follow up with the corresponding author and consultation with an expert member of the Editorial Board and a statistical reviewer, the *PLOS ONE* Editors received input that the issues do not affect the main conclusions; however, some items could not be fully resolved.

**Dataset errors**

The corresponding author stated that the originally published datasets used in the meta-analyses contained errors which arose when a cleaned dataset was accidentally overwritten with an older version. They noted that reanalysis of the corrected data produced minor changes to the results, with the main conclusions remaining supported. After reanalysis, one additional factor reaches statistical significance (distance to water). The revised meta-analyses are provided here in S1 File, along with detailed descriptions of the corrections made to the datasets in S2 File. Corrected versions of Table 1, Fig 3 and Fig 4 are also provided in S1 File. A consulted member of the Editorial Board and statistical reviewer advised that the revised results are consistent with the original conclusions and the numerical changes to the results are minor. However, one discrepancy was noted that was not addressed in the revised dataset. Specifically:

- In the corrected meta-analysis for tree diameter (S1 File), sample sizes for the Perry and Thill (2007) study are reported as 40 and 42, while the source article reports sample sizes of 43 and 49.

The statistical reviewer noted that this remaining discrepancy is unlikely to affect the results.

Readers are advised there appear to be discrepancies in some descriptions of the revisions in S2 File, as follows:

- For the analysis of Canopy Closure, the corrected mean value from Lacki et al. 2009 used in the revised analyses (S1 File) is 47% (revised from 45%), but these values are reversed in the description in S5_Table_correction report (S2 File).
- For the analysis of Canopy Closure, the revised Table 1 in S1 File reports an updated P value of P < 0.0001 for the random effects model, while the S5_Table_correction report (S2 File) reports a revised P value of P = 0.0027 for the random effects model and P <0.0001 for the fixed effect model.
- For the analysis of Tree Density, the mean (SD) values from the Vonhof and Gwilliam studies used in the revised analyses (S1 File and S7_Table_correction report in S2 File) are 38 (28.4), and 38 (30.9) for big brown bats (EPFU) and California bat (MYCA), respectively, but the accompanying description in S7_Table_correction report in S2 File gives values of 46 (38) and 20 (38).
- For the analysis of Slope, the mean converted angle value from the Rabe et al. (1998) study used in the revised analysis (S1 File and S8_Table_correction report in S2 File) is 23.1, but the accompanying description in S8_Table_correction report in S2 File gives a value of 26.1.

**Article updates**

Based on the results of the revised analyses, the following parts of the article text are amended:

Abstract

The revised text for the Results subsection of the Abstract is:

“Tree diameter, tree height, snag density, elevation, canopy closure, and distance to water were significant characteristics of roost selection by cavity-roosting bats. Size and direction of effects varied greatly among studies with respect to tree density, slope, and bark remaining on trunks. Inclusion of mean summer temperature and sex in meta-regressions further explained heterogeneity in tree diameter effect sizes.

Results

The revised text for the Standardized mean differences subsection of the Results section is as follows:

“We found significant SMD for six of the nine characteristics (Table 1). Roost trees had significantly larger DBH and were significantly taller than random trees. Roost trees were mostly located in stands with a higher snag density, at a lower elevation, and with lower canopy closure and lower distance to water compared to random stands. We found no significant difference between roost and random trees with respect to tree density, slope, and bark remaining on trunks.”

For details of specific results, readers should refer to the revised analyses in S1 File and S2 File, and take into account the information about dataset errors detailed in the above section of this notice.

Discussion

The revised text for the Meta-analysis and heterogeneity subsection of the Discussion section is:

“Our meta-analysis included a larger number of characteristics, and increased the scope to a wider range of bat species and forest habitats throughout North America than previous quantitative reviews [1, 7, 65]. Despite an overall high level of heterogeneity among studies, six characteristics showed strong general trends in roost selection by bats. Cavity-roosting bats selected larger and taller roosts compared to random trees. They also roosted in stands with a larger number of surrounding snags, at lower elevations, with less canopy closure and a lower distance to water compared to random stands. These results are consistent with those found by Lacki and Baker [65], and Kalcounis-Rueppell, Psyllakis [1]. Other characteristics, such as slope, and bark remaining on trunks, did not significantly differ from random trees because of strong differences in size and direction of effects among studies. Water is an important resource for bats [33–35], especially in arid regions [34, 35]. Only two studies included in our meta-analysis were located in arid regions and reported distance to water [87, 88]. It would be interesting to investigate if studies located in arid areas show roosts being at shorter distances to water than studies where the availability of water to bats and precipitation are important.”

**Additional issues**

In the Spatial autocorrelation and meta-regressions subsection of the Results section, specific values cited in the text should be disregarded as revisions have not been provided to fully address all instances of incorrect specific values. However, the corresponding author has indicated that the broad results and conclusions in this section remain supported. Specifically, a Moran’s I test showed no spatial correlation, and an updated version of the delta AICc model ranking analysis is provided in S2 File (in S1_Table correction_report), in which some values are revised but the best models are unchanged compared to the originally published Table 2 (Temperature, Temperature + sex, Temperature + elevation).

Methodology

The Selection of studies subsection of the Materials and Methods section does not report the specific search terms or strings used to retrieve studies from Google Scholar and Web of Science. The corresponding author has clarified that the search terms included the keywords "bat" "roost" "selection" "selected" "cavity" "roosting" "tree" and "snag" in several combinations, but the precise combinations were not documented. The consulting Editorial Board member advised that the reporting of the methodology does not meet best practice standards for systematic reviews and meta-analyses. The *PLOS ONE* Editors consider the information reported as insufficient to allow the study to be reproduced or to allow assessment of the validity of the search methodology.

Some additional methodological clarifications have been provided by the corresponding author as follows:

- Studies that reported different measurements for number of snags or snag density were converted to number of snags per 0.1 ha where possible; original values were kept when it was not possible to convert the data. This approach is discussed further in the S3 Table correction report in S2 File.
- The Materials and Methods section states that “We excluded studies with an effect size greater than 4 times the mean group standard deviation to meet criteria of effect size normality and variance homogeneity.” No sensitivity analyses were performed for results with and without including these studies because all effect sizes fell below the exclusion threshold, and no studies were discarded for this reason.
- With regard to the random effects models, each effect size (*n*) is considered a sampling unit (including those from the same study), and an effect-size-level random effect has been applied to each effect size (*n*). There were studies that contributed to more than one effect size (mentioned as “*dataset*” in the paper); the analysis had an effect size-level random effect nested within a study-level effect that quantified within- and between-study heterogeneity as suggested in references 66 and 68 of [1].

Most errors in the dataset are resolved by the reanalyses and article updates provided in this notice and the accompanying supporting information files, and expert input supports that the main results and conclusions are not affected. However, the discrepancy involving the Perry and Thill (2007) data and issues discussed above in the “Additional issues” section were not fully resolved. Readers are furthermore advised that in editorial follow-up, the values in the revised dataset provided by the corresponding author have not been fully cross-checked by PLOS against the original source data from all primary studies. The *PLOS ONE* Editors issue this Expression of Concern to inform readers of the above updates and unresolved issues.

## Supporting information

S1 File Revised results. Revised meta-analyses, Table 1, and Figs 3–4
(PDF)

S2 File File Correction reports for Supporting Information Tables S1-S9. These files provide clarifications regarding sources, extraction and conversion of data; and descriptions of errors and their corrections provided by the corresponding author. Readers should also refer to the Expression of Concern notice section on dataset errors.
(ZIP)
