# Supplementary material for: Exploring Regional Variation in Roost Selection by Bats: Evidence from a Meta-Analysis
Source: PLoS One. 2015 Sep 29;10(9):e0139126. doi: 10.1371/journal.pone.0139126 (PMC4587962; doi:10.1371/journal.pone.0139126)
Supplement: S1 Table — Number of selected and random trees is provided for each dataset with corresponding mean, standard deviation (SD), standardized mean difference (SMD) with 95% CI, fixed weight (W), and random weight. Fixed effect and random effects SMD with 95% CI, and prediction intervals are provided at the end of the table. All values are rounded upward to two decimal places. (DOCX) [file pone.0139126.s001.docx]

# Supporting information 1

## S1 Table. Meta-analysis on diameter at breast height (cm). Number of selected and random trees is provided for each dataset with corresponding mean, standard deviation (SD), standardized mean difference (SMD) with 95 % CI, fixed weight (W), and random weight. Fixed effect and random effects SMD with 95 % CI, and prediction intervals are provided at the end of the table. All values are rounded upward to two decimal places.

|  | **Selected trees** | | | **Random trees** | | |  |  |  |  |
| --- | --- | --- | --- | --- | --- | --- | --- | --- | --- | --- |
| **Study** | ***N*** | **Mean** | **SD** | ***N*** | **Mean** | **SD** | **SMD** | **95 % CI** | **W(fixed)** | **W(random)** |
| [[1](#_ENREF_1)] | 42 | 121.6 | 42.8 | 104 | 76.1 | 41.8 | 1.08 | 0.70; 1.46 | 2.6 % | 1.9 % |
| [[1](#_ENREF_1)] | 35 | 138.7 | 47.9 | 33 | 96.8 | 41.4 | 0.92 | 0.42; 1.43 | 1.5 % | 1.7 % |
| [[1](#_ENREF_1)] | 24 | 62.4 | 37.2 | 23 | 69.2 | 35.5 | -0.18 | -0.76; 0.39 | 1.1 % | 1.6 % |
| [[1](#_ENREF_1)] | 105 | 91.1 | 49.2 | 119 | 72.1 | 39.3 | 0.43 | 0.16; 0.69 | 5.3 % | 2.0 % |
| [[1](#_ENREF_1)] | 22 | 95.5 | 50.7 | 26 | 98.7 | 54.6 | -0.06 | -0.63; 0.51 | 1.2 % | 1.6 % |
| [[2](#_ENREF_2)] | 164 | 60.9 | 24.3 | 160 | 54.0 | 21.5 | 0.30 | 0.08; 0.52 | 7.8 % | 2.1 % |
| [[2](#_ENREF_2)] | 28 | 82.3 | 19.1 | 160 | 54.0 | 21.5 | 1.33 | 0.91; 1.76 | 2.1 % | 1.8 % |
| [[3](#_ENREF_3)] | 1 | 56.0 | 16.8 | 38 | 28.0 | 15.0 | 1.77 | 1.12; 2.42 | 0.9 % | 1.5 % |
| [[4](#_ENREF_4)] | 48 | 32.9 | 10.4 | 48 | 30.0 | 11.1 | 0.27 | -0.13; 0.67 | 2.3 % | 1.9 % |
| [[4](#_ENREF_4)] | 55 | 43.8 | 13.4 | 55 | 38.3 | 14.1 | 0.40 | 0.02; 0.78 | 2.6 % | 1.9 % |
| [[4](#_ENREF_4)] | 57 | 32.0 | 12.1 | 57 | 27.2 | 11.3 | 0.41 | 0.04; 0.78 | 2.7 % | 1.9 % |
| [[5](#_ENREF_5)] | 7 | 49.3 | 16.4 | 8 | 40.0 | 15.0 | 0.56 | -0.48; 1.60 | 0.3 % | 1.0 % |
| [[5](#_ENREF_5)] | 11 | 54.5 | 15.6 | 14 | 38.5 | 14.6 | 1.03 | 0.18; 1.88 | 0.5 % | 1.2 % |
| [[5](#_ENREF_5)] | 20 | 54.6 | 13.9 | 5 | 46.2 | 14.0 | 0.59 | -0.41; 1.58 | 0.4 % | 1.1 % |
| [[6](#_ENREF_6)] | 19 | 37.3 | 20.6 | 46 | 34.9 | 15.0 | 0.14 | -0.39; 0.68 | 1.3 % | 1.7 % |
| [[6](#_ENREF_6)] | 47 | 39.0 | 14.0 | 37 | 37.7 | 13.6 | 0.09 | -0.34; 0.53 | 2.0 % | 1.8 % |
| [[7](#_ENREF_7)] | 25 | 95.5 | 30.0 | 314 | 63.3 | 34.7 | 0.93 | 0.52; 1.35 | 2.2 % | 1.9 % |
| [[8](#_ENREF_8)] | 8 | 42.1 | 7.4 | 30 | 31.7 | 12.4 | 0.88 | 0.07; 1.69 | 0.6 % | 1.3 % |
| [[8](#_ENREF_8)] | 21 | 39.0 | 8.2 | 30 | 31.7 | 12.4 | 0.66 | 0.09; 1.23 | 1.1 % | 1.6 % |
| [[8](#_ENREF_8)] | 9 | 43.3 | 12.1 | 30 | 31.7 | 12.4 | 0.92 | 0.15; 1.70 | 0.6 % | 1.3 % |
| [[8](#_ENREF_8)] | 7 | 42.6 | 3.2 | 30 | 31.7 | 12.4 | 0.94 | 0.09; 1.79 | 0.5 % | 1.2 % |
| [[9](#_ENREF_9)] | 8 | 48.4 | 8.7 | 8 | 31.0 | 7.6 | 2.01 | 0.74; 3.27 | 0.2 % | 0.8 % |
| [[9](#_ENREF_9)] | 40 | 20.7 | 4.5 | 40 | 15.5 | 5.4 | 1.03 | 0.57; 1.50 | 1.7 % | 1.8 % |
| [[10](#_ENREF_10)] | 8 | 74.9 | 28.9 | 157 | 65.7 | 26.3 | 0.35 | -0.36; 1.06 | 0.7 % | 1.4 % |
| [[10](#_ENREF_10)] | 7 | 92.0 | 42.9 | 147 | 63.7 | 23.0 | 1.17 | 0.40; 1.94 | 0.6 % | 1.3 % |
| [[11](#_ENREF_11)] | 29 | 63.0 | 32.3 | 46 | 37.7 | 12.8 | 1.12 | 0.62; 1.62 | 1.5 % | 1.7 % |
| [[11](#_ENREF_11)] | 23 | 41.0 | 4.8 | 46 | 37.7 | 12.8 | 0.30 | -0.20; 0.80 | 1.5 % | 1.7 % |
| [[12](#_ENREF_12)] | 8 | 29.1 | 7.4 | 241 | 23.6 | 7.5 | 0.74 | 0.03; 1.44 | 0.7 % | 1.4 % |
| [[12](#_ENREF_12)] | 8 | 29.1 | 7.4 | 84 | 25.9 | 10.1 | 0.32 | -0.41; 1.05 | 0.7 % | 1.4 % |
| [[13](#_ENREF_13)] | 52 | 79.9 | 21.6 | 61 | 62.2 | 31.2 | 0.65 | 0.27; 1.02 | 2.6 % | 1.9 % |
| [[14](#_ENREF_14)] | 16 | 41.4 | 10.2 | 6 | 26.6 | 11.9 | 1.34 | 0.30; 2.37 | 0.3 % | 1.0 % |
| [[15](#_ENREF_15)] | 15 | 42.6 | 14.7 | 52 | 28.1 | 5.8 | 1.68 | 1.03; 2.32 | 0.9 % | 1.5 % |
| [[15](#_ENREF_15)] | 11 | 49.5 | 19.2 | 52 | 28.1 | 5.8 | 2.25 | 1.48; 3.01 | 0.6 % | 1.3 % |
| [[16](#_ENREF_16)] | 56 | 47.3 | 16.7 | 16 | 41.0 | 12.5 | 0.39 | -0.17; 0.95 | 1.2 % | 1.6 % |
| [[17](#_ENREF_17)] | 6 | 82.6 | 26.2 | 50 | 52.8 | 19.2 | 1.47 | 0.58; 2.36 | 0.5 % | 1.2 % |
| [[18](#_ENREF_18)] | 12 | 29.2 | 5.5 | 12 | 34.7 | 10.1 | -0.65 | -1.48; 0.17 | 0.5 % | 1.3 % |
| [[19](#_ENREF_19)] | 111 | 50.0 | 19.0 | 111 | 42.1 | 26.3 | 0.34 | 0.08; 0.61 | 5.3 % | 2.0 % |
| [[19](#_ENREF_19)] | 57 | 26.3 | 9.8 | 57 | 19.9 | 7.6 | 0.73 | 0.35; 1.11 | 2.6 % | 1.9 % |
| [[20](#_ENREF_20)] | 33 | 100.0 | 34.5 | 66 | 66.0 | 16.3 | 1.41 | 0.95; 1.88 | 1.7 % | 1.8 % |
| [[21](#_ENREF_21)] | 6 | 49.6 | 12.7 | 4 | 39.3 | 8.0 | 0.83 | -0.52; 2.18 | 0.2 % | 0.7 % |
| [[22](#_ENREF_22)] | 40 | 15.0 | 8.2 | 82 | 11.8 | 6.3 | 0.45 | 0.07; 0.84 | 2.5 % | 1.9 % |
| [[22](#_ENREF_22)] | 42 | 18.7 | 6.5 | 82 | 11.8 | 6.3 | 1.07 | 0.68; 1.47 | 2.4 % | 1.9 % |
| [[23](#_ENREF_23)] | 19 | 26.1 | 7.2 | 25 | 23.6 | 7.3 | 0.34 | -0.26; 0.94 | 1.0 % | 1.6 % |
| [[23](#_ENREF_23)] | 17 | 48.2 | 10.5 | 21 | 37.0 | 11.7 | 0.98 | 0.30; 1.66 | 0.8 % | 1.5 % |
| [[24](#_ENREF_24)] | 43 | 66.0 | 11.8 | 58 | 57.4 | 16.8 | 0.57 | 0.17; 0.98 | 2.3 % | 1.9 % |
| [[24](#_ENREF_24)] | 54 | 69.0 | 14.7 | 54 | 58.6 | 15.4 | 0.69 | 0.30; 1.07 | 2.5 % | 1.9 % |
| [[25](#_ENREF_25)] | 47 | 40.9 | 19.2 | 47 | 33.0 | 6.9 | 0.54 | 0.13; 0.96 | 2.2 % | 1.9 % |
| [[26](#_ENREF_26)] | 46 | 49.0 | 15.6 | 112 | 36.3 | 13.6 | 0.89 | 0.53; 1.25 | 2.9 % | 1.9 % |
| [[26](#_ENREF_26)] | 46 | 40.0 | 13.4 | 112 | 36.3 | 13.6 | 0.27 | -0.07; 0.62 | 3.1 % | 2.0 % |
| [[26](#_ENREF_26)] | 20 | 55.8 | 14.2 | 112 | 36.3 | 13.6 | 1.41 | 0.91; 1.92 | 1.5 % | 1.7 % |
| [[27](#_ENREF_27)] | 23 | 120.8 | 25.4 | 46 | 78.5 | 46.1 | 1.03 | 0.50; 1.56 | 1.3 % | 1.7 % |
| [[28](#_ENREF_28)] | 9 | 20.6 | 6.6 | 34 | 15.3 | 7.0 | 0.75 | 0.00; 1.50 | 0.7 % | 1.4 % |
| [[28](#_ENREF_28)] | 25 | 21.5 | 5.0 | 34 | 15.3 | 7.0 | 0.98 | 0.43; 1.53 | 1.2 % | 1.7 % |
| [[29](#_ENREF_29)] | 60 | 106.5 | 34.1 | 114 | 50.7 | 35.2 | 1.59 | 1.24; 1.95 | 2.9 % | 1.9 % |
| [[29](#_ENREF_29)] | 24 | 65.6 | 34.8 | 44 | 49.9 | 33.2 | 0.46 | -0.04; 0.96 | 1.5 % | 1.7 % |
| [[30](#_ENREF_30)] | 31 | 22.3 | 13.4 | 31 | 28.8 | 11.7 | -0.51 | -1.02; 0.00 | 1.4 % | 1.7 % |
| [[30](#_ENREF_30)] | 24 | 36.6 | 14.2 | 24 | 31.2 | 10.8 | 0.42 | -0.15; 0.99 | 1.1 % | 1.6 % |
| [[30](#_ENREF_30)] | 11 | 32.4 | 9.0 | 11 | 31.7 | 12.3 | 0.06 | -0.77; 0.90 | 0.5 % | 1.2 % |
| [[30](#_ENREF_30)] | 9 | 23.0 | 13.8 | 9 | 32.5 | 11.1 | -0.72 | -1.68; 0.24 | 0.4 % | 1.1 % |
| [[31](#_ENREF_31)] | 16 | 44.2 | 16.0 | 11 | 41.6 | 21.9 | 0.14 | -0.63; 0.90 | 0.6 % | 1.3 % |
| [[31](#_ENREF_31)] | 35 | 34.6 | 20.1 | 57 | 32.0 | 13.6 | 0.16 | -0.26; 0.58 | 2.1 % | 1.8 % |
| [[32](#_ENREF_32)] | 27 | 51.6 | 16.5 | 25 | 28.7 | 8.0 | 1.72 | 1.08; 2.36 | 0.9 % | 1.5 % |
| [[32](#_ENREF_32)] | 6 | 46.8 | 17.2 | 50 | 22.0 | 9.0 | 2.44 | 1.48; 3.41 | 0.4 % | 1.1 % |
| **Fixed effect** | | |  |  |  |  | **0.65** | **0.59; 0.71** | **100 %** | **-** |
| **Random effects** | | |  |  |  |  | **0.71** | **0.57; 0.86** | **-** | **100 %** |
| **Prediction range** | | |  |  |  |  | - | **-0.27; 1.70** |  |  |

# References

1. Arnett EB, Hayes JP. Use of conifer snags as roosts by female bats in western Oregon. Journal of Wildlife Management. 2009;73(2):214-25. doi: 10.2193/2007-532.

2. Baker MD, Lacki MJ. Day-roosting habitat of female long-legged myotis in ponderosa pine forests. Journal of Wildlife Management. 2006;70(1):207-15. doi: 10.2307/3803562.

3. Brigham RM, Vonhof MJ, Barclay RMR, Gwilliam JC. Roosting behavior and roost-site preferences of forest-dwelling California bats (*Myotis californicus*). Journal of Mammalogy. 1997;78(4):1231-9. doi: 10.2307/1383066.

4. Broders HG, Forbes GJ. Interspecific and intersexual variation in roost-site selection of northern long-eared and little brown bats in the Greater Fundy National Park ecosystem. Journal of Wildlife Management. 2004;68(3):602-10. doi: 10.2193/0022-541x(2004)068[0602:iaivir]2.0.co;2.

5. Callahan EV, Drobney RD, Clawson RL. Selection of summer roosting sites by Indiana bats (*Myotis sodalis*) in Missouri. Journal of Mammalogy. 1997;78(3):818-25. doi: 10.2307/1382939.

6. Carter TC. Summer habitat use of roost trees by the endangered Indiana bat *(Myotis sodalis*) in the Shawnee National Forest of southern Illinois. Southern Illinois: Carbondale University; 2003.

7. Clement MJ, Castleberry SB. Southeastern myotis (*Myotis austroriparius*) roost selection in cypress-gum swamps. Acta Chiropterologica. 2013;15(1):133-41. doi: 10.3161/150811013x667939.

8. Cryan PM, Bogan MA, Yanega GM. Roosting habits of four species in the Black Hills of South Dakota. Acta Chiropterologica. 2001;3:43-52.

9. Fabianek F, Simard MA, Racine B. E, Desrochers A. Selection of roosting habitat by male *Myotis* bats in a boreal forest. Canadian Journal of Zoology. 2015;(0):539-46. doi: 10.1139/cjz-2014-0294.

10. Fleming HL, Jones JC, Belant JL, Richardson DM. Multi-scale roost site selection by Rafinesque's big-eared bat (*Corynorhinus rafinesquii*) and southeastern myotis (*Myotis austroriparius*) in Mississippi. American Midland Naturalist. 2013;169(1):43-55. doi: 10.1674/0003-0031-169.1.43.

11. Foster RW, Kurta A. Roosting ecology of the northern bat (*Myotis septentrionalis*) and comparisons with the endangered Indiana bat (*Myotis sodalis*). Journal of Mammalogy. 1999;80(2):659-72. doi: 10.2307/1383310.

12. Grindal SD. Habitat use by bats, *Myotis* spp., in western Newfoundland. Canadian Field-Naturalist. 1999;113:258-63.

13. Herder MJ, Jackson JG. Roost preferences of long-legged myotis in northern Arizona. Transactions of the Western Section of the Wildlife Society. 2000;36:1-7.

14. Johnson JB, Ford WM, Rodrigue JL, Edwards JW, Johnson CM. Roost selection by male Indiana myotis following forest fires in Central Appalachian hardwood forests. Journal of Fish and Wildlife Management. 2010;1(2):111-21. doi: 10.3996/042010-JFWM-007.

15. Jung TS, Thompson ID, Titman RD. Roost site selection by forest-dwelling male *Myotis* in central Ontario, Canada. Forest Ecology and Management. 2004;202(1-3):325-35. doi: 10.1016/j.foreco.2004.07.043.

16. Kniowski AB, Gehrt SD. Summer ecology of Indiana bats in Ohio. Columbus, OH, USA: 2011.

17. Lacki MJ, Baker MD. Day roosts of female fringed myotis (*Myotis thysanodes*) in xeric forests of the Pacific Northwest. Journal of Mammalogy. 2007;88(4):967-73. doi: 10.1644/06-MAMM-A-255R.1.

18. Menzel MA, Owen SF, Ford WM, Edwards JW, Wood PB, Chapman BR, et al. Roost tree selection by northern long-eared bat (*Myotis septentrionalis*) maternity colonies in an industrial forest of the central Appalachian mountains. Forest Ecology and Management. 2002;155(1):107-14. doi: 10.1016/S0378-1127(01)00551-5.

19. Miles AC, Castleberry SB, Miller DA, Conner LM. Multi-scale roost-site selection by evening bats on pine-dominated landscapes in southwest Georgia. Journal of Wildlife Management. 2006;70(5):1191-9. doi: 10.2193/0022-541x(2006)70[1191:mrsbeb]2.0.co;2.

20. Ormsbee PC, McComb WC. Selection of day roosts by female long-legged myotis in the central Oregon Cascade range. Journal of Wildlife Management. 1998;62(2):596-603. doi: 10.2307/3802335.

21. Parsons S, Lewis KJ, Psyllakis JM. Relationships between roosting habitat of bats and decay of aspen in the sub-boreal forests of British Columbia. Forest Ecology and Management. 2003;177(1–3):559-70. doi: 10.1016/S0378-1127(02)00448-6.

22. Perry RW, Thill RE. Roost selection by male and female northern long-eared bats in a pine-dominated landscape. Forest Ecology and Management. 2007;247(1-3):220-6. doi: 10.1016/j.foreco.2007.04.041.

23. Psyllakis JM, Brigham RM. Characteristics of diurnal roosts used by female *Myotis* bats in sub-boreal forests. Forest Ecology and Management. 2006;223(1-3):93-102. doi: 10.1016/j.foreco.2005.03.071.

24. Rabe MJ, Morrell TE, Green H, Devos JJC, Miller CR. Characteristics of ponderosa pine snag roosts used by reproductive bats in northern Arizona. Journal of Wildlife Management. 1998;62:612-21. doi: 10.2307/3802337.

25. Sasse DB, Pekins PJ. Summer roosting ecology of northern long-eared bats (*Myotis septentrionalis*) in the White Mountain National Forest. In: Barclay RMR, Brigham RM, editors. Bats and Forests Symposium; October 19-21, 1995; Organized by the British Columbia Ministry of Forests. Victoria, BC1996. p. 91-101.

26. Vonhof MJ, Gwilliam JC. Intra- and interspecific patterns of day roost selection by three species of forest-dwelling bats in southern British Columbia. Forest Ecology and Management. 2007;252(1-3):165-75. doi: 10.1016/j.foreco.2007.06.046.

27. Weller TJ, Zabel CJ. Characteristics of fringed myotis day roosts in northern California. Journal of Wildlife Management. 2001;65(3):489-97. doi: 10.2307/3803102.

28. Perry RW, Thill RE. Roost selection by big brown bats in forests of Arkansas: importance of pine snags and open forest habitats to males. Southeastern Naturalist 2008;7(4):607-18. doi: 10.1656/1528-7092-7.4.607.

29. Boland JL, Hayes JP, Smith WP, Huso MM. Selection of day-roosts by Keen's myotis (*Myotis keenii*) at multiple spatial scales. Journal of Mammalogy. 2009; 90(1):222-34. doi: 10.1644/07-MAMM-A-369.1.

30. Hein CD. Bat activity and roost-site selection on an intensively managed pine landscape with forested corridors in the lower coastal plain of South Carolina. Athens, GA, USA: The University of Georgia; 2009.

31. Lacki MJ, Cox DR, Dodd LE, Dickinson MB. Response of Northern bats (*Myotis septentrionalis*) to prescribed fires in eastern Kentucky forests. Journal of Mammalogy. 2009;90(5):1165-75. doi: 10.1644/08-MAMM-A-349.1.

32. Barclay RMR, Faure PA, Farr DR. Roosting behavior and roost selection by migrating silver-haired bats (*Lasionycteris noctivagans*). Journal of Mammalogy. 1988;69(4):821-5. doi: 10.2307/1381639.
