# Supplementary material for: Exploring Regional Variation in Roost Selection by Bats: Evidence from a Meta-Analysis
Source: PLoS One. 2015 Sep 29;10(9):e0139126. doi: 10.1371/journal.pone.0139126 (PMC4587962; doi:10.1371/journal.pone.0139126)
Supplement: S2 Table — Number of selected and random trees is provided for each dataset with corresponding mean, standard deviation (SD), standardized mean difference (SMD) with 95% CI, fixed weight (W), and random weight. Fixed effect and random effects SMD with 95% CI, and prediction intervals are provided at the end of the table. All values are rounded upward to two decimal places. (DOCX) [file pone.0139126.s002.docx]

# Supporting information 2

## S2 Table. Meta-analysis on tree height (m). Number of selected and random trees is provided for each dataset with corresponding mean, standard deviation (SD), standardized mean difference (SMD) with 95 % CI, fixed weight (W), and random weight. Fixed effect and random effects SMD with 95 % CI, and prediction intervals are provided at the end of the table. All values are rounded upward to two decimal places.

|  | **Selected trees** | | | **Random trees** | | |  |  |  |  |
| --- | --- | --- | --- | --- | --- | --- | --- | --- | --- | --- |
| **Study** | ***N*** | **Mean** | **SD** | ***N*** | **Mean** | **SD** | **SMD** | **95 % CI** | **W(fixed)** | **W(random)** |
| [[1](#_ENREF_1)] | 105 | 22.8 | 9.2 | 119 | 23.1 | 13.1 | -0.03 | -0.29; 0.24 | 6.5 % | 2.6 % |
| [[1](#_ENREF_1)] | 24 | 17.7 | 10.8 | 23 | 21.9 | 12.5 | -0.35 | -0.93; 0.22 | 1.3 % | 2.1 % |
| [[1](#_ENREF_1)] | 42 | 24.3 | 9.1 | 104 | 21.9 | 13.3 | 0.20 | -0.16; 0.55 | 3.5 % | 2.4 % |
| [[1](#_ENREF_1)] | 35 | 34.9 | 8.9 | 33 | 32.3 | 11.5 | 0.25 | -0.23; 0.73 | 2.0 % | 2.3 % |
| [[1](#_ENREF_1)] | 22 | 34.3 | 11.3 | 26 | 36.8 | 17.3 | -0.17 | -0.73; 0.40 | 1.4 % | 2.1 % |
| [[2](#_ENREF_2)] | 164 | 26.7 | 7.7 | 160 | 14.7 | 8.9 | 1.45 | 1.20; 1.69 | 7.4 % | 2.6 % |
| [[2](#_ENREF_2)] | 28 | 31.5 | 12.2 | 160 | 14.7 | 8.9 | 1.78 | 1.34; 2.22 | 2.3 % | 2.3 % |
| [[3](#_ENREF_3)] | 19 | 27.0 | 7.9 | 38 | 17.0 | 8.9 | 1.15 | 0.56; 1.74 | 1.3 % | 2.1 % |
| [[4](#_ENREF_4)] | 55 | 13.5 | 3.7 | 55 | 12.4 | 5.2 | 0.24 | -0.13; 0.62 | 3.2 % | 2.4 % |
| [[4](#_ENREF_4)] | 57 | 11.5 | 3.8 | 57 | 10.7 | 4.5 | 0.19 | -0.18; 0.56 | 3.3 % | 2.4 % |
| [[4](#_ENREF_4)] | 48 | 12.2 | 4.9 | 48 | 11.8 | 4.9 | 0.08 | -0.32; 0.48 | 2.8 % | 2.4 % |
| [[5](#_ENREF_5)] | 47 | 17.5 | 6.5 | 37 | 15.7 | 6.1 | 0.29 | -0.14; 0.73 | 2.4 % | 2.3 % |
| [[5](#_ENREF_5)] | 19 | 15.8 | 8.5 | 46 | 14.9 | 5.8 | 0.13 | -0.41; 0.66 | 1.6 % | 2.2 % |
| [[6](#_ENREF_6)] | 25 | 24.8 | 4.3 | 314 | 21.4 | 7.0 | 0.50 | 0.09; 0.90 | 2.7 % | 2.4 % |
| [[7](#_ENREF_7)] | 21 | 9.1 | 5.9 | 30 | 11.4 | 7.1 | -0.34 | -0.90; 0.22 | 1.4 % | 2.1 % |
| [[7](#_ENREF_7)] | 8 | 22.2 | 2.5 | 30 | 11.4 | 7.1 | 1.63 | 0.76; 2.51 | 0.6 % | 1.6 % |
| [[7](#_ENREF_7)] | 9 | 6.9 | 6.3 | 30 | 11.4 | 7.1 | -0.64 | -1.40; 0.12 | 0.8 % | 1.8 % |
| [[7](#_ENREF_7)] | 7 | 11.0 | 7.1 | 30 | 11.4 | 7.1 | -0.06 | -0.88; 0.77 | 0.7 % | 1.7 % |
| [[8](#_ENREF_8)] | 8 | 15.8 | 3.0 | 8 | 14.8 | 3.0 | 0.30 | -0.68; 1.29 | 0.5 % | 1.5 % |
| [[8](#_ENREF_8)] | 40 | 9.6 | 3.1 | 40 | 6.6 | 3.0 | 0.95 | 0.48; 1.41 | 2.1 % | 2.3 % |
| [[9](#_ENREF_9)] | 52 | 21.1 | 7.2 | 61 | 16.1 | 9.4 | 0.59 | 0.21; 0.97 | 3.1 % | 2.4 % |
| [[10](#_ENREF_10)] | 16 | 21.7 | 4.4 | 6 | 12.9 | 5.3 | 1.80 | 0.69; 2.90 | 0.4 % | 1.3 % |
| [[11](#_ENREF_11)] | 15 | 20.1 | 4.3 | 52 | 15.5 | 3.6 | 1.21 | 0.60; 1.82 | 1.2 % | 2.1 % |
| [[11](#_ENREF_11)] | 11 | 21.0 | 7.0 | 52 | 15.5 | 3.6 | 1.25 | 0.56; 1.94 | 0.9 % | 1.9 % |
| [[12](#_ENREF_12)] | 6 | 31.3 | 9.3 | 50 | 14.4 | 9.1 | 1.82 | 0.91; 2.74 | 0.5 % | 1.6 % |
| [[13](#_ENREF_13)] | 134 | 28.3 | 7.4 | 100 | 15.3 | 8.9 | 1.60 | 1.31; 1.90 | 5.0 % | 2.5 % |
| [[14](#_ENREF_14)] | 12 | 18.7 | 5.2 | 12 | 16.8 | 7.3 | 0.29 | -0.51; 1.10 | 0.7 % | 1.7 % |
| [[15](#_ENREF_15)] | 111 | 22.2 | 5.3 | 111 | 19.5 | 6.3 | 0.46 | 0.20; 0.73 | 6.3 % | 2.6 % |
| [[15](#_ENREF_15)] | 57 | 15.4 | 6.8 | 57 | 13.6 | 3.8 | 0.33 | -0.04; 0.70 | 3.3 % | 2.4 % |
| [[16](#_ENREF_16)] | 19 | 22.6 | 8.2 | 25 | 18.8 | 9.2 | 0.42 | -0.18; 1.02 | 1.2 % | 2.1 % |
| [[16](#_ENREF_16)] | 17 | 16.4 | 9.1 | 21 | 20.2 | 8.5 | -0.43 | -1.08; 0.22 | 1.1 % | 2.0 % |
| [[17](#_ENREF_17)] | 43 | 18.8 | 6.6 | 58 | 17.7 | 8.4 | 0.14 | -0.25; 0.54 | 2.9 % | 2.4 % |
| [[17](#_ENREF_17)] | 54 | 17.8 | 8.1 | 54 | 15.9 | 8.8 | 0.22 | -0.16; 0.60 | 3.1 % | 2.4 % |
| [[18](#_ENREF_18)] | 47 | 14.8 | 6.9 | 47 | 8.8 | 2.7 | 1.14 | 0.70; 1.58 | 2.3 % | 2.3 % |
| [[19](#_ENREF_19)] | 46 | 23.7 | 8.4 | 112 | 18.6 | 10.0 | 0.53 | 0.18; 0.88 | 3.7 % | 2.5 % |
| [[19](#_ENREF_19)] | 46 | 29.4 | 9.0 | 112 | 18.6 | 10.0 | 1.10 | 0.74; 1.47 | 3.4 % | 2.4 % |
| [[19](#_ENREF_19)] | 20 | 29.3 | 6.7 | 112 | 18.6 | 10.0 | 1.11 | 0.61; 1.60 | 1.8 % | 2.2 % |
| [[20](#_ENREF_20)] | 9 | 12.3 | 4.2 | 34 | 6.5 | 5.3 | 1.12 | 0.35; 1.90 | 0.7 % | 1.8 % |
| [[20](#_ENREF_20)] | 25 | 12.0 | 4.5 | 34 | 6.5 | 5.3 | 1.10 | 0.54; 1.65 | 1.4 % | 2.1 % |
| [[21](#_ENREF_21)] | 31 | 16.6 | 7.2 | 31 | 19.3 | 6.7 | -0.38 | -0.89; 0.12 | 1.8 % | 2.2 % |
| [[21](#_ENREF_21)] | 24 | 24.9 | 4.9 | 24 | 22.4 | 6.9 | 0.41 | -0.16; 0.98 | 1.4 % | 2.1 % |
| [[21](#_ENREF_21)] | 11 | 22.3 | 5.0 | 11 | 20.3 | 8.0 | 0.29 | -0.55; 1.13 | 0.6 % | 1.7 % |
| [[21](#_ENREF_21)] | 9 | 16.9 | 9.6 | 9 | 20.2 | 8.1 | -0.35 | -1.29; 0.58 | 0.5 % | 1.6 % |
| [[22](#_ENREF_22)] | 16 | 20.6 | 10.0 | 11 | 12.1 | 7.0 | 0.93 | 0.11; 1.74 | 0.7 % | 1.7 % |
| [[22](#_ENREF_22)] | 35 | 19.7 | 8.3 | 57 | 17.8 | 7.6 | 0.24 | -0.18; 0.66 | 2.5 % | 2.4 % |
| [[23](#_ENREF_23)] | 27 | 14.8 | 8.1 | 25 | 15.3 | 3.0 | -0.07 | -0.61; 0.48 | 1.5 % | 2.2 % |
| [[23](#_ENREF_23)] | 6 | 12.9 | 5.0 | 50 | 12.5 | 3.1 | 0.12 | -0.73; 0.96 | 0.6 % | 1.7 % |
| **Fixed effect** | | |  |  |  |  | **0.56** | **0.49; 0.62** | **100 %** | **-** |
| **Random effects** | | |  |  |  |  | **0.51** | **0.34; 0.69** | **-** | **100 %** |
| **Prediction range** | | |  |  |  |  | - | **-0.61; 1.64** |  |  |

# References

1. Arnett EB, Hayes JP. Use of conifer snags as roosts by female bats in western Oregon. Journal of Wildlife Management. 2009;73(2):214-25. doi: 10.2193/2007-532.

2. Baker MD, Lacki MJ. Day-roosting habitat of female long-legged myotis in ponderosa pine forests. Journal of Wildlife Management. 2006;70(1):207-15. doi: 10.2307/3803562.

3. Brigham RM, Vonhof MJ, Barclay RMR, Gwilliam JC. Roosting behavior and roost-site preferences of forest-dwelling California bats (*Myotis californicus*). Journal of Mammalogy. 1997;78(4):1231-9. doi: 10.2307/1383066.

4. Broders HG, Forbes GJ. Interspecific and intersexual variation in roost-site selection of northern long-eared and little brown bats in the Greater Fundy National Park ecosystem. Journal of Wildlife Management. 2004;68(3):602-10. doi: 10.2193/0022-541x(2004)068[0602:iaivir]2.0.co;2.

5. Carter TC. Summer habitat use of roost trees by the endangered Indiana bat *(Myotis sodalis*) in the Shawnee National Forest of southern Illinois. Southern Illinois: Carbondale University; 2003.

6. Clement MJ, Castleberry SB. Southeastern myotis (*Myotis austroriparius*) roost selection in cypress-gum swamps. Acta Chiropterologica. 2013;15(1):133-41. doi: 10.3161/150811013x667939.

7. Cryan PM, Bogan MA, Yanega GM. Roosting habits of four species in the Black Hills of South Dakota. Acta Chiropterologica. 2001;3:43-52.

8. Fabianek F, Simard MA, Racine B. E, Desrochers A. Selection of roosting habitat by male *Myotis* bats in a boreal forest. Canadian Journal of Zoology. 2015;(0):539-46. doi: 10.1139/cjz-2014-0294.

9. Herder MJ, Jackson JG. Roost preferences of long-legged myotis in northern Arizona. Transactions of the Western Section of the Wildlife Society. 2000;36:1-7.

10. Johnson JB, Ford WM, Rodrigue JL, Edwards JW, Johnson CM. Roost selection by male Indiana myotis following forest fires in Central Appalachian hardwood forests. Journal of Fish and Wildlife Management. 2010;1(2):111-21. doi: 10.3996/042010-JFWM-007.

11. Jung TS, Thompson ID, Titman RD. Roost site selection by forest-dwelling male *Myotis* in central Ontario, Canada. Forest Ecology and Management. 2004;202(1-3):325-35. doi: 10.1016/j.foreco.2004.07.043.

12. Lacki MJ, Baker MD. Day roosts of female fringed myotis (*Myotis thysanodes*) in xeric forests of the Pacific Northwest. Journal of Mammalogy. 2007;88(4):967-73. doi: 10.1644/06-MAMM-A-255R.1.

13. Lacki MJ, Baker MD, Johnson JS. Geographic variation in roost-site selection of long-legged myotis in the Pacific Northwest. Journal of Wildlife Management. 2010;74(6):1218-28. doi: 10.2307/40801116.

14. Menzel MA, Owen SF, Ford WM, Edwards JW, Wood PB, Chapman BR, et al. Roost tree selection by northern long-eared bat (*Myotis septentrionalis*) maternity colonies in an industrial forest of the central Appalachian mountains. Forest Ecology and Management. 2002;155(1):107-14. doi: 10.1016/S0378-1127(01)00551-5.

15. Miles AC, Castleberry SB, Miller DA, Conner LM. Multi-scale roost-site selection by evening bats on pine-dominated landscapes in southwest Georgia. Journal of Wildlife Management. 2006;70(5):1191-9. doi: 10.2193/0022-541x(2006)70[1191:mrsbeb]2.0.co;2.

16. Psyllakis JM, Brigham RM. Characteristics of diurnal roosts used by female *Myotis* bats in sub-boreal forests. Forest Ecology and Management. 2006;223(1-3):93-102. doi: 10.1016/j.foreco.2005.03.071.

17. Rabe MJ, Morrell TE, Green H, Devos JJC, Miller CR. Characteristics of ponderosa pine snag roosts used by reproductive bats in northern Arizona. Journal of Wildlife Management. 1998;62:612-21. doi: 10.2307/3802337.

18. Sasse DB, Pekins PJ. Summer roosting ecology of northern long-eared bats (*Myotis septentrionalis*) in the White Mountain National Forest. In: Barclay RMR, Brigham RM, editors. Bats and Forests Symposium; October 19-21, 1995; Organized by the British Columbia Ministry of Forests. Victoria, BC1996. p. 91-101.

19. Vonhof MJ, Gwilliam JC. Intra- and interspecific patterns of day roost selection by three species of forest-dwelling bats in southern British Columbia. Forest Ecology and Management. 2007;252(1-3):165-75. doi: 10.1016/j.foreco.2007.06.046.

20. Perry RW, Thill RE. Roost selection by big brown bats in forests of Arkansas: importance of pine snags and open forest habitats to males. Southeastern Naturalist 2008;7(4):607-18. doi: 10.1656/1528-7092-7.4.607.

21. Hein CD. Bat activity and roost-site selection on an intensively managed pine landscape with forested corridors in the lower coastal plain of South Carolina. Athens, GA, USA: The University of Georgia; 2009.

22. Lacki MJ, Cox DR, Dodd LE, Dickinson MB. Response of Northern bats (*Myotis septentrionalis*) to prescribed fires in eastern Kentucky forests. Journal of Mammalogy. 2009;90(5):1165-75. doi: 10.1644/08-MAMM-A-349.1.

23. Barclay RMR, Faure PA, Farr DR. Roosting behavior and roost selection by migrating silver-haired bats (*Lasionycteris noctivagans*). Journal of Mammalogy. 1988;69(4):821-5. doi: 10.2307/1381639.
