# Supplementary material for: Exploring Regional Variation in Roost Selection by Bats: Evidence from a Meta-Analysis
Source: PLoS One. 2015 Sep 29;10(9):e0139126. doi: 10.1371/journal.pone.0139126 (PMC4587962; doi:10.1371/journal.pone.0139126)
Supplement: S3 Table — Number of selected and random trees is provided for each dataset with corresponding mean, standard deviation (SD), standardized mean difference (SMD) with 95% CI, fixed weight (W), and random weight. Fixed effect and random effects SMD with 95% CI, and prediction intervals are provided at the end of the table. All values are rounded upward to two decimal places. (DOCX) [file pone.0139126.s003.docx]

# Supporting information 3

## S3 Table. Meta-analysis on snag density (stems/0.1 ha). Number of selected and random trees is provided for each dataset with corresponding mean, standard deviation (SD), standardized mean difference (SMD) with 95 % CI, fixed weight (W), and random weight. Fixed effect and random effects SMD with 95 % CI, and prediction intervals are provided at the end of the table. All values are rounded upward to two decimal places.

|  | **Selected trees** | | | **Random trees** | | |  |  |  |  |
| --- | --- | --- | --- | --- | --- | --- | --- | --- | --- | --- |
| **Study** | ***N*** | **Mean** | **SD** | ***N*** | **Mean** | **SD** | **SMD** | **95 % CI** | **W(fixed)** | **W(random)** |
| [[1](#_ENREF_1)] | 105 | 2.3 | 3.1 | 119 | 0.5 | 1.1 | 0.80 | 0.53; 1.07 | 7.7 % | 4.0 % |
| [[1](#_ENREF_1)] | 24 | 1.5 | 2.5 | 23 | 0.6 | 1.0 | 0.47 | -0.11; 1.05 | 1.7 % | 2.7 % |
| [[1](#_ENREF_1)] | 42 | 2.3 | 1.9 | 104 | 0.7 | 2.0 | 0.79 | 0.42; 1.16 | 4.2 % | 3.6 % |
| [[1](#_ENREF_1)] | 35 | 1.8 | 1.8 | 33 | 1.0 | 1.7 | 0.45 | -0.03; 0.93 | 2.5 % | 3.1 % |
| [[1](#_ENREF_1)] | 22 | 1.4 | 1.9 | 26 | 0.9 | 1.5 | 0.29 | -0.28; 0.86 | 1.8 % | 2.7 % |
| [[2](#_ENREF_2)] | 164 | 42.7 | 46.1 | 160 | 16.6 | 22.8 | 0.71 | 0.49; 0.94 | 11.3 % | 4.2 % |
| [[2](#_ENREF_2)] | 28 | 41.2 | 84.1 | 160 | 16.6 | 22.8 | 0.64 | 0.23; 1.05 | 3.4 % | 3.4 % |
| [[3](#_ENREF_3)] | 19 | 55.0 | 37.0 | 38 | 50.0 | 3.0 | 0.23 | -0.32; 0.78 | 1.9 % | 2.8 % |
| [[4](#_ENREF_4)] | 55 | 7.0 | 5.2 | 55 | 6.9 | 5.9 | 0.02 | -0.36; 0.39 | 4.1 % | 3.6 % |
| [[4](#_ENREF_4)] | 57 | 11.0 | 7.6 | 57 | 9.3 | 6.0 | 0.25 | -0.12; 0.62 | 4.2 % | 3.6 % |
| [[4](#_ENREF_4)] | 48 | 12.7 | 6.9 | 48 | 9.0 | 4.9 | 0.61 | 0.20; 1.02 | 3.4 % | 3.4 % |
| [[5](#_ENREF_5)] | 21 | 0.3 | 0.6 | 30 | 0.1 | 0.4 | 0.40 | -0.16; 0.96 | 1.8 % | 2.7 % |
| [[5](#_ENREF_5)] | 8 | 0.6 | 0.7 | 30 | 0.1 | 0.4 | 1.03 | 0.22; 1.85 | 0.9 % | 1.9 % |
| [[5](#_ENREF_5)] | 9 | 0.5 | 0.8 | 30 | 0.1 | 0.4 | 0.76 | 0.00; 1.53 | 1.0 % | 2.0 % |
| [[5](#_ENREF_5)] | 7 | 0.9 | 1.0 | 30 | 0.1 | 0.4 | 1.42 | 0.53; 2.31 | 0.7 % | 1.7 % |
| [[6](#_ENREF_6)] | 8 | 1.0 | 1.8 | 8 | 1.0 | 1.1 | 0.00 | -0.98; 0.98 | 0.6 % | 1.5 % |
| [[6](#_ENREF_6)] | 40 | 6.4 | 4.4 | 40 | 1.9 | 2.6 | 1.23 | 0.75; 1.71 | 2.5 % | 3.1 % |
| [[7](#_ENREF_7)] | 52 | 6.4 | 9.1 | 61 | 6.8 | 7.7 | -0.05 | -0.42; 0.32 | 4.2 % | 3.6 % |
| [[8](#_ENREF_8)] | 15 | 32.7 | 22.5 | 52 | 33.7 | 17.3 | -0.05 | -0.63; 0.52 | 1.7 % | 2.7 % |
| [[8](#_ENREF_8)] | 11 | 25.5 | 14.3 | 52 | 33.7 | 17.3 | -0.48 | -1.14; 0.18 | 1.3 % | 2.4 % |
| [[9](#_ENREF_9)] | 6 | 17.2 | 9.3 | 50 | 8.8 | 6.8 | 1.17 | 0.30; 2.05 | 0.7 % | 1.7 % |
| [[10](#_ENREF_10)] | 12 | 1.4 | 0.9 | 12 | 1.1 | 1.0 | 0.24 | -0.56; 1.04 | 0.9 % | 1.9 % |
| [[11](#_ENREF_11)] | 17 | 56.7 | 38.2 | 21 | 34.8 | 20.9 | 0.72 | 0.06; 1.38 | 1.3 % | 2.3 % |
| [[12](#_ENREF_12)] | 43 | 6.5 | 6.6 | 58 | 1.0 | 3.1 | 1.12 | 0.70; 1.55 | 3.2 % | 3.3 % |
| [[12](#_ENREF_12)] | 54 | 10.6 | 10.3 | 54 | 2.0 | 4.4 | 1.08 | 0.67; 1.48 | 3.5 % | 3.4 % |
| [[13](#_ENREF_13)] | 47 | 3.9 | 2.1 | 47 | 2.3 | 2.1 | 0.77 | 0.35; 1.19 | 3.2 % | 3.4 % |
| [[14](#_ENREF_14)] | 46 | 7.0 | 7.4 | 112 | 6.0 | 5.0 | 0.17 | -0.17; 0.52 | 4.8 % | 3.7 % |
| [[14](#_ENREF_14)] | 46 | 8.0 | 5.6 | 112 | 6.0 | 5.0 | 0.38 | 0.04; 0.73 | 4.8 % | 3.7 % |
| [[14](#_ENREF_14)] | 20 | 7.0 | 3.7 | 112 | 6.0 | 5.0 | 0.21 | -0.27; 0.68 | 2.5 % | 3.1 % |
| [[15](#_ENREF_15)] | 23 | 10.8 | 4.3 | 46 | 11.3 | 14.9 | -0.04 | -0.54; 0.46 | 2.3 % | 3.0 % |
| [[16](#_ENREF_16)] | 60 | 0.4 | 0.2 | 114 | 0.4 | 0.1 | 0.16 | -0.15; 0.47 | 5.8 % | 3.8 % |
| [[16](#_ENREF_16)] | 24 | 0.5 | 0.2 | 44 | 0.4 | 0.1 | 0.79 | 0.28; 1.31 | 2.1 % | 2.9 % |
| [[17](#_ENREF_17)] | 16 | 4.1 | 2.7 | 11 | 2.8 | 2.9 | 0.46 | -0.32; 1.23 | 0.9 % | 2.0 % |
| [[17](#_ENREF_17)] | 35 | 2.5 | 1.8 | 57 | 3.3 | 3.9 | -0.23 | -0.65; 0.19 | 3.2 % | 3.3 % |
| **Fixed effect** | | |  |  |  |  | **0.48** | **0.41; 0.56** | **100 %** | **-** |
| **Random effects** | | |  |  |  |  | **0.47** | **0.33; 0.62** | **-** | **100 %** |
| **Prediction range** | | |  |  |  |  | **-** | **-0.23; 1.17** |  |  |

#####

# References

1. Arnett EB, Hayes JP. Use of conifer snags as roosts by female bats in western Oregon. Journal of Wildlife Management. 2009;73(2):214-25. doi: 10.2193/2007-532.

2. Baker MD, Lacki MJ. Day-roosting habitat of female long-legged myotis in ponderosa pine forests. Journal of Wildlife Management. 2006;70(1):207-15. doi: 10.2307/3803562.

3. Brigham RM, Vonhof MJ, Barclay RMR, Gwilliam JC. Roosting behavior and roost-site preferences of forest-dwelling California bats (*Myotis californicus*). Journal of Mammalogy. 1997;78(4):1231-9. doi: 10.2307/1383066.

4. Broders HG, Forbes GJ. Interspecific and intersexual variation in roost-site selection of northern long-eared and little brown bats in the Greater Fundy National Park ecosystem. Journal of Wildlife Management. 2004;68(3):602-10. doi: 10.2193/0022-541x(2004)068[0602:iaivir]2.0.co;2.

5. Cryan PM, Bogan MA, Yanega GM. Roosting habits of four species in the Black Hills of South Dakota. Acta Chiropterologica. 2001;3:43-52.

6. Fabianek F, Simard MA, Racine B. E, Desrochers A. Selection of roosting habitat by male *Myotis* bats in a boreal forest. Canadian Journal of Zoology. 2015;(0):539-46. doi: 10.1139/cjz-2014-0294.

7. Herder MJ, Jackson JG. Roost preferences of long-legged myotis in northern Arizona. Transactions of the Western Section of the Wildlife Society. 2000;36:1-7.

8. Jung TS, Thompson ID, Titman RD. Roost site selection by forest-dwelling male *Myotis* in central Ontario, Canada. Forest Ecology and Management. 2004;202(1-3):325-35. doi: 10.1016/j.foreco.2004.07.043.

9. Lacki MJ, Baker MD. Day roosts of female fringed myotis (*Myotis thysanodes*) in xeric forests of the Pacific Northwest. Journal of Mammalogy. 2007;88(4):967-73. doi: 10.1644/06-MAMM-A-255R.1.

10. Menzel MA, Owen SF, Ford WM, Edwards JW, Wood PB, Chapman BR, et al. Roost tree selection by northern long-eared bat (*Myotis septentrionalis*) maternity colonies in an industrial forest of the central Appalachian mountains. Forest Ecology and Management. 2002;155(1):107-14. doi: 10.1016/S0378-1127(01)00551-5.

11. Psyllakis JM, Brigham RM. Characteristics of diurnal roosts used by female *Myotis* bats in sub-boreal forests. Forest Ecology and Management. 2006;223(1-3):93-102. doi: 10.1016/j.foreco.2005.03.071.

12. Rabe MJ, Morrell TE, Green H, Devos JJC, Miller CR. Characteristics of ponderosa pine snag roosts used by reproductive bats in northern Arizona. Journal of Wildlife Management. 1998;62:612-21. doi: 10.2307/3802337.

13. Sasse DB, Pekins PJ. Summer roosting ecology of northern long-eared bats (*Myotis septentrionalis*) in the White Mountain National Forest. In: Barclay RMR, Brigham RM, editors. Bats and Forests Symposium; October 19-21, 1995; Organized by the British Columbia Ministry of Forests. Victoria, BC1996. p. 91-101.

14. Vonhof MJ, Gwilliam JC. Intra- and interspecific patterns of day roost selection by three species of forest-dwelling bats in southern British Columbia. Forest Ecology and Management. 2007;252(1-3):165-75. doi: 10.1016/j.foreco.2007.06.046.

15. Weller TJ, Zabel CJ. Characteristics of fringed myotis day roosts in northern California. Journal of Wildlife Management. 2001;65(3):489-97. doi: 10.2307/3803102.

16. Boland JL, Hayes JP, Smith WP, Huso MM. Selection of day-roosts by Keen's myotis (*Myotis keenii*) at multiple spatial scales. Journal of Mammalogy. 2009; 90(1):222-34. doi: 10.1644/07-MAMM-A-369.1.

17. Lacki MJ, Cox DR, Dodd LE, Dickinson MB. Response of Northern bats (*Myotis septentrionalis*) to prescribed fires in eastern Kentucky forests. Journal of Mammalogy. 2009;90(5):1165-75. doi: 10.1644/08-MAMM-A-349.1.
