# Supplementary material for: Exploring Regional Variation in Roost Selection by Bats: Evidence from a Meta-Analysis
Source: PLoS One. 2015 Sep 29;10(9):e0139126. doi: 10.1371/journal.pone.0139126 (PMC4587962; doi:10.1371/journal.pone.0139126)
Supplement: S5 Table — Number of selected and random trees is provided for each dataset with corresponding mean, standard deviation (SD), standardized mean difference (SMD) with 95% CI, fixed weight (W), and random weight. Fixed effect and random effects SMD with 95% CI, and prediction intervals are provided at the end of the table. All values are rounded upward to two decimal places. (DOCX) [file pone.0139126.s005.docx]

# Supporting information 5

## S5 Table. Meta-analysis on canopy closure (%). Number of selected and random trees is provided for each dataset with corresponding mean, standard deviation (SD), standardized mean difference (SMD) with 95 % CI, fixed weight (W), and random weight. Fixed effect and random effects SMD with 95 % CI, and prediction intervals are provided at the end of the table. All values are rounded upward to two decimal places.

|  | **Selected trees** | | | **Random trees** | | |  |  |  |  |
| --- | --- | --- | --- | --- | --- | --- | --- | --- | --- | --- |
| **Study** | ***N*** | **Mean** | **SD** | ***N*** | **Mean** | **SD** | **SMD** | **95 % CI** | **W(fixed)** | **W(random)** |
| [[1](#_ENREF_1)] | 105 | 27.5 | 16.4 | 119 | 43.6 | 24.0 | -0.8 | -1.04; -0.50 | 7.6 % | 3.4 % |
| [[1](#_ENREF_1)] | 24 | 24.8 | 17.6 | 23 | 34.6 | 24.5 | -0.5 | -1.03; 0.13 | 1.7 % | 2.9 % |
| [[1](#_ENREF_1)] | 42 | 30.7 | 16.2 | 104 | 45.4 | 23.5 | -0.7 | -1.04; -0.31 | 4.2 % | 3.3 % |
| [[1](#_ENREF_1)] | 35 | 37.9 | 17.2 | 33 | 35.6 | 21.8 | 0.12 | -0.36; 0.59 | 2.5 % | 3.1 % |
| [[1](#_ENREF_1)] | 22 | 33.9 | 11.3 | 26 | 29.4 | 23.0 | 0.24 | -0.33; 0.81 | 1.7 % | 2.9 % |
| [[2](#_ENREF_2)] | 164 | 31.2 | 24.3 | 160 | 28.5 | 21.5 | 0.12 | -0.10; 0.34 | 11.9 % | 3.5 % |
| [[2](#_ENREF_2)] | 28 | 22.1 | 20.6 | 160 | 28.5 | 21.5 | -0.3 | -0.70; 0.10 | 3.5 % | 3.3 % |
| [[3](#_ENREF_3)] | 55 | 74.2 | 14.1 | 55 | 69.4 | 19.3 | 0.28 | -0.09; 0.66 | 4.0 % | 3.3 % |
| [[3](#_ENREF_3)] | 57 | 67.4 | 16.6 | 57 | 66.4 | 16.6 | 0.06 | -0.31; 0.43 | 4.2 % | 3.3 % |
| [[3](#_ENREF_3)] | 48 | 62.4 | 19.4 | 48 | 54.2 | 21.5 | 0.4 | -0.01; 0.80 | 3.5 % | 3.2 % |
| [[4](#_ENREF_4)] | 47 | 36.3 | 28.1 | 37 | 36.6 | 35.0 | -0 | -0.44; 0.42 | 3.0 % | 3.2 % |
| [[4](#_ENREF_4)] | 19 | 61.3 | 28.3 | 46 | 44.0 | 35.7 | 0.51 | -0.04; 1.05 | 1.9 % | 3.0 % |
| [[5](#_ENREF_5)] | 25 | 85.9 | 12.3 | 314 | 86.0 | 7.6 | -0 | -0.42; 0.39 | 3.4 % | 3.2 % |
| [[6](#_ENREF_6)] | 8 | 54.0 | 65.0 | 8 | 98.0 | 3.0 | -0.9 | -1.95; 0.14 | 0.5 % | 2.0 % |
| [[6](#_ENREF_6)] | 40 | 44.0 | 75.0 | 40 | 46.0 | 74.0 | -0 | -0.46; 0.41 | 2.9 % | 3.2 % |
| [[7](#_ENREF_7)] | 52 | 55.2 | 19.5 | 61 | 64.2 | 17.2 | -0.5 | -0.86; -0.11 | 4.0 % | 3.3 % |
| [[8](#_ENREF_8)] | 16 | 72.0 | 13.9 | 6 | 88.6 | 1.7 | -1.3 | -2.36; -0.29 | 0.5 % | 2.1 % |
| [[8](#_ENREF_8)] | 34 | 85.4 | 18.1 | 15 | 91.5 | 1.7 | -0.4 | -1.01; 0.22 | 1.5 % | 2.9 % |
| [[9](#_ENREF_9)] | 15 | 39.1 | 16.7 | 52 | 70.0 | 11.5 | -2.4 | -3.09; -1.67 | 1.1 % | 2.7 % |
| [[9](#_ENREF_9)] | 11 | 31.6 | 19.6 | 52 | 70.0 | 11.5 | -2.9 | -3.71; -2.04 | 0.8 % | 2.4 % |
| [[10](#_ENREF_10)] | 57 | 91.4 | 1.0 | 31 | 90.5 | 1.4 | 0.78 | 0.33; 1.24 | 2.8 % | 3.2 % |
| [[11](#_ENREF_11)] | 12 | 91.7 | 8.3 | 12 | 83.4 | 29.1 | 0.37 | -0.43; 1.18 | 0.9 % | 2.5 % |
| [[12](#_ENREF_12)] | 19 | 64.9 | 13.8 | 25 | 67.2 | 15.0 | -0.2 | -0.75; 0.44 | 1.6 % | 2.9 % |
| [[12](#_ENREF_12)] | 17 | 65.5 | 26.4 | 21 | 79.4 | 11.0 | -0.7 | -1.36; -0.04 | 1.3 % | 2.8 % |
| [[13](#_ENREF_13)] | 43 | 48.0 | 13.1 | 58 | 53.0 | 22.9 | -0.3 | -0.65; 0.14 | 3.6 % | 3.3 % |
| [[13](#_ENREF_13)] | 54 | 52.0 | 14.7 | 54 | 57.0 | 14.7 | -0.3 | -0.72; 0.04 | 3.9 % | 3.3 % |
| [[14](#_ENREF_14)] | 47 | 83.0 | 9.6 | 47 | 86.0 | 24.0 | -0.2 | -0.57; 0.24 | 3.4 % | 3.2 % |
| [[15](#_ENREF_15)] | 46 | 42.0 | 28.1 | 112 | 50.0 | 27.8 | -0.3 | -0.63; 0.06 | 4.8 % | 3.3 % |
| [[15](#_ENREF_15)] | 46 | 38.0 | 23.2 | 112 | 50.0 | 27.8 | -0.5 | -0.80; -0.10 | 4.7 % | 3.3 % |
| [[15](#_ENREF_15)] | 20 | 35.0 | 26.9 | 112 | 50.0 | 27.8 | -0.5 | -1.02; -0.06 | 2.4 % | 3.1 % |
| [[16](#_ENREF_16)] | 23 | 78.5 | 12.5 | 46 | 89.2 | 7.5 | -1.1 | -1.66; -0.59 | 2.0 % | 3.0 % |
| [[17](#_ENREF_17)] | 16 | 45.0 | 20.0 | 11 | 51.0 | 23.9 | -0.3 | -1.04; 0.50 | 0.9 % | 2.5 % |
| [[17](#_ENREF_17)] | 35 | 65.0 | 26.0 | 57 | 61.0 | 22.7 | 0.17 | -0.26; 0.59 | 3.2 % | 3.2 % |
| **Fixed effect** | | |  |  |  |  | **-0.2** | **-0.29; -0.14** | **100 %** | **-** |
| **Random effects** | | |  |  |  |  | **-0.3** | **-0.54; -0.09** | **-** | **100 %** |
| **Prediction range** | | |  |  |  |  | - | **-1.56; 0.93** |  |  |

##

# References

1. Arnett EB, Hayes JP. Use of conifer snags as roosts by female bats in western Oregon. Journal of Wildlife Management. 2009;73(2):214-25. doi: 10.2193/2007-532.

2. Baker MD, Lacki MJ. Day-roosting habitat of female long-legged myotis in ponderosa pine forests. Journal of Wildlife Management. 2006;70(1):207-15. doi: 10.2307/3803562.

3. Broders HG, Forbes GJ. Interspecific and intersexual variation in roost-site selection of northern long-eared and little brown bats in the Greater Fundy National Park ecosystem. Journal of Wildlife Management. 2004;68(3):602-10. doi: 10.2193/0022-541x(2004)068[0602:iaivir]2.0.co;2.

4. Carter TC. Summer habitat use of roost trees by the endangered Indiana bat *(Myotis sodalis*) in the Shawnee National Forest of southern Illinois. Southern Illinois: Carbondale University; 2003.

5. Clement MJ, Castleberry SB. Southeastern myotis (*Myotis austroriparius*) roost selection in cypress-gum swamps. Acta Chiropterologica. 2013;15(1):133-41. doi: 10.3161/150811013x667939.

6. Fabianek F, Simard MA, Racine B. E, Desrochers A. Selection of roosting habitat by male *Myotis* bats in a boreal forest. Canadian Journal of Zoology. 2015;(0):539-46. doi: 10.1139/cjz-2014-0294.

7. Herder MJ, Jackson JG. Roost preferences of long-legged myotis in northern Arizona. Transactions of the Western Section of the Wildlife Society. 2000;36:1-7.

8. Johnson JB, Ford WM, Rodrigue JL, Edwards JW, Johnson CM. Roost selection by male Indiana myotis following forest fires in Central Appalachian hardwood forests. Journal of Fish and Wildlife Management. 2010;1(2):111-21. doi: 10.3996/042010-JFWM-007.

9. Jung TS, Thompson ID, Titman RD. Roost site selection by forest-dwelling male *Myotis* in central Ontario, Canada. Forest Ecology and Management. 2004;202(1-3):325-35. doi: 10.1016/j.foreco.2004.07.043.

10. Lacki MJ, Schwierjohann JH. Day-roost characteristics of northern bats in mixed mesophytic forest. Journal of Wildlife Management. 2001;65(3):482-8. doi: 10.2307/3803101.

11. Menzel MA, Owen SF, Ford WM, Edwards JW, Wood PB, Chapman BR, et al. Roost tree selection by northern long-eared bat (*Myotis septentrionalis*) maternity colonies in an industrial forest of the central Appalachian mountains. Forest Ecology and Management. 2002;155(1):107-14. doi: 10.1016/S0378-1127(01)00551-5.

12. Psyllakis JM, Brigham RM. Characteristics of diurnal roosts used by female *Myotis* bats in sub-boreal forests. Forest Ecology and Management. 2006;223(1-3):93-102. doi: 10.1016/j.foreco.2005.03.071.

13. Rabe MJ, Morrell TE, Green H, Devos JJC, Miller CR. Characteristics of ponderosa pine snag roosts used by reproductive bats in northern Arizona. Journal of Wildlife Management. 1998;62:612-21. doi: 10.2307/3802337.

14. Sasse DB, Pekins PJ. Summer roosting ecology of northern long-eared bats (*Myotis septentrionalis*) in the White Mountain National Forest. In: Barclay RMR, Brigham RM, editors. Bats and Forests Symposium; October 19-21, 1995; Organized by the British Columbia Ministry of Forests. Victoria, BC1996. p. 91-101.

15. Vonhof MJ, Gwilliam JC. Intra- and interspecific patterns of day roost selection by three species of forest-dwelling bats in southern British Columbia. Forest Ecology and Management. 2007;252(1-3):165-75. doi: 10.1016/j.foreco.2007.06.046.

16. Weller TJ, Zabel CJ. Characteristics of fringed myotis day roosts in northern California. Journal of Wildlife Management. 2001;65(3):489-97. doi: 10.2307/3803102.

17. Lacki MJ, Cox DR, Dodd LE, Dickinson MB. Response of Northern bats (*Myotis septentrionalis*) to prescribed fires in eastern Kentucky forests. Journal of Mammalogy. 2009;90(5):1165-75. doi: 10.1644/08-MAMM-A-349.1.
