# Supplementary material for: Exploring Regional Variation in Roost Selection by Bats: Evidence from a Meta-Analysis
Source: PLoS One. 2015 Sep 29;10(9):e0139126. doi: 10.1371/journal.pone.0139126 (PMC4587962; doi:10.1371/journal.pone.0139126)
Supplement: S6 Table — Number of selected and random trees is provided for each dataset with corresponding mean, standard deviation (SD), standardized mean difference (SMD) with 95% CI, fixed weight (W), and random weight. Fixed effect and random effects SMD with 95% CI, and prediction intervals are provided at the end of the table. All values are rounded upward to two decimal places. (DOCX) [file pone.0139126.s006.docx]

# Supporting information 6

## S6 Table. Meta-analysis on distance to water (m). Number of selected and random trees is provided for each dataset with corresponding mean, standard deviation (SD), standardized mean difference (SMD) with 95 % CI, fixed weight (W), and random weight. Fixed effect and random effects SMD with 95 % CI, and prediction intervals are provided at the end of the table. All values are rounded upward to two decimal places.

|  | **Selected trees** | | | **Random trees** | | |  |  |  |  |
| --- | --- | --- | --- | --- | --- | --- | --- | --- | --- | --- |
| **Study** | ***N*** | **Mean** | **SD** | ***N*** | **Mean** | **SD** | **SMD** | **95 % CI** | **W(fixed)** | **W(random)** |
| [[1](#_ENREF_1)] | 105 | 1407 | 1332 | 119 | 1829 | 1385 | -0.30 | -0.57; -0.05 | 11.50 % | 6.00 % |
| [[1](#_ENREF_1)] | 24 | 1205 | 926 | 23 | 1470 | 1352 | -0.20 | -0.80; 0.35 | 2.40 % | 3.80 % |
| [[1](#_ENREF_1)] | 42 | 967 | 1056 | 104 | 1883 | 1428 | -0.70 | -1.05; -0.32 | 5.90 % | 5.30 % |
| [[1](#_ENREF_1)] | 35 | 1582 | 1935 | 33 | 1551 | 1338 | 0.02 | -0.46; 0.49 | 3.50 % | 4.50 % |
| [[1](#_ENREF_1)] | 22 | 1480 | 1599 | 26 | 1766 | 1127 | -0.20 | -0.78; 0.36 | 2.50 % | 3.90 % |
| [[2](#_ENREF_2)] | 25 | 627 | 469 | 314 | 580 | 400 | 0.11 | -0.29; 0.52 | 4.80 % | 5.00 % |
| [[3](#_ENREF_3)] | 40 | 779 | 799 | 40 | 1342 | 663 | -0.80 | -1.21; -0.30 | 3.90 % | 4.60 % |
| [[4](#_ENREF_4)] | 8 | 197 | 476 | 157 | 256 | 365 | -0.20 | -0.87; 0.55 | 1.60 % | 3.10 % |
| [[4](#_ENREF_4)] | 7 | 177 | 229 | 147 | 244 | 174 | -0.40 | -1.14; 0.38 | 1.40 % | 2.80 % |
| [[5](#_ENREF_5)] | 52 | 1032 | 413 | 61 | 770 | 500 | 0.56 | 0.19; 0.94 | 5.60 % | 5.20 % |
| [[6](#_ENREF_6)] | 15 | 2283 | 871 | 52 | 1458 | 1262 | 0.69 | 0.10; 1.27 | 2.30 % | 3.70 % |
| [[6](#_ENREF_6)] | 11 | 2283 | 746 | 52 | 1458 | 1262 | 0.68 | 0.02; 1.35 | 1.80 % | 3.30 % |
| [[7](#_ENREF_7)] | 57 | 127 | 100 | 31 | 121 | 89.1 | 0.06 | -0.38; 0.50 | 4.20 % | 4.80 % |
| [[8](#_ENREF_8)] | 111 | 333 | 247 | 111 | 429 | 312 | -0.30 | -0.60; -0.07 | 11.40 % | 6.00 % |
| [[8](#_ENREF_8)] | 57 | 164 | 139 | 57 | 192 | 146 | -0.20 | -0.57; 0.17 | 5.90 % | 5.30 % |
| [[9](#_ENREF_9)] | 33 | 923 | 925 | 66 | 1217 | 780 | -0.40 | -0.77; 0.07 | 4.50 % | 4.90 % |
| [[10](#_ENREF_10)] | 17 | 272 | 288 | 21 | 301 | 206 | -0.10 | -0.76; 0.52 | 2.00 % | 3.40 % |
| [[11](#_ENREF_11)] | 43 | 458 | 315 | 58 | 701 | 350 | -0.70 | -1.13; -0.31 | 4.80 % | 5.00 % |
| [[11](#_ENREF_11)] | 54 | 759 | 353 | 54 | 855 | 558 | -0.20 | -0.58; 0.17 | 5.60 % | 5.20 % |
| [[12](#_ENREF_12)] | 23 | 117 | 131 | 46 | 150 | 130 | -0.20 | -0.75; 0.26 | 3.20 % | 4.30 % |
| [[13](#_ENREF_13)] | 60 | 101 | 139 | 114 | 179 | 139 | -0.60 | -0.88; -0.24 | 7.90 % | 5.60 % |
| [[13](#_ENREF_13)] | 24 | 219 | 137 | 44 | 182 | 133 | 0.27 | -0.23; 0.77 | 3.20 % | 4.30 % |
| **Fixed effect** | | |  |  |  |  | **-0.20** | **-0.30; -0.12** | **100 %** | **-** |
| **Random effects** | | |  |  |  |  | **-0.20** | **-0.33; 0.00** | **-** | **100 %** |
| **Prediction range** | | |  |  |  |  | **-** | **-0.84; 0.52** |  |  |

##

# References

1. Arnett EB, Hayes JP. Use of conifer snags as roosts by female bats in western Oregon. Journal of Wildlife Management. 2009;73(2):214-25. doi: 10.2193/2007-532.

2. Clement MJ, Castleberry SB. Southeastern myotis (*Myotis austroriparius*) roost selection in cypress-gum swamps. Acta Chiropterologica. 2013;15(1):133-41. doi: 10.3161/150811013x667939.

3. Fabianek F, Simard MA, Racine B. E, Desrochers A. Selection of roosting habitat by male *Myotis* bats in a boreal forest. Canadian Journal of Zoology. 2015;(0):539-46. doi: 10.1139/cjz-2014-0294.

4. Fleming HL, Jones JC, Belant JL, Richardson DM. Multi-scale roost site selection by Rafinesque's big-eared bat (*Corynorhinus rafinesquii*) and southeastern myotis (*Myotis austroriparius*) in Mississippi. American Midland Naturalist. 2013;169(1):43-55. doi: 10.1674/0003-0031-169.1.43.

5. Herder MJ, Jackson JG. Roost preferences of long-legged myotis in northern Arizona. Transactions of the Western Section of the Wildlife Society. 2000;36:1-7.

6. Jung TS, Thompson ID, Titman RD. Roost site selection by forest-dwelling male *Myotis* in central Ontario, Canada. Forest Ecology and Management. 2004;202(1-3):325-35. doi: 10.1016/j.foreco.2004.07.043.

7. Lacki MJ, Schwierjohann JH. Day-roost characteristics of northern bats in mixed mesophytic forest. Journal of Wildlife Management. 2001;65(3):482-8. doi: 10.2307/3803101.

8. Miles AC, Castleberry SB, Miller DA, Conner LM. Multi-scale roost-site selection by evening bats on pine-dominated landscapes in southwest Georgia. Journal of Wildlife Management. 2006;70(5):1191-9. doi: 10.2193/0022-541x(2006)70[1191:mrsbeb]2.0.co;2.

9. Ormsbee PC, McComb WC. Selection of day roosts by female long-legged myotis in the central Oregon Cascade range. Journal of Wildlife Management. 1998;62(2):596-603. doi: 10.2307/3802335.

10. Psyllakis JM, Brigham RM. Characteristics of diurnal roosts used by female *Myotis* bats in sub-boreal forests. Forest Ecology and Management. 2006;223(1-3):93-102. doi: 10.1016/j.foreco.2005.03.071.

11. Rabe MJ, Morrell TE, Green H, Devos JJC, Miller CR. Characteristics of ponderosa pine snag roosts used by reproductive bats in northern Arizona. Journal of Wildlife Management. 1998;62:612-21. doi: 10.2307/3802337.

12. Weller TJ, Zabel CJ. Characteristics of fringed myotis day roosts in northern California. Journal of Wildlife Management. 2001;65(3):489-97. doi: 10.2307/3803102.

13. Boland JL, Hayes JP, Smith WP, Huso MM. Selection of day-roosts by Keen's myotis (*Myotis keenii*) at multiple spatial scales. Journal of Mammalogy. 2009; 90(1):222-34. doi: 10.1644/07-MAMM-A-369.1.
