# Supplementary material for: Exploring Regional Variation in Roost Selection by Bats: Evidence from a Meta-Analysis
Source: PLoS One. 2015 Sep 29;10(9):e0139126. doi: 10.1371/journal.pone.0139126 (PMC4587962; doi:10.1371/journal.pone.0139126)
Supplement: S7 Table — Number of selected and random trees is provided for each dataset with corresponding mean, standard deviation (SD), standardized mean difference (SMD) with 95% CI, fixed weight (W), and random weight. Fixed effect and random effects SMD with 95% CI, and prediction intervals are provided at the end of the table. All values are rounded upward to two decimal places. (DOCX) [file pone.0139126.s007.docx]

# Supporting information 7

## S7 Table. Meta-analysis tree density (%). Number of selected and random trees is provided for each dataset with corresponding mean, standard deviation (SD), standardized mean difference (SMD) with 95 % CI, fixed weight (W), and random weight. Fixed effect and random effects SMD with 95 % CI, and prediction intervals are provided at the end of the table. All values are rounded upward to two decimal places.

|  | **Selected trees** | | | **Random trees** | | |  |  |  |  |
| --- | --- | --- | --- | --- | --- | --- | --- | --- | --- | --- |
| **Study** | ***N*** | **Mean** | **SD** | ***N*** | **Mean** | **SD** | **SMD** | **95 % CI** | **W(fixed)** | **W(random)** |
| [[1](#_ENREF_1)] | 160 | 202 | 161.9 | 164 | 291.7 | 224.1 | -0.46 | -0.68; -0.23 | 15.7 % | 5.4 % |
| [[1](#_ENREF_1)] | 160 | 202 | 161.9 | 28 | 249.7 | 245.5 | -0.27 | -0.67; 0.13 | 4.7 % | 4.7 % |
| [[2](#_ENREF_2)] | 38 | 490 | 227.0 | 19 | 450.0 | 158.0 | 0.19 | -0.36; 0.74 | 2.5 % | 4.1 % |
| [[3](#_ENREF_3)] | 55 | 123 | 61.6 | 55 | 192.3 | 423.5 | -0.23 | -0.60; 0.15 | 5.4 % | 4.8 % |
| [[3](#_ENREF_3)] | 57 | 205 | 248.4 | 57 | 217.6 | 373.0 | -0.04 | -0.41; 0.33 | 5.7 % | 4.9 % |
| [[3](#_ENREF_3)] | 48 | 239 | 474.6 | 48 | 322.5 | 805.1 | -0.13 | -0.53; 0.27 | 4.8 % | 4.7 % |
| [[4](#_ENREF_4)] | 314 | 150 | 140.9 | 25 | 94.0 | 49.3 | 0.41 | 0.00; 0.82 | 4.6 % | 4.7 % |
| [[5](#_ENREF_5)] | 8 | 150 | 36.3 | 8 | 105.0 | 59.3 | 0.87 | -0.17; 1.91 | 0.7 % | 2.4 % |
| [[5](#_ENREF_5)] | 40 | 124 | 81.9 | 40 | 147.8 | 68.7 | -0.31 | -0.75; 0.13 | 3.9 % | 4.6 % |
| [[6](#_ENREF_6)] | 52 | 198 | 53.4 | 15 | 120.0 | 75.1 | 1.31 | 0.70; 1.93 | 2.0 % | 3.8 % |
| [[6](#_ENREF_6)] | 52 | 198 | 53.4 | 11 | 104.6 | 69.7 | 1.64 | 0.92; 2.35 | 1.5 % | 3.4 % |
| [[7](#_ENREF_7)] | 50 | 294 | 193.8 | 6 | 590.0 | 279.2 | -1.44 | -2.33; -0.55 | 1.0 % | 2.8 % |
| [[8](#_ENREF_8)] | 12 | 108 | 69.3 | 12 | 83.0 | 48.5 | 0.40 | -0.41; 1.21 | 1.2 % | 3.1 % |
| [[9](#_ENREF_9)] | 4 | 165 | 34.2 | 6 | 100.0 | 52.2 | 1.27 | -0.18; 2.72 | 0.4 % | 1.5 % |
| [[10](#_ENREF_10)] | 58 | 82 | 48.7 | 43 | 111.4 | 73.6 | -0.48 | -0.88; -0.08 | 4.8 % | 4.7 % |
| [[10](#_ENREF_10)] | 54 | 66.6 | 323.0 | 54 | 104.7 | 74.2 | -0.16 | -0.54; 0.22 | 5.4 % | 4.8 % |
| [[11](#_ENREF_11)] | 112 | 134 | 116.5 | 46 | 123.4 | 100.2 | 0.10 | -0.25; 0.44 | 6.5 % | 5.0 % |
| [[11](#_ENREF_11)] | 112 | 134 | 116.5 | 46 | 108.9 | 82.8 | 0.23 | -0.11; 0.58 | 6.5 % | 4.9 % |
| [[11](#_ENREF_11)] | 112 | 134 | 116.5 | 20 | 112.5 | 98.7 | 0.19 | -0.29; 0.67 | 3.4 % | 4.4 % |
| [[12](#_ENREF_12)] | 46 | 57.7 | 26.5 | 23 | 50.6 | 17.3 | 0.29 | -0.21; 0.80 | 3.0 % | 4.3 % |
| [[13](#_ENREF_13)] | 114 | 38.8 | 15.0 | 60 | 36.1 | 13.9 | 0.18 | -0.13; 0.50 | 7.8 % | 5.1 % |
| [[13](#_ENREF_13)] | 44 | 32.7 | 14.6 | 24 | 34.0 | 13.7 | -0.09 | -0.59; 0.41 | 3.1 % | 4.3 % |
| [[14](#_ENREF_14)] | 11 | 28 | 9.0 | 16 | 36.3 | 20.0 | -0.49 | -1.27; 0.29 | 1.3 % | 3.2 % |
| [[14](#_ENREF_14)] | 57 | 25.2 | 7.6 | 35 | 27.2 | 5.9 | -0.28 | -0.71; 0.14 | 4.3 % | 4.6 % |
| **Fixed effect** | | |  |  |  |  | **-0.04** | **-0.13; 0.04** | **100 %** | **-** |
| **Random effects** | | |  |  |  |  | **0.06** | **-0.15; 0.27** | **-** | **100 %** |
| **Prediction range** | | |  |  |  |  | - | **-0.88; 1.00** |  |  |

##

# References

1. Baker MD, Lacki MJ. Day-roosting habitat of female long-legged myotis in ponderosa pine forests. Journal of Wildlife Management. 2006;70(1):207-15. doi: 10.2307/3803562.

2. Brigham RM, Vonhof MJ, Barclay RMR, Gwilliam JC. Roosting behavior and roost-site preferences of forest-dwelling California bats (*Myotis californicus*). Journal of Mammalogy. 1997;78(4):1231-9. doi: 10.2307/1383066.

3. Broders HG, Forbes GJ. Interspecific and intersexual variation in roost-site selection of northern long-eared and little brown bats in the Greater Fundy National Park ecosystem. Journal of Wildlife Management. 2004;68(3):602-10. doi: 10.2193/0022-541x(2004)068[0602:iaivir]2.0.co;2.

4. Clement MJ, Castleberry SB. Southeastern myotis (*Myotis austroriparius*) roost selection in cypress-gum swamps. Acta Chiropterologica. 2013;15(1):133-41. doi: 10.3161/150811013x667939.

5. Fabianek F, Simard MA, Racine B. E, Desrochers A. Selection of roosting habitat by male *Myotis* bats in a boreal forest. Canadian Journal of Zoology. 2015;(0):539-46. doi: 10.1139/cjz-2014-0294.

6. Jung TS, Thompson ID, Titman RD. Roost site selection by forest-dwelling male *Myotis* in central Ontario, Canada. Forest Ecology and Management. 2004;202(1-3):325-35. doi: 10.1016/j.foreco.2004.07.043.

7. Lacki MJ, Baker MD. Day roosts of female fringed myotis (*Myotis thysanodes*) in xeric forests of the Pacific Northwest. Journal of Mammalogy. 2007;88(4):967-73. doi: 10.1644/06-MAMM-A-255R.1.

8. Menzel MA, Owen SF, Ford WM, Edwards JW, Wood PB, Chapman BR, et al. Roost tree selection by northern long-eared bat (*Myotis septentrionalis*) maternity colonies in an industrial forest of the central Appalachian mountains. Forest Ecology and Management. 2002;155(1):107-14. doi: 10.1016/S0378-1127(01)00551-5.

9. Parsons S, Lewis KJ, Psyllakis JM. Relationships between roosting habitat of bats and decay of aspen in the sub-boreal forests of British Columbia. Forest Ecology and Management. 2003;177(1–3):559-70. doi: 10.1016/S0378-1127(02)00448-6.

10. Rabe MJ, Morrell TE, Green H, Devos JJC, Miller CR. Characteristics of ponderosa pine snag roosts used by reproductive bats in northern Arizona. Journal of Wildlife Management. 1998;62:612-21. doi: 10.2307/3802337.

11. Vonhof MJ, Gwilliam JC. Intra- and interspecific patterns of day roost selection by three species of forest-dwelling bats in southern British Columbia. Forest Ecology and Management. 2007;252(1-3):165-75. doi: 10.1016/j.foreco.2007.06.046.

12. Weller TJ, Zabel CJ. Characteristics of fringed myotis day roosts in northern California. Journal of Wildlife Management. 2001;65(3):489-97. doi: 10.2307/3803102.

13. Boland JL, Hayes JP, Smith WP, Huso MM. Selection of day-roosts by Keen's myotis (*Myotis keenii*) at multiple spatial scales. Journal of Mammalogy. 2009; 90(1):222-34. doi: 10.1644/07-MAMM-A-369.1.

14. Lacki MJ, Cox DR, Dodd LE, Dickinson MB. Response of Northern bats (*Myotis septentrionalis*) to prescribed fires in eastern Kentucky forests. Journal of Mammalogy. 2009;90(5):1165-75. doi: 10.1644/08-MAMM-A-349.1.
