# Supplementary material for: Exploring Regional Variation in Roost Selection by Bats: Evidence from a Meta-Analysis
Source: PLoS One. 2015 Sep 29;10(9):e0139126. doi: 10.1371/journal.pone.0139126 (PMC4587962; doi:10.1371/journal.pone.0139126)
Supplement: S8 Table — Number of selected and random trees is provided for each dataset with corresponding mean, standard deviation (SD), standardized mean difference (SMD) with 95% CI, fixed weight (W), and random weight. Fixed effect and random effects SMD with 95% CI, and prediction intervals are provided at the end of the table. All values are rounded upward to two decimal places. (DOCX) [file pone.0139126.s008.docx]

# Supporting information 8

## S8 Table. Meta-analysis on slope (%). Number of selected and random trees is provided for each dataset with corresponding mean, standard deviation (SD), standardized mean difference (SMD) with 95 % CI, fixed weight (W), and random weight. Fixed effect and random effects SMD with 95 % CI, and prediction intervals are provided at the end of the table. All values are rounded upward to two decimal places.

|  | **Selected trees** | | | **Random trees** | | |  |  |  |  |
| --- | --- | --- | --- | --- | --- | --- | --- | --- | --- | --- |
| **Study** | ***N*** | **Mean** | **SD** | ***N*** | **Mean** | **SD** | **SMD** | **95 % CI** | **W(fixed)** | **W(random)** |
| [[1](#_ENREF_1)] | 105 | 32.4 | 20.5 | 119 | 36.8 | 25.1 | -0.19 | -0.45; 0.07 | 11.5 % | 6.3 % |
| [[1](#_ENREF_1)] | 24 | 36.4 | 23.5 | 23 | 42.8 | 23.5 | -0.27 | -0.84; 0.31 | 2.4 % | 4.2 % |
| [[1](#_ENREF_1)] | 42 | 35.6 | 22.7 | 104 | 33.8 | 20.4 | 0.08 | -0.27; 0.44 | 6.2 % | 5.7 % |
| [[1](#_ENREF_1)] | 35 | 42.3 | 28.4 | 33 | 35.6 | 20.1 | 0.27 | -0.21; 0.75 | 3.5 % | 4.8 % |
| [[1](#_ENREF_1)] | 22 | 34.1 | 18.8 | 26 | 40.0 | 33.7 | -0.21 | -0.78; 0.36 | 2.5 % | 4.3 % |
| [[2](#_ENREF_2)] | 164 | 26.7 | 25.6 | 160 | 25.6 | 21.5 | 0.05 | -0.17; 0.26 | 16.8 % | 6.5 % |
| [[2](#_ENREF_2)] | 28 | 31.2 | 25.9 | 160 | 25.6 | 21.5 | 0.25 | -0.15; 0.65 | 4.9 % | 5.4 % |
| [[3](#_ENREF_3)] | 55 | 6.6 | 3.7 | 55 | 7.4 | 4.5 | -0.19 | -0.57; 0.18 | 5.7 % | 5.5 % |
| [[3](#_ENREF_3)] | 57 | 4.6 | 4.5 | 57 | 5.4 | 10.6 | -0.10 | -0.47; 0.27 | 5.9 % | 5.6 % |
| [[3](#_ENREF_3)] | 48 | 4.6 | 4.2 | 48 | 4.9 | 4.9 | -0.07 | -0.47; 0.33 | 5.0 % | 5.4 % |
| [[4](#_ENREF_4)] | 8 | 3.3 | 2.5 | 157 | 9.3 | 17.5 | -0.35 | -1.06; 0.37 | 1.6 % | 3.5 % |
| [[4](#_ENREF_4)] | 7 | 2.6 | 1.9 | 147 | 9.3 | 14.8 | -0.46 | -1.22; 0.30 | 1.4 % | 3.2 % |
| [[5](#_ENREF_5)] | 52 | 4.0 | 5.1 | 61 | 6.1 | 5.5 | -0.39 | -0.77; -0.02 | 5.7 % | 5.6 % |
| [[6](#_ENREF_6)] | 16 | 29.1 | 11.7 | 6 | 43.5 | 12.5 | -1.16 | -2.17; -0.15 | 0.8 % | 2.3 % |
| [[7](#_ENREF_7)] | 6 | 52.3 | 15.3 | 50 | 34.3 | 20.6 | 0.88 | 0.02; 1.74 | 1.1 % | 2.8 % |
| [[8](#_ENREF_8)] | 12 | 23.3 | 7.9 | 12 | 25.3 | 6.1 | -0.28 | -1.08; 0.53 | 1.2 % | 3.0 % |
| [[9](#_ENREF_9)] | 43 | 14.1 | 11.5 | 58 | 10.5 | 13.3 | 0.28 | -0.12; 0.68 | 5.1 % | 5.4 % |
| [[9](#_ENREF_9)] | 54 | 29.1 | 12.9 | 54 | 12.3 | 12.9 | 1.30 | 0.88; 1.71 | 4.6 % | 5.3 % |
| [[10](#_ENREF_10)] | 23 | 29.7 | 24.9 | 46 | 35.7 | 14.9 | -0.32 | -0.82; 0.19 | 3.1 % | 4.7 % |
| [[11](#_ENREF_11)] | 60 | 43.0 | 14.7 | 114 | 36.9 | 16.0 | 0.39 | 0.07; 0.70 | 8.0 % | 5.9 % |
| [[11](#_ENREF_11)] | 24 | 35.6 | 14.7 | 44 | 31.5 | 15.3 | 0.27 | -0.23; 0.77 | 3.2 % | 4.7 % |
| **Fixed effect** | | |  |  |  |  | **0.05** | **-0.04; 0.14** | **100 %** | **-** |
| **Random effects** | | |  |  |  |  | **0.03** | **-0.16; 0.21** | **-** | **100 %** |
| **Prediction range** | | |  |  |  |  | **-** | **-0.73; 0.79** |  |  |

# References

1. Arnett EB, Hayes JP. Use of conifer snags as roosts by female bats in western Oregon. Journal of Wildlife Management. 2009;73(2):214-25. doi: 10.2193/2007-532.

2. Baker MD, Lacki MJ. Day-roosting habitat of female long-legged myotis in ponderosa pine forests. Journal of Wildlife Management. 2006;70(1):207-15. doi: 10.2307/3803562.

3. Broders HG, Forbes GJ. Interspecific and intersexual variation in roost-site selection of northern long-eared and little brown bats in the Greater Fundy National Park ecosystem. Journal of Wildlife Management. 2004;68(3):602-10. doi: 10.2193/0022-541x(2004)068[0602:iaivir]2.0.co;2.

4. Fleming HL, Jones JC, Belant JL, Richardson DM. Multi-scale roost site selection by Rafinesque's big-eared bat (*Corynorhinus rafinesquii*) and southeastern myotis (*Myotis austroriparius*) in Mississippi. American Midland Naturalist. 2013;169(1):43-55. doi: 10.1674/0003-0031-169.1.43.

5. Herder MJ, Jackson JG. Roost preferences of long-legged myotis in northern Arizona. Transactions of the Western Section of the Wildlife Society. 2000;36:1-7.

6. Johnson JB, Ford WM, Rodrigue JL, Edwards JW, Johnson CM. Roost selection by male Indiana myotis following forest fires in Central Appalachian hardwood forests. Journal of Fish and Wildlife Management. 2010;1(2):111-21. doi: 10.3996/042010-JFWM-007.

7. Lacki MJ, Baker MD. Day roosts of female fringed myotis (*Myotis thysanodes*) in xeric forests of the Pacific Northwest. Journal of Mammalogy. 2007;88(4):967-73. doi: 10.1644/06-MAMM-A-255R.1.

8. Menzel MA, Owen SF, Ford WM, Edwards JW, Wood PB, Chapman BR, et al. Roost tree selection by northern long-eared bat (*Myotis septentrionalis*) maternity colonies in an industrial forest of the central Appalachian mountains. Forest Ecology and Management. 2002;155(1):107-14. doi: 10.1016/S0378-1127(01)00551-5.

9. Rabe MJ, Morrell TE, Green H, Devos JJC, Miller CR. Characteristics of ponderosa pine snag roosts used by reproductive bats in northern Arizona. Journal of Wildlife Management. 1998;62:612-21. doi: 10.2307/3802337.

10. Weller TJ, Zabel CJ. Characteristics of fringed myotis day roosts in northern California. Journal of Wildlife Management. 2001;65(3):489-97. doi: 10.2307/3803102.

11. Boland JL, Hayes JP, Smith WP, Huso MM. Selection of day-roosts by Keen's myotis (*Myotis keenii*) at multiple spatial scales. Journal of Mammalogy. 2009; 90(1):222-34. doi: 10.1644/07-MAMM-A-369.1.
