# Supplementary material for: Exploring Regional Variation in Roost Selection by Bats: Evidence from a Meta-Analysis
Source: PLoS One. 2015 Sep 29;10(9):e0139126. doi: 10.1371/journal.pone.0139126 (PMC4587962; doi:10.1371/journal.pone.0139126)
Supplement: S9 Table — Number of selected and random trees is provided for each dataset with corresponding mean, standard deviation (SD), standardized mean difference (SMD) with 95% CI, fixed weight (W), and random weight. Fixed effect and random effects SMD with 95% CI, and prediction intervals are provided at the end of the table. All values are rounded upward to two decimal places. (DOCX) [file pone.0139126.s009.docx]

# Supporting information 9

## S9 Table. Meta-analysis on bark remaining on trunks (%). Number of selected and random trees is provided for each dataset with corresponding mean, standard deviation (SD), standardized mean difference (SMD) with 95 % CI, fixed weight (W), and random weight. Fixed effect and random effects SMD with 95 % CI, and prediction intervals are provided at the end of the table. All values are rounded upward to two decimal places.

|  | **Selected trees** | | | **Random trees** | | |  |  |  |  |
| --- | --- | --- | --- | --- | --- | --- | --- | --- | --- | --- |
| **Study** | ***N*** | **Mean** | **SD** | ***N*** | **Mean** | **SD** | **SMD** | **95 % CI** | **W(fixed)** | **W(random)** |
| [[1](#_ENREF_1)] | 164 | 64.6 | 44.8 | 160 | 29.6 | 34.2 | 0.87 | 0.65; 1.10 | 13.1 % | 4.1 % |
| [[1](#_ENREF_1)] | 28 | 82.5 | 47.1 | 160 | 29.6 | 34.2 | 1.45 | 1.02; 1.88 | 3.7 % | 4.0 % |
| [[2](#_ENREF_2)] | 19 | 75.0 | 29.6 | 38 | 86.0 | 27.3 | -0.39 | -0.94; 0.17 | 2.2 % | 3.8 % |
| [[3](#_ENREF_3)] | 55 | 78.5 | 30.4 | 55 | 73.7 | 33.4 | 0.15 | -0.22; 0.52 | 4.9 % | 4.0 % |
| [[3](#_ENREF_3)] | 57 | 74.4 | 31.7 | 57 | 59.0 | 37.8 | 0.44 | 0.07; 0.81 | 4.9 % | 4.0 % |
| [[3](#_ENREF_3)] | 48 | 72.1 | 31.2 | 48 | 69.0 | 34.6 | 0.09 | -0.31; 0.49 | 4.3 % | 4.0 % |
| [[4](#_ENREF_4)] | 11 | 81.1 | 16.9 | 14 | 76.2 | 16.5 | 0.28 | -0.51; 1.08 | 1.1 % | 3.5 % |
| [[4](#_ENREF_4)] | 20 | 69.1 | 18.3 | 5 | 69.8 | 23.0 | -0.04 | -1.02; 0.94 | 0.7 % | 3.2 % |
| [[5](#_ENREF_5)] | 47 | 47.0 | 26.7 | 37 | 55.0 | 35.3 | -0.26 | -0.69; 0.18 | 3.6 % | 4.0 % |
| [[5](#_ENREF_5)] | 19 | 79.2 | 30.9 | 46 | 63.8 | 38.4 | 0.42 | -0.12; 0.96 | 2.3 % | 3.8 % |
| [[6](#_ENREF_6)] | 8 | 93.1 | 15.8 | 8 | 70.6 | 34.9 | 0.79 | -0.24; 1.81 | 0.6 % | 3.1 % |
| [[6](#_ENREF_6)] | 40 | 0.7 | 26.7 | 40 | 54.5 | 39.7 | -1.57 | -2.08; -1.07 | 2.7 % | 3.9 % |
| [[7](#_ENREF_7)] | 34 | 76.8 | 31.0 | 15 | 46.7 | 31.8 | 0.95 | 0.31; 1.59 | 1.7 % | 3.7 % |
| [[8](#_ENREF_8)] | 15 | 61.0 | 19.8 | 52 | 77.0 | 32.5 | -0.52 | -1.11; 0.06 | 2.0 % | 3.8 % |
| [[8](#_ENREF_8)] | 11 | 63.6 | 27.2 | 52 | 77.0 | 32.5 | -0.42 | -1.07; 0.24 | 1.6 % | 3.7 % |
| [[9](#_ENREF_9)] | 56 | 68.8 | 33.1 | 1438 | 98.7 | 9.9 | -2.58 | -2.86; -2.30 | 8.5 % | 4.1 % |
| [[10](#_ENREF_10)] | 134 | 63.3 | 70.3 | 100 | 11.6 | 26.3 | 0.92 | 0.65; 1.20 | 9.2 % | 4.1 % |
| [[11](#_ENREF_11)] | 19 | 78.1 | 17.5 | 25 | 85.8 | 16.0 | -0.45 | -1.06; 0.15 | 1.9 % | 3.8 % |
| [[12](#_ENREF_12)] | 47 | 78.0 | 38.4 | 47 | 66.0 | 13.0 | 0.42 | 0.01; 0.82 | 4.1 % | 4.0 % |
| [[13](#_ENREF_13)] | 46 | 93.0 | 17.6 | 112 | 72.0 | 26.2 | 0.87 | 0.51; 1.23 | 5.4 % | 4.0 % |
| [[13](#_ENREF_13)] | 46 | 90.0 | 23.3 | 112 | 72.0 | 26.2 | 0.71 | 0.35; 1.06 | 5.5 % | 4.0 % |
| [[13](#_ENREF_13)] | 20 | 70.0 | 28.0 | 112 | 72.0 | 26.2 | -0.08 | -0.55; 0.40 | 3.0 % | 3.9 % |
| [[14](#_ENREF_14)] | 60 | 63.0 | 28.7 | 114 | 90.6 | 31.0 | -0.91 | -1.24; -0.58 | 6.4 % | 4.1 % |
| [[14](#_ENREF_14)] | 24 | 37.4 | 29.4 | 44 | 78.2 | 29.2 | -1.38 | -1.93; -0.83 | 2.2 % | 3.8 % |
| [[15](#_ENREF_15)] | 16 | 62.0 | 36.0 | 11 | 40.0 | 33.8 | 0.61 | -0.18; 1.39 | 1.1 % | 3.5 % |
| [[15](#_ENREF_15)] | 35 | 83.0 | 26.0 | 57 | 46.0 | 37.0 | 1.10 | 0.65; 1.55 | 3.3 % | 3.9 % |
| **Fixed effect** | | |  |  |  |  | **0.07** | **-0.01; 0.15** | **100 %** | **-** |
| **Random effects** | | |  |  |  |  | **0.05** | **-0.31; 0.41** | **-** | **100 %** |
| **Prediction range** | | |  |  |  |  | - | **-1.83; 1.93** |  |  |

# References

1. Baker MD, Lacki MJ. Day-roosting habitat of female long-legged myotis in ponderosa pine forests. Journal of Wildlife Management. 2006;70(1):207-15. doi: 10.2307/3803562.

2. Brigham RM, Vonhof MJ, Barclay RMR, Gwilliam JC. Roosting behavior and roost-site preferences of forest-dwelling California bats (*Myotis californicus*). Journal of Mammalogy. 1997;78(4):1231-9. doi: 10.2307/1383066.

3. Broders HG, Forbes GJ. Interspecific and intersexual variation in roost-site selection of northern long-eared and little brown bats in the Greater Fundy National Park ecosystem. Journal of Wildlife Management. 2004;68(3):602-10. doi: 10.2193/0022-541x(2004)068[0602:iaivir]2.0.co;2.

4. Callahan EV, Drobney RD, Clawson RL. Selection of summer roosting sites by Indiana bats (*Myotis sodalis*) in Missouri. Journal of Mammalogy. 1997;78(3):818-25. doi: 10.2307/1382939.

5. Carter TC. Summer habitat use of roost trees by the endangered Indiana bat *(Myotis sodalis*) in the Shawnee National Forest of southern Illinois. Southern Illinois: Carbondale University; 2003.

6. Fabianek F, Simard MA, Racine B. E, Desrochers A. Selection of roosting habitat by male *Myotis* bats in a boreal forest. Canadian Journal of Zoology. 2015;(0):539-46. doi: 10.1139/cjz-2014-0294.

7. Johnson JB, Ford WM, Rodrigue JL, Edwards JW, Johnson CM. Roost selection by male Indiana myotis following forest fires in Central Appalachian hardwood forests. Journal of Fish and Wildlife Management. 2010;1(2):111-21. doi: 10.3996/042010-JFWM-007.

8. Jung TS, Thompson ID, Titman RD. Roost site selection by forest-dwelling male *Myotis* in central Ontario, Canada. Forest Ecology and Management. 2004;202(1-3):325-35. doi: 10.1016/j.foreco.2004.07.043.

9. Kniowski AB, Gehrt SD. Summer ecology of Indiana bats in Ohio. Columbus, OH, USA: 2011.

10. Lacki MJ, Baker MD, Johnson JS. Geographic variation in roost-site selection of long-legged myotis in the Pacific Northwest. Journal of Wildlife Management. 2010;74(6):1218-28. doi: 10.2307/40801116.

11. Psyllakis JM, Brigham RM. Characteristics of diurnal roosts used by female *Myotis* bats in sub-boreal forests. Forest Ecology and Management. 2006;223(1-3):93-102. doi: 10.1016/j.foreco.2005.03.071.

12. Sasse DB, Pekins PJ. Summer roosting ecology of northern long-eared bats (*Myotis septentrionalis*) in the White Mountain National Forest. In: Barclay RMR, Brigham RM, editors. Bats and Forests Symposium; October 19-21, 1995; Organized by the British Columbia Ministry of Forests. Victoria, BC1996. p. 91-101.

13. Vonhof MJ, Gwilliam JC. Intra- and interspecific patterns of day roost selection by three species of forest-dwelling bats in southern British Columbia. Forest Ecology and Management. 2007;252(1-3):165-75. doi: 10.1016/j.foreco.2007.06.046.

14. Boland JL, Hayes JP, Smith WP, Huso MM. Selection of day-roosts by Keen's myotis (*Myotis keenii*) at multiple spatial scales. Journal of Mammalogy. 2009; 90(1):222-34. doi: 10.1644/07-MAMM-A-369.1.

15. Lacki MJ, Cox DR, Dodd LE, Dickinson MB. Response of Northern bats (*Myotis septentrionalis*) to prescribed fires in eastern Kentucky forests. Journal of Mammalogy. 2009;90(5):1165-75. doi: 10.1644/08-MAMM-A-349.1.
