# Supplementary material for: Expression of Concern: Exploring Regional Variation in Roost Selection by Bats: Evidence from a Meta-Analysis
Source: PLoS One. 2024 Dec 18;19(12):e0316243. doi: 10.1371/journal.pone.0316243 (PMC11654921; doi:10.1371/journal.pone.0316243)
Supplement: S1 File — Revised meta-analyses, Table 1, and Figs 3–4 (PDF) [file pone.0316243.s001.pdf]

Tree Diameter

Study	Experimental Total	Experimental Mean	Experimental SD	Control Total	Control Mean	Control SD	SMD	95%-CI	Weight (fixed)	Weight (random)
Arnett_and_Hayes	42	121.60	42.7700	104	76.10	41.8100	1.08	[0.70; 1.46]	2.6%	1.9%
Arnett_and_Hayes	35	138.70	47.9200	33	96.80	41.3600	0.92	[0.42; 1.43]	1.5%	1.7%
Arnett_and_Hayes	24	62.40	37.2300	23	69.20	35.4900	-0.18	[-0.76; 0.39]	1.1%	1.6%
Arnett_and_Hayes	105	91.10	49.1900	119	72.10	39.2700	0.43	[0.16; 0.69]	5.3%	2.1%
Arnett_and_Hayes	22	95.50	50.6600	26	98.70	54.5600	-0.06	[-0.63; 0.51]	1.1%	1.6%
Baker_and_Lacki	164	60.90	24.3300	160	54.00	21.5000	0.30	[0.08; 0.52]	7.7%	2.1%
Baker_and_Lacki	28	82.30	19.0500	160	54.00	21.5000	1.33	[0.91; 1.76]	2.1%	1.8%
Brigham_et_al	19	56.00	16.7800	38	28.00	15.0000	1.77	[1.12; 2.42]	0.9%	1.5%
Broders_and_Forbes	48	32.90	10.3900	48	30.00	11.0900	0.27	[-0.13; 0.67]	2.3%	1.9%
Broders_and_Forbes	55	43.80	13.3500	55	38.30	14.0900	0.40	[0.02; 0.78]	2.6%	1.9%
Broders_and_Forbes	57	32.00	12.0800	57	27.20	11.3200	0.41	[0.04; 0.78]	2.7%	1.9%
Callahan_et_al	7	49.30	16.4000	8	40.00	14.9900	0.56	[-0.48; 1.60]	0.3%	1.0%
Callahan_et_al	11	54.50	15.5900	14	38.50	14.5900	1.03	[0.18; 1.88]	0.5%	1.2%
Callahan_et_al	20	54.60	13.8600	5	46.20	13.9500	0.59	[-0.41; 1.58]	0.4%	1.0%
Carter	19	37.29	20.5700	46	34.91	14.9900	0.14	[-0.39; 0.68]	1.3%	1.7%
Carter	47	39.02	13.9900	37	37.71	13.5600	0.09	[-0.34; 0.53]	2.0%	1.8%
Clement_and_Castleberry	25	93.50	30.0000	314	63.30	34.7000	0.88	[0.46; 1.29]	2.2%	1.9%
Cryan_et_al	8	42.10	7.4000	30	31.70	12.4000	0.88	[0.07; 1.69]	0.6%	1.3%
Cryan_et_al	21	39.00	8.2000	30	31.70	12.4000	0.66	[0.09; 1.23]	1.1%	1.6%
Cryan_et_al	9	43.30	12.1000	30	31.70	12.4000	0.92	[0.15; 1.70]	0.6%	1.3%
Cryan_et_al	7	42.60	3.2000	30	31.70	12.4000	0.94	[0.09; 1.79]	0.5%	1.2%
Fabianek_et_al	8	48.39	8.7400	8	31.04	7.5700	2.01	[0.74; 3.27]	0.2%	0.8%
Fabianek_et_al	40	20.69	4.4800	40	15.53	5.3600	1.03	[0.57; 1.50]	1.7%	1.8%
Fleming_et_al	8	74.90	28.8500	157	65.70	26.3100	0.35	[-0.36; 1.06]	0.7%	1.4%
Fleming_et_al	7	92.00	42.8600	147	63.70	23.0400	1.17	[0.40; 1.94]	0.6%	1.3%
Foster_and_Kurta	29	63.00	32.3100	46	37.70	12.8200	1.12	[0.62; 1.62]	1.5%	1.7%
Foster_and_Kurta	23	41.00	4.8000	46	37.70	12.8200	0.30	[-0.20; 0.80]	1.5%	1.7%
Grindal_et_al	8	29.10	7.3500	241	23.60	7.4500	0.74	[0.03; 1.44]	0.7%	1.4%
Grindal_et_al	8	29.10	7.3500	84	25.90	10.0800	0.32	[-0.41; 1.05]	0.7%	1.4%
Herder_and_Jackson	52	79.90	21.6300	61	62.20	31.2400	0.65	[0.27; 1.02]	2.6%	1.9%
Johnson_et_al	16	41.37	10.1600	6	26.63	11.8800	1.34	[0.30; 2.37]	0.3%	1.0%
Jung_et_al	15	42.60	14.7200	52	28.10	5.7700	1.68	[1.03; 2.32]	0.9%	1.5%
Jung_et_al	11	49.50	19.2400	52	28.10	5.7700	2.25	[1.48; 3.01]	0.6%	1.3%
Kniowski_and_Gehrt	56	47.30	16.6900	16	41.00	12.4800	0.39	[-0.17; 0.95]	1.2%	1.6%
Lacki_and_Baker	6	82.60	26.2100	50	52.80	19.2300	1.47	[0.58; 2.36]	0.5%	1.2%
Menzel_et_al	12	29.20	5.5400	12	34.70	10.0500	-0.65	[-1.48; 0.17]	0.5%	1.2%
Miles_et_al	111	50.00	18.9600	111	42.10	26.3400	0.34	[0.08; 0.61]	5.3%	2.1%
Miles_et_al	57	26.30	9.8100	57	19.90	7.5500	0.73	[0.35; 1.11]	2.6%	1.9%
Ormsbee_and_McComb	33	97.00	38.0800	66	73.00	28.7600	0.74	[0.31; 1.17]	2.0%	1.8%
Parsons_et_al	6	49.60	12.7400	4	39.30	8.0000	0.83	[-0.52; 2.18]	0.2%	0.7%
Perry_and_Till	40	15.00	8.2200	82	11.80	6.3400	0.45	[0.07; 0.84]	2.5%	1.9%
Perry_and_Till	42	18.70	6.4800	82	11.80	6.3400	1.07	[0.68; 1.47]	2.4%	1.9%
Psyllakis_and_Brigham	19	26.13	7.2400	25	23.64	7.3000	0.34	[-0.26; 0.94]	1.0%	1.6%
Psyllakis_and_Brigham	17	48.15	10.5100	21	36.95	11.7300	0.98	[0.30; 1.66]	0.8%	1.5%
Rabe_et_al	43	66.00	11.8000	58	57.40	16.7500	0.57	[0.17; 0.98]	2.3%	1.9%
Rabe_et_al	54	69.00	14.7000	54	58.60	15.4300	0.69	[0.30; 1.07]	2.5%	1.9%
Sasse_and_Pekins	47	40.90	19.2000	47	33.00	6.8600	0.54	[0.13; 0.96]	2.2%	1.9%
Vonhof_and_Gwilliam	46	49.00	15.6000	112	36.30	13.6400	0.89	[0.53; 1.25]	2.9%	1.9%
Vonhof_and_Gwilliam	46	40.00	13.4300	112	36.30	13.6400	0.27	[-0.07; 0.62]	3.1%	2.0%
Vonhof_and_Gwilliam	20	55.80	14.2000	112	36.30	13.6400	1.41	[0.91; 1.92]	1.4%	1.7%
Weller_and_Zabel	23	120.80	25.4200	46	78.50	46.1200	1.03	[0.50; 1.56]	1.3%	1.7%
Perry_and_Till	9	20.60	6.6000	34	15.30	7.0000	0.75	[0.00; 1.50]	0.7%	1.3%
Perry_and_Till	25	21.50	5.0000	34	15.30	7.0000	0.98	[0.43; 1.53]	1.2%	1.7%
Boland_et_al	62	106.50	34.6400	114	50.70	35.2300	1.59	[1.23; 1.94]	3.0%	2.0%
Boland_et_al	24	65.60	34.7800	44	49.90	33.1700	0.46	[-0.04; 0.96]	1.5%	1.7%
Hein	31	22.30	13.3600	31	28.80	11.6900	-0.51	[-1.02; 0.00]	1.4%	1.7%
Hein	24	36.60	14.2100	24	31.20	10.7800	0.42	[-0.15; 0.99]	1.1%	1.6%
Hein	11	32.40	8.9500	11	31.70	12.2700	0.06	[-0.77; 0.90]	0.5%	1.2%
Hein	9	23.00	13.8000	9	32.50	11.1000	-0.72	[-1.68; 0.24]	0.4%	1.1%
Lacki_et_al	16	44.20	16.0000	11	41.60	21.8900	0.14	[-0.63; 0.90]	0.6%	1.3%
Lacki_et_al	35	34.60	20.1100	57	32.00	13.5900	0.16	[-0.26; 0.58]	2.1%	1.9%
Barclay_et_al	27	51.57	16.5200	25	28.65	7.9500	1.72	[1.08; 2.36]	0.9%	1.5%
Barclay_et_al	6	46.79	17.1500	50	21.96	8.9800	2.44	[1.48; 3.41]	0.4%	1.1%
**Fixed effect model**	**1955**			**3818**			**0.64**	**[0.57; 0.70]**	**100.0%**	**--**
**Random effects model**							**0.70**	**[0.56; 0.84]**	**--**	**100.0%**
**Prediction interval**								**[-0.26; 1.66]**		

Heterogeneity: $I^2 = 75\%$, $\tau^2 = 0.2246$, $p < 0.01$

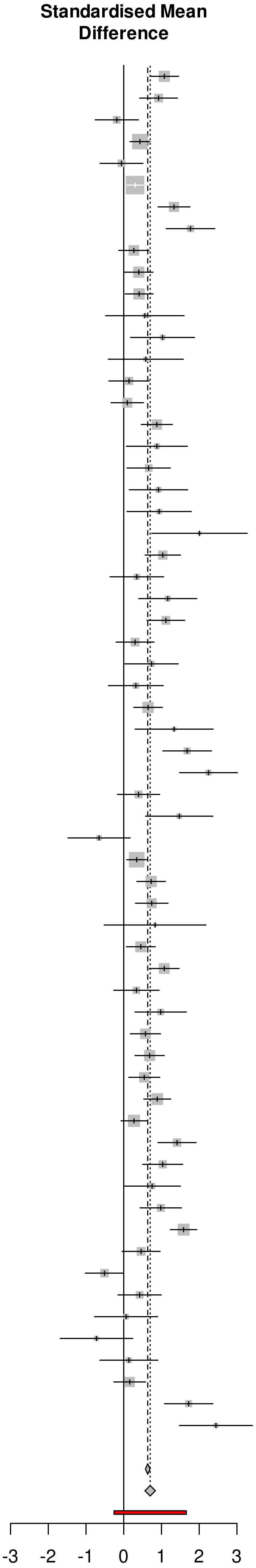

Tree Height

Study	Experimental Total	Experimental Mean	Experimental SD	Control Total	Control Mean	Control SD	SMD	95%-CI	Weight (fixed)	Weight (random)
Arnett_and_Hayes	105	22.80	9.2200	119	23.10	13.0900	-0.03	[-0.29; 0.24]	6.5%	2.6%
Arnett_and_Hayes	24	17.70	10.7800	23	21.90	12.4700	-0.35	[-0.93; 0.22]	1.3%	2.1%
Arnett_and_Hayes	42	24.30	9.0700	104	21.90	13.2600	0.20	[-0.16; 0.55]	3.5%	2.5%
Arnett_and_Hayes	35	34.90	8.8700	33	32.30	11.4900	0.25	[-0.23; 0.73]	2.0%	2.3%
Arnett_and_Hayes	22	34.30	11.2600	26	36.80	17.3400	-0.17	[-0.73; 0.40]	1.4%	2.1%
Baker_and_Lacki	164	26.70	7.6800	160	14.70	8.8500	1.45	[1.20; 1.69]	7.4%	2.6%
Baker_and_Lacki	28	31.50	12.1700	160	14.70	8.8500	1.78	[1.34; 2.22]	2.3%	2.3%
Brigham_et_al	19	27.00	7.9000	38	17.00	8.9000	1.15	[0.56; 1.74]	1.3%	2.1%
Broders_and_Forbes	55	13.50	3.7100	55	12.40	5.1900	0.24	[-0.13; 0.62]	3.2%	2.4%
Broders_and_Forbes	57	11.50	3.7700	57	10.70	4.5300	0.19	[-0.18; 0.56]	3.3%	2.4%
Broders_and_Forbes	48	12.20	4.8500	48	11.80	4.8500	0.08	[-0.32; 0.48]	2.8%	2.4%
Carter	47	17.53	6.5100	37	15.65	6.0800	0.29	[-0.14; 0.73]	2.4%	2.3%
Carter	19	15.76	8.5400	46	14.90	5.7600	0.13	[-0.41; 0.66]	1.6%	2.2%
Clement_and_Castleberry	25	24.80	4.3000	314	21.40	7.0000	0.50	[0.09; 0.90]	2.7%	2.4%
Cryan_et_al	21	9.10	5.9000	30	11.40	7.1000	-0.34	[-0.90; 0.22]	1.4%	2.1%
Cryan_et_al	8	22.20	2.5000	30	11.40	7.1000	1.63	[0.76; 2.51]	0.6%	1.6%
Cryan_et_al	9	6.90	6.3000	30	11.40	7.1000	-0.64	[-1.40; 0.12]	0.8%	1.8%
Cryan_et_al	7	11.00	7.1000	30	11.40	7.1000	-0.06	[-0.88; 0.77]	0.7%	1.7%
Fabianek_et_al	8	15.76	2.9800	8	14.81	2.9500	0.30	[-0.68; 1.29]	0.5%	1.5%
Fabianek_et_al	40	9.57	3.1400	40	6.63	3.0000	0.95	[0.48; 1.41]	2.1%	2.3%
Herder_and_Jackson	52	21.10	7.2100	61	16.10	9.3700	0.59	[0.21; 0.97]	3.1%	2.4%
Johnson_et_al	16	21.65	4.4400	6	12.92	5.3200	1.80	[0.69; 2.90]	0.4%	1.3%
Jung_et_al	15	20.10	4.2600	52	15.50	3.6100	1.21	[0.60; 1.82]	1.2%	2.0%
Jung_et_al	11	21.00	6.9600	52	15.50	3.6100	1.25	[0.56; 1.94]	0.9%	1.9%
Lacki_and_Baker	6	31.30	9.3300	50	14.40	9.1200	1.82	[0.91; 2.74]	0.5%	1.6%
Lacki_et_al	134	28.30	7.4100	100	15.30	8.9000	1.60	[1.31; 1.90]	5.0%	2.5%
Menzel_et_al	12	18.70	5.2000	12	16.80	7.2700	0.29	[-0.51; 1.10]	0.7%	1.7%
Miles_et_al	111	22.20	5.2700	111	19.50	6.3200	0.46	[0.20; 0.73]	6.3%	2.6%
Miles_et_al	57	15.40	6.7900	57	13.60	3.7700	0.33	[-0.04; 0.70]	3.3%	2.4%
Psyllakis_and_Brigham	19	22.57	8.1500	25	18.82	9.2000	0.42	[-0.18; 1.02]	1.2%	2.1%
Psyllakis_and_Brigham	17	26.38	9.0700	21	20.21	8.4800	0.69	[0.03; 1.35]	1.0%	2.0%
Rabe_et_al	43	18.80	6.5600	58	17.70	8.3800	0.14	[-0.25; 0.54]	2.9%	2.4%
Rabe_et_al	54	17.80	8.0800	54	15.90	8.8200	0.22	[-0.16; 0.60]	3.1%	2.4%
Sasse_and_Pekins	47	14.80	6.8600	47	8.80	2.7400	1.14	[0.70; 1.58]	2.3%	2.3%
Vonhof_and_Gwilliam	46	23.70	8.3600	112	18.60	10.0200	0.53	[0.18; 0.88]	3.7%	2.5%
Vonhof_and_Gwilliam	46	29.40	9.0400	112	18.60	10.0200	1.10	[0.74; 1.47]	3.4%	2.4%
Vonhof_and_Gwilliam	20	29.30	6.7200	112	18.60	10.0200	1.11	[0.61; 1.60]	1.8%	2.2%
Perry_and_Till	9	12.30	4.2000	34	6.50	5.2500	1.12	[0.35; 1.90]	0.7%	1.8%
Perry_and_Till	25	12.00	4.5000	34	6.50	5.2500	1.10	[0.54; 1.65]	1.4%	2.1%
Hein	31	16.60	7.2400	31	19.30	6.6800	-0.38	[-0.89; 0.12]	1.8%	2.2%
Hein	24	24.90	4.9000	24	22.40	6.8600	0.41	[-0.16; 0.98]	1.4%	2.1%
Hein	11	22.30	4.9700	11	20.30	7.9600	0.29	[-0.55; 1.13]	0.6%	1.7%
Hein	9	16.90	9.6000	9	20.20	8.1000	-0.35	[-1.29; 0.58]	0.5%	1.5%
Lacki_et_al	16	20.60	10.0000	11	12.10	6.9600	0.93	[0.11; 1.74]	0.7%	1.7%
Lacki_et_al	35	19.70	8.2800	57	17.80	7.5500	0.24	[-0.18; 0.66]	2.5%	2.4%
Barclay_et_al	27	14.83	8.0500	25	15.26	2.9500	-0.07	[-0.61; 0.48]	1.5%	2.2%
Barclay_et_al	6	12.85	4.9500	50	12.46	3.1100	0.12	[-0.73; 0.96]	0.6%	1.7%
**Fixed effect model**	**1682**			**2716**			**0.57**	**[0.50; 0.64]**	**100.0%**	**--**
**Random effects model**							**0.54**	**[0.36; 0.71]**	**--**	**100.0%**
**Prediction interval**								**[-0.56; 1.63]**		

Heterogeneity: $I^2 = 85\%$, $\tau^2 = 0.2872$, $p < 0.01$

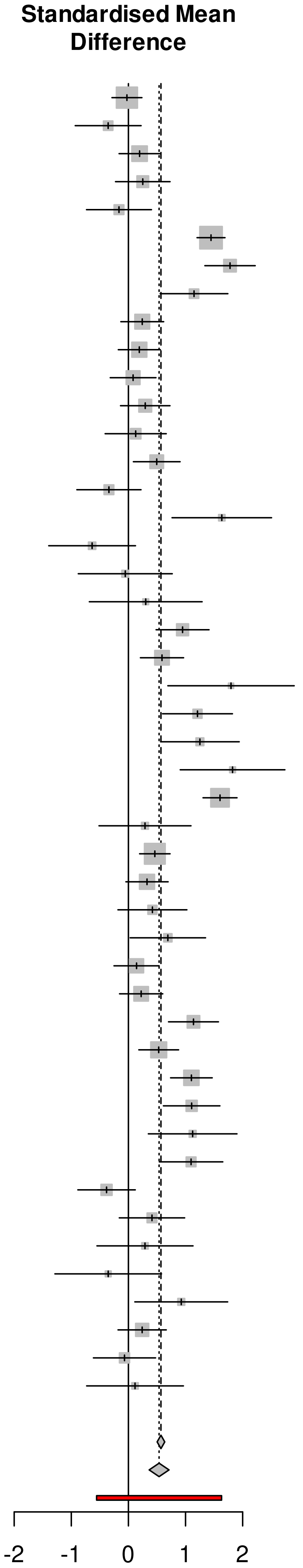

Number of snags

Study	Experimental Total	Experimental Mean	Experimental SD	Control Total	Control Mean	Control SD	SMD	95%-CI	Weight (fixed)	Weight (random)
Arnett_and_Hayes	105	2.30	3.0700	119	0.50	1.0900	0.80	[0.53; 1.07]	7.7%	4.0%
Arnett_and_Hayes	24	1.50	2.4500	23	0.60	0.9600	0.47	[-0.11; 1.05]	1.7%	2.6%
Arnett_and_Hayes	42	2.30	1.9400	104	0.70	2.0400	0.79	[0.42; 1.16]	4.2%	3.6%
Arnett_and_Hayes	35	1.80	1.7700	33	1.00	1.7200	0.45	[-0.03; 0.93]	2.5%	3.1%
Arnett_and_Hayes	22	1.40	1.8800	26	0.90	1.5300	0.29	[-0.28; 0.86]	1.8%	2.7%
Baker_and_Lacki	164	42.70	46.1000	160	16.60	22.7700	0.71	[0.49; 0.94]	11.3%	4.2%
Baker_and_Lacki	28	41.20	84.1300	160	16.60	22.7700	0.64	[0.23; 1.05]	3.4%	3.4%
Brigham_et_al	19	55.00	37.0000	38	50.00	3.0000	0.23	[-0.32; 0.78]	1.9%	2.8%
Broders_and_Forbes	55	7.00	5.1900	55	6.90	5.9300	0.02	[-0.36; 0.39]	4.1%	3.6%
Broders_and_Forbes	57	11.00	7.5500	57	9.30	6.0400	0.25	[-0.12; 0.62]	4.2%	3.6%
Broders_and_Forbes	48	12.70	6.9300	48	9.00	4.8500	0.61	[0.20; 1.02]	3.4%	3.4%
Cryan_et_al	21	0.30	0.6000	30	0.10	0.4000	0.40	[-0.16; 0.96]	1.8%	2.7%
Cryan_et_al	8	0.60	0.7000	30	0.10	0.4000	1.03	[0.22; 1.85]	0.9%	1.8%
Cryan_et_al	9	0.50	0.8000	30	0.10	0.4000	0.76	[0.00; 1.53]	1.0%	2.0%
Cryan_et_al	7	0.90	1.0000	30	0.10	0.4000	1.42	[0.53; 2.31]	0.7%	1.7%
Fabianek_et_al	8	1.00	1.7700	8	1.00	1.0700	0.00	[-0.98; 0.98]	0.6%	1.5%
Fabianek_et_al	40	6.40	4.3700	40	1.93	2.6200	1.23	[0.75; 1.71]	2.5%	3.1%
Herder_and_Jackson	52	6.40	9.0900	61	6.80	7.7300	-0.05	[-0.42; 0.32]	4.2%	3.6%
Jung_et_al	15	32.70	22.4600	52	33.70	17.3100	-0.05	[-0.63; 0.52]	1.7%	2.7%
Jung_et_al	11	25.50	14.2600	52	33.70	17.3100	-0.48	[-1.14; 0.18]	1.3%	2.4%
Lacki_and_Baker	6	7.00	3.1000	50	8.00	6.2000	-0.16	[-1.01; 0.68]	0.8%	1.8%
Menzel_et_al	12	1.80	0.1300	12	1.10	1.7000	0.56	[-0.26; 1.38]	0.9%	1.8%
Psyllakis_and_Brigham	17	56.71	38.1800	21	34.76	20.9000	0.72	[0.06; 1.38]	1.3%	2.3%
Rabe_et_al	43	6.50	6.5600	58	1.00	3.0500	1.12	[0.70; 1.55]	3.2%	3.3%
Rabe_et_al	54	10.60	10.2900	54	2.00	4.4100	1.08	[0.67; 1.48]	3.5%	3.4%
Sasse_and_Pekins	47	3.90	2.0600	47	2.30	2.0600	0.77	[0.35; 1.19]	3.2%	3.4%
Vonhof_and_Gwilliam	46	7.00	7.4000	112	6.00	5.0000	0.17	[-0.17; 0.52]	4.8%	3.7%
Vonhof_and_Gwilliam	46	8.00	5.6000	112	6.00	5.0000	0.38	[0.04; 0.73]	4.8%	3.7%
Vonhof_and_Gwilliam	20	7.00	3.7000	112	6.00	5.0000	0.21	[-0.27; 0.68]	2.5%	3.1%
Weller_and_Zabel	23	10.80	4.3200	46	11.30	14.9200	-0.04	[-0.54; 0.46]	2.3%	3.0%
Boland_et_al	62	0.38	0.1500	114	0.36	0.1100	0.16	[-0.15; 0.47]	5.8%	3.9%
Boland_et_al	24	0.49	0.1500	44	0.38	0.1300	0.79	[0.28; 1.31]	2.1%	2.9%
Lacki_et_al	16	4.05	2.6800	11	2.75	2.8900	0.46	[-0.32; 1.23]	0.9%	2.0%
Lacki_et_al	35	2.52	1.8300	57	3.29	3.9300	-0.23	[-0.65; 0.19]	3.2%	3.3%
**Fixed effect model**	**1219**			**2006**			**0.48**	**[0.40; 0.55]**	**100.0%**	**--**
**Random effects model**							**0.46**	**[0.31; 0.60]**	**--**	**100.0%**
**Prediction interval**								**[-0.24; 1.15]**		

Heterogeneity: $I^2 = 69\%$, $\tau^2 = 0.1114$, $p < 0.01$

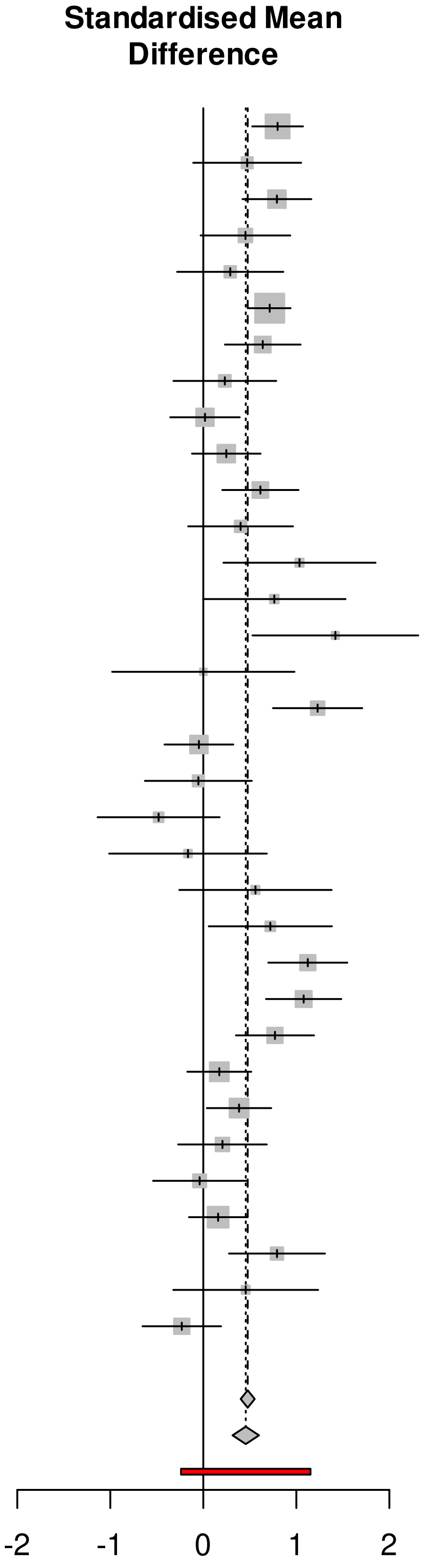

Elevation

Study	Experimental Total	Experimental Mean	Experimental SD	Control Total	Control Mean	Control SD	SMD	95%-CI	Weight (fixed)	Weight (random)
Arnett_and_Hayes	105	624.00	194.6900	119	665.00	229.0800	-0.19	[-0.45; 0.07]	13.2%	8.3%
Arnett_and_Hayes	24	554.00	151.8700	23	578.00	139.0800	-0.16	[-0.73; 0.41]	2.8%	4.2%
Arnett_and_Hayes	42	508.00	194.4200	104	737.00	305.9400	-0.82	[-1.19; -0.45]	6.6%	6.6%
Arnett_and_Hayes	35	562.00	165.6500	33	771.00	338.9300	-0.78	[-1.28; -0.29]	3.7%	5.0%
Arnett_and_Hayes	22	480.00	150.0900	26	613.00	163.1700	-0.83	[-1.43; -0.24]	2.6%	4.0%
Baker_and_Lacki	164	1217.00	322.7200	160	1325.00	320.0200	-0.34	[-0.55; -0.12]	19.0%	9.0%
Baker_and_Lacki	28	1263.00	360.8800	160	1325.00	320.0200	-0.19	[-0.59; 0.21]	5.6%	6.1%
Fabianek_et_al	40	710.05	48.4300	40	751.95	69.1900	-0.69	[-1.15; -0.24]	4.5%	5.5%
Fleming_et_al	33	229.80	30.4500	139	275.50	96.6800	-0.52	[-0.90; -0.13]	6.2%	6.4%
Fleming_et_al	19	218.30	8.7000	144	275.40	94.8000	-0.64	[-1.12; -0.15]	3.9%	5.1%
Herder_and_Jackson	52	2068.00	264.0000	61	2093.00	152.4000	-0.12	[-0.49; 0.25]	6.7%	6.6%
Johnson_et_al	16	833.56	130.3200	6	680.17	315.1200	0.76	[-0.21; 1.73]	1.0%	1.9%
Johnson_et_al	34	812.59	199.9200	15	721.27	246.9600	0.42	[-0.20; 1.03]	2.4%	3.8%
Jung_et_al	15	400.00	60.0000	52	430.00	60.0000	-0.49	[-1.08; 0.09]	2.7%	4.1%
Jung_et_al	11	400.00	60.0000	52	430.00	60.0000	-0.49	[-1.15; 0.16]	2.1%	3.5%
Lacki_and_Baker	6	957.00	637.2000	50	1218.00	285.6000	-0.77	[-1.63; 0.09]	1.2%	2.3%
Rabe_et_al	43	2099.00	144.0000	58	2142.00	156.0000	-0.28	[-0.68; 0.11]	5.8%	6.2%
Rabe_et_al	54	2366.00	192.0000	54	2341.00	168.0000	0.14	[-0.24; 0.52]	6.4%	6.5%
Weller_and_Zabel	23	1058.40	218.4000	46	1072.40	139.2000	-0.08	[-0.58; 0.42]	3.6%	4.9%
**Fixed effect model**	**766**			**1342**			**-0.33**	**[-0.43; -0.24]**	**100.0%**	**--**
**Random effects model**							**-0.34**	**[-0.49; -0.19]**	**--**	**100.0%**
**Prediction interval**								**[-0.84; 0.16]**		

Heterogeneity: $I^2 = 55\%$, $\tau^2 = 0.0506$, $p < 0.01$

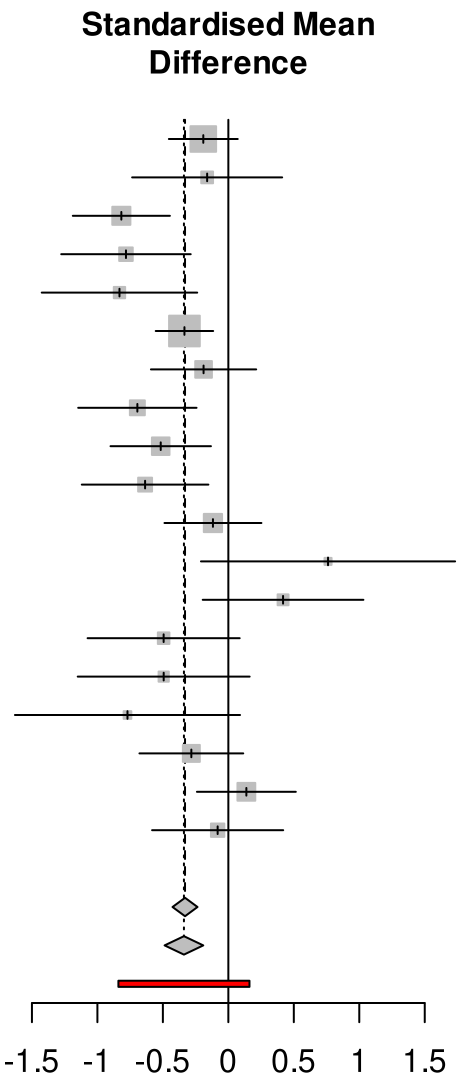

Canopy closure

Study	Experimental Total	Experimental Mean	Experimental SD	Control Total	Control Mean	Control SD	SMD	95%-CI	Weight (fixed)	Weight (random)
Arnett_and_Hayes	105	27.50	16.4000	119	43.60	24.0000	-0.77	[-1.04; -0.50]	7.6%	3.5%
Arnett_and_Hayes	24	24.80	17.6400	23	34.60	24.4600	-0.45	[-1.03; 0.13]	1.7%	2.9%
Arnett_and_Hayes	42	30.70	16.2000	104	45.40	23.4600	-0.68	[-1.04; -0.31]	4.2%	3.3%
Arnett_and_Hayes	35	37.90	17.1600	33	35.60	21.8300	0.12	[-0.36; 0.59]	2.5%	3.1%
Arnett_and_Hayes	22	33.90	11.2600	26	39.40	22.9500	-0.29	[-0.86; 0.28]	1.7%	2.9%
Baker_and_Lacki	164	31.20	24.3300	160	28.50	21.5000	0.12	[-0.10; 0.34]	11.9%	3.5%
Baker_and_Lacki	28	22.10	20.6400	160	28.50	21.5000	-0.30	[-0.70; 0.10]	3.5%	3.3%
Broders_and_Forbes	55	74.20	14.0900	55	69.40	19.2800	0.28	[-0.09; 0.66]	4.0%	3.3%
Broders_and_Forbes	57	67.40	16.6100	57	66.40	16.6100	0.06	[-0.31; 0.43]	4.2%	3.3%
Broders_and_Forbes	48	62.40	19.4000	48	54.20	21.4800	0.40	[-0.01; 0.80]	3.5%	3.3%
Carter	47	36.30	28.1100	37	36.62	35.0400	-0.01	[-0.44; 0.42]	3.0%	3.2%
Carter	19	61.32	28.2900	46	44.02	35.6800	0.51	[-0.04; 1.05]	1.9%	3.0%
Clement_and_Castleberry	25	85.90	12.3000	314	86.00	7.6000	-0.01	[-0.42; 0.39]	3.4%	3.3%
Fabianek_et_al	8	54.00	65.0000	8	98.00	3.0000	-0.90	[-1.95; 0.14]	0.5%	2.0%
Fabianek_et_al	40	24.00	75.0000	40	46.00	74.0000	-0.29	[-0.73; 0.15]	2.9%	3.2%
Herder_and_Jackson	52	55.20	19.4700	61	64.20	17.1800	-0.49	[-0.86; -0.11]	4.0%	3.3%
Johnson_et_al	16	71.95	13.9200	6	88.57	1.7400	-1.32	[-2.36; -0.29]	0.5%	2.0%
Johnson_et_al	34	85.37	18.0800	15	91.51	1.7400	-0.40	[-1.01; 0.22]	1.5%	2.9%
Jung_et_al	15	39.10	16.6500	52	70.00	11.5400	-2.38	[-3.09; -1.67]	1.1%	2.7%
Jung_et_al	11	31.60	19.5700	52	70.00	11.5400	-2.87	[-3.71; -2.04]	0.8%	2.4%
Lacki_and_Schwierjohann	57	91.40	0.9800	31	90.50	1.3900	0.78	[0.33; 1.24]	2.8%	3.2%
Menzel_et_al	12	91.70	8.3100	12	83.40	29.1000	0.37	[-0.43; 1.18]	0.9%	2.5%
Psyllakis_and_Brigham	19	64.87	13.7700	25	67.19	15.0000	-0.16	[-0.75; 0.44]	1.6%	2.9%
Psyllakis_and_Brigham	17	65.47	26.3500	21	79.40	11.0400	-0.70	[-1.36; -0.04]	1.3%	2.8%
Rabe_et_al	43	48.00	13.1100	58	53.00	22.8500	-0.26	[-0.65; 0.14]	3.6%	3.3%
Rabe_et_al	54	52.00	14.7000	54	57.00	14.7000	-0.34	[-0.72; 0.04]	3.9%	3.3%
Sasse_and_Pekins	47	83.00	9.6000	47	86.00	24.0000	-0.16	[-0.57; 0.24]	3.4%	3.3%
Vonhof_and_Gwilliam	46	42.00	28.1000	112	50.00	27.8000	-0.29	[-0.63; 0.06]	4.8%	3.4%
Vonhof_and_Gwilliam	46	38.00	23.2000	112	50.00	27.8000	-0.45	[-0.80; -0.10]	4.7%	3.4%
Vonhof_and_Gwilliam	20	35.00	26.9000	112	50.00	27.8000	-0.54	[-1.02; -0.06]	2.4%	3.1%
Weller_and_Zabel	23	78.50	12.4700	46	89.20	7.4600	-1.13	[-1.66; -0.59]	2.0%	3.0%
Lacki_et_al	16	47.00	20.0000	11	51.00	23.8800	-0.18	[-0.95; 0.59]	1.0%	2.5%
Lacki_et_al	35	65.00	26.0300	57	61.00	22.6500	0.17	[-0.26; 0.59]	3.2%	3.2%
**Fixed effect model**	**1282**			**2114**			**-0.23**	**[-0.31; -0.16]**	**100.0%**	**--**
**Random effects model**							**-0.34**	**[-0.56; -0.12]**	**--**	**100.0%**
**Prediction interval**								**[-1.56; 0.89]**		

Heterogeneity: $I^2 = 83\%$, $\tau^2 = 0.3483$, $p < 0.01$

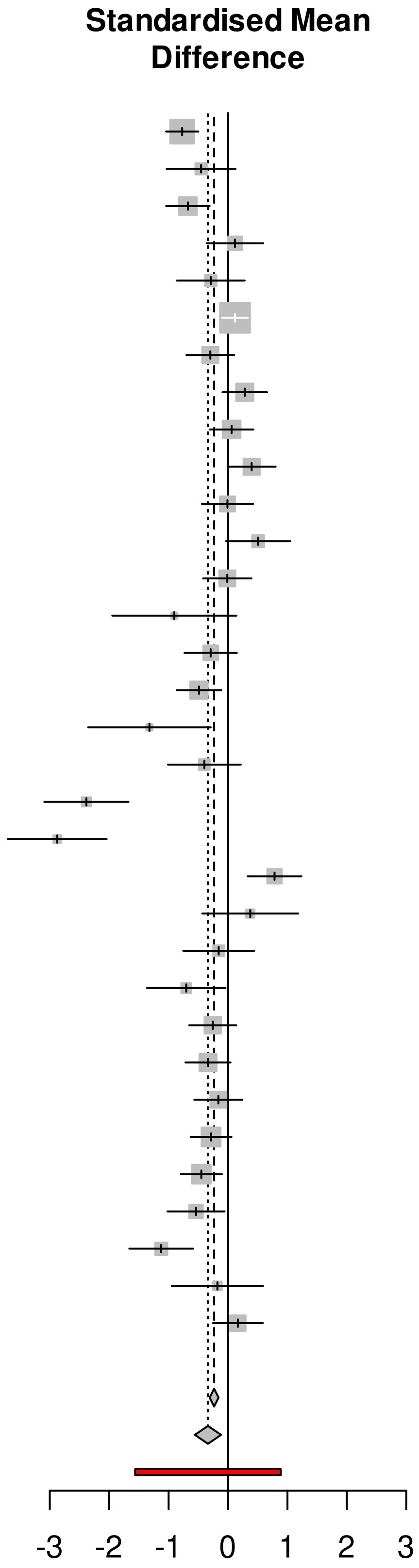

Distance to water

Study	Experimental Total	Experimental Mean	Experimental SD	Control Total	Control Mean	Control SD	SMD	95%-CI	Weight (fixed)	Weight (random)
Arnett_and_Hayes	105	1407.00	1332.1000	119	1829.00	1385.4100	-0.31	[-0.57; -0.05]	10.9%	6.4%
Arnett_and_Hayes	24	1205.00	925.9100	23	1470.00	1352.4200	-0.23	[-0.80; 0.35]	2.3%	3.6%
Arnett_and_Hayes	42	967.00	1056.3600	104	1883.00	1427.7300	-0.68	[-1.05; -0.32]	5.6%	5.3%
Arnett_and_Hayes	35	1582.00	1934.5600	33	1551.00	1338.4800	0.02	[-0.46; 0.49]	3.4%	4.3%
Arnett_and_Hayes	22	1480.00	1599.4300	26	1766.00	1126.8800	-0.21	[-0.78; 0.36]	2.3%	3.6%
Clement_and_Castleberry	25	626.80	468.5000	314	580.40	399.6000	0.11	[-0.29; 0.52]	4.6%	4.9%
Fabianek_et_al	40	779.38	798.5600	40	1341.80	662.5800	-0.76	[-1.21; -0.30]	3.7%	4.5%
Fleming_et_al	33	196.60	228.0600	139	256.00	365.4800	-0.17	[-0.55; 0.21]	5.2%	5.2%
Fleming_et_al	19	176.70	228.8400	144	244.00	363.6000	-0.19	[-0.67; 0.29]	3.3%	4.3%
Herder_and_Jackson	52	1032.00	413.2000	61	770.00	499.8600	0.56	[0.19; 0.94]	5.3%	5.2%
Jung_et_al	26	1800.00	1940.0000	52	1328.00	1388.0000	0.29	[-0.18; 0.77]	3.4%	4.3%
Lacki_and_Schwierjohann	57	127.00	100.4100	31	121.00	89.0800	0.06	[-0.38; 0.50]	4.0%	4.6%
Miles_et_al	111	333.30	246.5300	111	428.60	311.8600	-0.34	[-0.60; -0.07]	10.8%	6.4%
Miles_et_al	57	163.50	138.9200	57	191.90	145.7100	-0.20	[-0.57; 0.17]	5.6%	5.3%
Ormsbee_and_McComb	33	923.00	924.8700	66	1217.00	779.9100	-0.35	[-0.77; 0.07]	4.3%	4.8%
Psyllakis_and_Brigham	17	272.41	287.5900	21	301.43	205.6200	-0.12	[-0.76; 0.52]	1.8%	3.1%
Rabe_et_al	43	458.00	314.7600	58	701.00	350.3300	-0.72	[-1.13; -0.31]	4.6%	4.9%
Rabe_et_al	54	759.00	352.7300	54	855.00	558.4800	-0.20	[-0.58; 0.17]	5.3%	5.2%
Weller_and_Zabel	23	117.40	130.9300	46	149.70	130.2200	-0.24	[-0.75; 0.26]	3.0%	4.1%
Boland_et_al	62	101.00	141.7000	114	179.00	138.8000	-0.56	[-0.87; -0.24]	7.6%	5.8%
Boland_et_al	24	219.00	137.1700	44	182.00	132.6600	0.27	[-0.23; 0.77]	3.0%	4.1%
**Fixed effect model**	**904**			**1657**			**-0.23**	**[-0.31; -0.14]**	**100.0%**	**--**
**Random effects model**							**-0.20**	**[-0.35; -0.06]**	**--**	**100.0%**
**Prediction interval**								**[-0.77; 0.37]**		

Heterogeneity: $I^2 = 63\%$, $\tau^2 = 0.0677$, $p < 0.01$

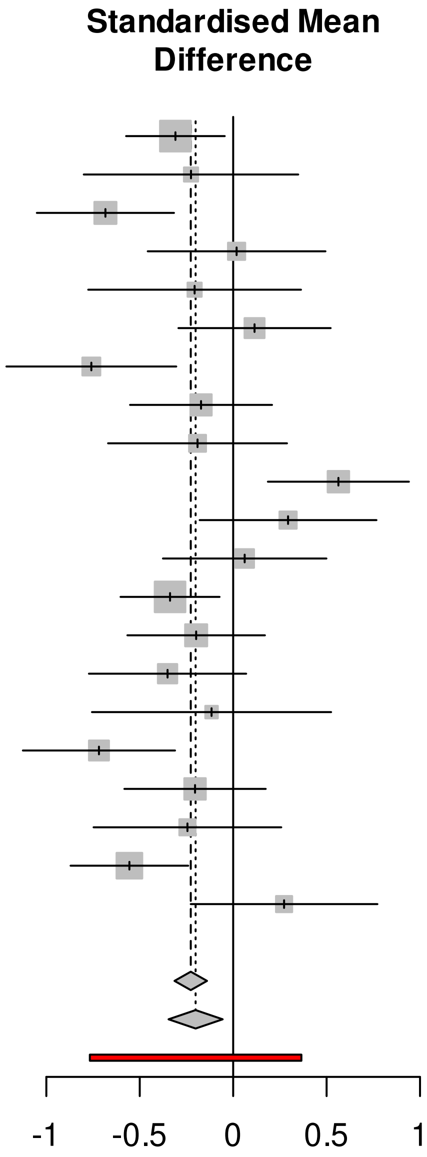

Tree density

Study	Experimental Total	Experimental Mean	Experimental SD	Control Total	Control Mean	Control SD	SMD	95%-CI	Weight (fixed)	Weight (random)
Baker_and_Lacki	164	291.70	224.1100	160	202.30	161.9100	0.46	[0.23; 0.68]	15.7%	5.4%
Baker_and_Lacki	28	249.70	245.5300	160	202.30	161.9100	0.27	[-0.13; 0.67]	4.7%	4.7%
Brigham_et_al	19	450.00	158.0000	38	490.00	227.0000	-0.19	[-0.74; 0.36]	2.5%	4.1%
Broders_and_Forbes	55	192.30	423.4600	55	123.10	61.5500	0.23	[-0.15; 0.60]	5.4%	4.8%
Broders_and_Forbes	57	217.60	372.9600	57	205.00	248.3900	0.04	[-0.33; 0.41]	5.7%	4.9%
Broders_and_Forbes	48	322.50	805.0600	48	238.90	474.5800	0.13	[-0.27; 0.53]	4.8%	4.7%
Clement_and_Castleberry	25	94.00	49.3000	314	150.30	140.9000	-0.41	[-0.82; 0.00]	4.6%	4.7%
Fabianek_et_al	8	105.00	59.2800	8	150.00	36.2500	-0.87	[-1.91; 0.17]	0.7%	2.4%
Fabianek_et_al	40	147.75	68.6700	40	124.00	81.8900	0.31	[-0.13; 0.75]	3.9%	4.6%
Jung_et_al	15	120.00	75.1400	52	198.10	53.3600	-1.31	[-1.93; -0.70]	2.0%	3.8%
Jung_et_al	11	104.60	69.6500	52	198.10	53.3600	-1.64	[-2.35; -0.92]	1.5%	3.4%
Lacki_and_Baker	6	590.00	279.2400	50	294.00	193.7500	1.44	[0.55; 2.33]	1.0%	2.8%
Menzel_et_al	12	74.00	40.5000	12	93.70	31.2000	-0.53	[-1.34; 0.29]	1.1%	3.0%
Parsons_et_al	6	8.00	4.2000	4	13.10	1.8000	-1.32	[-2.78; 0.15]	0.4%	1.5%
Rabe_et_al	43	111.42	73.6400	58	81.95	48.7400	0.48	[0.08; 0.88]	4.8%	4.7%
Rabe_et_al	54	104.67	74.1500	54	66.64	323.0400	0.16	[-0.22; 0.54]	5.4%	4.8%
Vonhof_and_Gwilliam	46	41.00	35.0000	112	43.00	37.1000	-0.05	[-0.40; 0.29]	6.5%	5.0%
Vonhof_and_Gwilliam	46	38.00	28.4000	112	43.00	37.1000	-0.14	[-0.49; 0.20]	6.5%	5.0%
Vonhof_and_Gwilliam	20	38.00	30.9000	112	43.00	37.1000	-0.14	[-0.61; 0.34]	3.4%	4.4%
Weller_and_Zabel	23	50.60	17.2600	46	57.70	26.4500	-0.29	[-0.80; 0.21]	3.0%	4.3%
Boland_et_al	62	36.10	14.2000	114	38.80	14.9500	-0.18	[-0.49; 0.13]	8.0%	5.1%
Boland_et_al	24	34.00	13.7200	44	32.70	14.5900	0.09	[-0.41; 0.59]	3.1%	4.3%
Lacki_et_al	16	36.30	20.0000	11	28.00	8.9500	0.49	[-0.29; 1.27]	1.3%	3.2%
Lacki_et_al	35	27.20	5.9200	57	25.20	7.5500	0.28	[-0.14; 0.71]	4.3%	4.6%
**Fixed effect model**	**863**			**1770**			**0.05**	**[-0.04; 0.14]**	**100.0%**	**--**
**Random effects model**							**-0.05**	**[-0.26; 0.15]**	**--**	**100.0%**
**Prediction interval**								**[-1.00; 0.89]**		

Heterogeneity: $I^2 = 75\%$, $\tau^2 = 0.1958$, $p < 0.01$

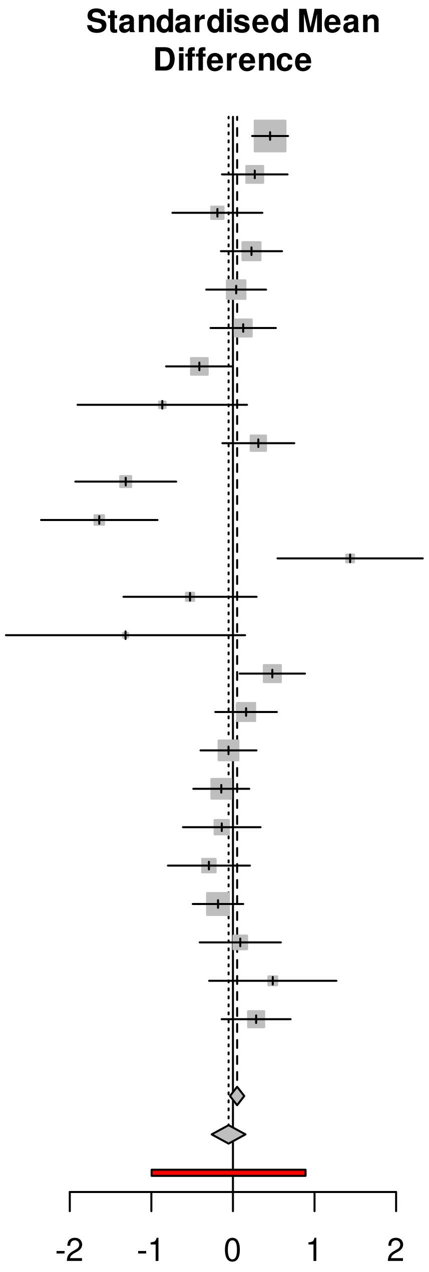

Slope

Study	Experimental Total	Experimental Mean	Experimental SD	Control Total	Control Mean	Control SD	SMD	95%-CI	Weight (fixed)	Weight (random)
Arnett_and_Hayes	105	32.40	20.4900	119	36.80	25.0900	-0.19	[-0.45; 0.07]	12.1%	7.4%
Arnett_and_Hayes	24	36.40	23.5200	23	42.80	23.5000	-0.27	[-0.84; 0.31]	2.5%	4.2%
Arnett_and_Hayes	42	35.60	22.6800	104	33.80	20.4000	0.08	[-0.27; 0.44]	6.5%	6.3%
Arnett_and_Hayes	35	42.30	28.4000	33	35.60	20.1100	0.27	[-0.21; 0.75]	3.7%	5.1%
Arnett_and_Hayes	22	34.10	18.7600	26	40.00	33.6500	-0.21	[-0.78; 0.36]	2.6%	4.3%
Baker_and_Lacki	164	26.70	25.6100	160	25.60	21.5000	0.05	[-0.17; 0.26]	17.6%	7.9%
Baker_and_Lacki	28	31.20	25.9300	160	25.60	21.5000	0.25	[-0.15; 0.65]	5.2%	5.8%
Broders_and_Forbes	55	6.60	3.7100	55	7.40	4.4500	-0.19	[-0.57; 0.18]	6.0%	6.1%
Broders_and_Forbes	57	4.60	4.5300	57	5.40	10.5700	-0.10	[-0.47; 0.27]	6.2%	6.2%
Broders_and_Forbes	48	4.60	4.1600	48	4.90	4.8500	-0.07	[-0.47; 0.33]	5.2%	5.8%
Fleming_et_al	33	3.32	5.0100	139	9.29	16.4600	-0.40	[-0.78; -0.01]	5.7%	6.0%
Fleming_et_al	19	2.62	3.0400	144	9.28	14.6600	-0.48	[-0.96; 0.00]	3.6%	5.0%
Herder_and_Jackson	52	4.00	5.0500	61	6.10	5.4700	-0.39	[-0.77; -0.02]	6.0%	6.1%
Johnson_et_al	16	29.13	11.6800	6	43.50	12.5400	-1.16	[-2.17; -0.15]	0.8%	2.0%
Lacki_and_Baker	6	52.30	15.2800	50	34.30	20.5800	0.88	[0.02; 1.74]	1.1%	2.5%
Menzel_et_al	12	23.27	7.8600	12	25.30	6.0600	-0.28	[-1.08; 0.53]	1.3%	2.8%
Rabe_et_al	43	14.05	11.4800	58	10.51	13.3300	0.28	[-0.12; 0.68]	5.3%	5.9%
Rabe_et_al	53	23.09	12.8600	54	12.28	12.8600	0.83	[0.44; 1.23]	5.3%	5.9%
Weller_and_Zabel	23	29.70	24.9400	46	35.70	14.9200	-0.32	[-0.82; 0.19]	3.3%	4.8%
**Fixed effect model**	**837**			**1355**			**-0.04**	**[-0.13; 0.05]**	**100.0%**	**--**
**Random effects model**							**-0.05**	**[-0.21; 0.11]**	**--**	**100.0%**
**Prediction interval**								**[-0.64; 0.55]**		

Heterogeneity: $I^2 = 64\%$, $\tau^2 = 0.0726$, $p < 0.01$

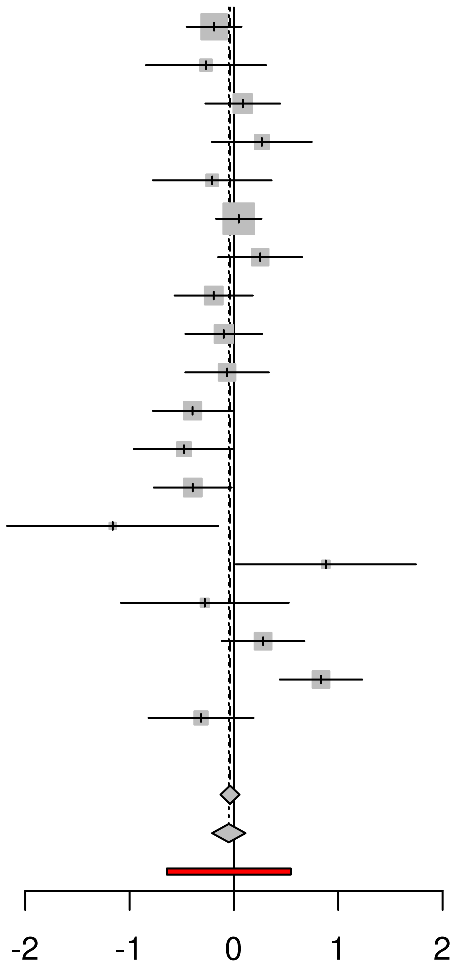

Bark remaining

Study	Experimental Total	Experimental Mean	Experimental SD	Control Total	Control Mean	Control SD	Standardised Mean Difference	SMD	95%-CI	Weight (fixed)	Weight (random)
Baker_and_Lacki	164	64.60	34.9000	160	47.40	54.6000		0.38	[0.16; 0.60]	15.3%	4.4%
Baker_and_Lacki	28	40.50	21.9000	160	47.40	54.6000		-0.13	[-0.54; 0.27]	4.6%	4.2%
Brigham_et_al	19	75.00	29.6000	38	86.00	27.3000		-0.39	[-0.94; 0.17]	2.4%	4.0%
Broders_and_Forbes	55	78.50	30.4100	55	73.70	33.3700		0.15	[-0.22; 0.52]	5.3%	4.2%
Broders_and_Forbes	57	74.40	31.7100	57	59.00	37.7500		0.44	[0.07; 0.81]	5.3%	4.2%
Broders_and_Forbes	48	72.10	31.1800	48	69.00	34.6400		0.09	[-0.31; 0.49]	4.6%	4.2%
Callahan_et_al	11	81.10	16.9100	14	76.20	16.4600		0.28	[-0.51; 1.08]	1.2%	3.6%
Callahan_et_al	20	69.10	18.3400	5	69.80	23.0300		-0.04	[-1.02; 0.94]	0.8%	3.3%
Carter	47	47.02	26.6700	37	55.00	35.3400		-0.26	[-0.69; 0.18]	3.9%	4.2%
Carter	19	79.21	30.9000	46	63.84	38.3900		0.42	[-0.12; 0.96]	2.5%	4.0%
Fabianek_et_al	8	93.13	15.8000	8	70.63	34.8900		0.79	[-0.24; 1.81]	0.7%	3.2%
Fabianek_et_al	40	0.74	26.7300	40	54.50	39.6900		-1.57	[-2.08; -1.07]	2.9%	4.1%
Johnson_et_al	34	76.79	30.9600	15	46.67	31.7600		0.95	[0.31; 1.59]	1.8%	3.8%
Jung_et_al	15	61.00	19.7500	52	77.00	32.4500		-0.52	[-1.11; 0.06]	2.2%	3.9%
Jung_et_al	11	63.60	27.2000	52	77.00	32.4500		-0.42	[-1.07; 0.24]	1.7%	3.8%
Kniowski_and_Gehrt	56	68.80	33.0800	1438	98.70	9.8600		-2.58	[-2.86; -2.30]	9.2%	4.3%
Psyllakis_and_Brigham	19	78.11	17.5200	25	85.76	16.0000		-0.45	[-1.06; 0.15]	2.0%	3.9%
Sasse_and_Pekins	47	78.00	38.3900	47	66.00	13.0300		0.42	[0.01; 0.82]	4.4%	4.2%
Vonhof_and_Gwilliam	46	93.00	17.6000	112	72.00	26.2000		0.87	[0.51; 1.23]	5.8%	4.2%
Vonhof_and_Gwilliam	46	90.00	23.3000	112	72.00	26.2000		0.71	[0.35; 1.06]	5.9%	4.2%
Vonhof_and_Gwilliam	20	70.00	28.0000	112	72.00	26.2000		-0.08	[-0.55; 0.40]	3.3%	4.1%
Boland_et_al	62	63.00	29.1000	114	90.60	30.9600		-0.91	[-1.23; -0.58]	7.0%	4.3%
Boland_et_al	24	37.40	29.3900	44	78.20	29.1900		-1.38	[-1.93; -0.83]	2.4%	4.0%
Lacki_et_al	16	62.00	36.0000	11	40.00	33.8300		0.61	[-0.18; 1.39]	1.2%	3.6%
Lacki_et_al	35	83.00	26.0300	57	46.00	36.9900		1.10	[0.65; 1.55]	3.6%	4.1%
**Fixed effect model**	**947**			**2859**				**-0.15**	**[-0.24; -0.06]**	**100.0%**	**--**
**Random effects model**								**-0.07**	**[-0.41; 0.27]**	**--**	**100.0%**
**Prediction interval**									**[-1.82; 1.67]**		

Heterogeneity: $I^2 = 95\%$, $\tau^2 = 0.6795$, $p < 0.01$

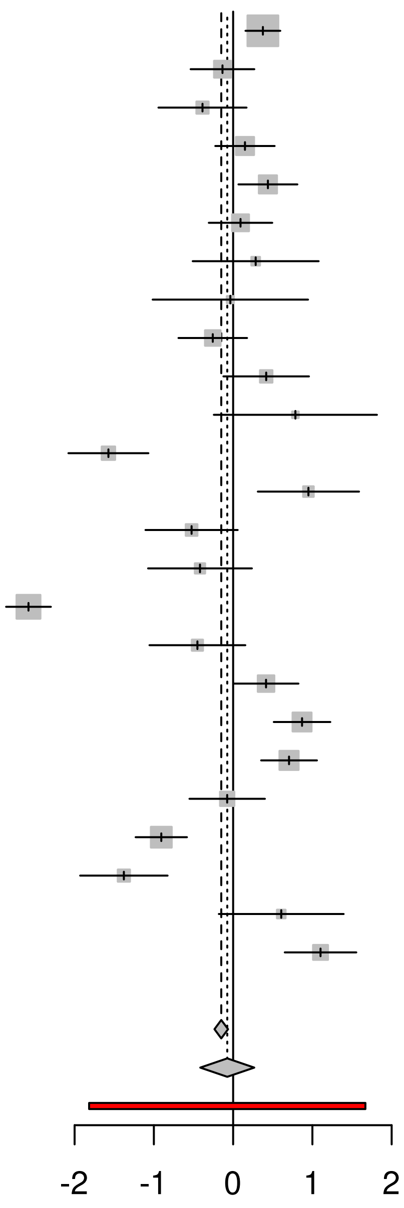

Table 1. Summary of the random effects meta-analysis of roost selection by North American bats, with heterogeneity indices and publication biases for each characteristic.

Characteristic	*K*	SMD	95%-CI		*Z*	*P*	τ^{2}*	I^2 (%)	95% CI (%)		t	df	*P*
Tree diameter	63	0.70	0.56	0.84	9.89	<0.0001	0.23	0.75	0.69	0.81	1.85	61	0.07
Tree height	47	0.54	0.36	0.71	6.05	<0.0001	0.29	0.85	0.80	0.88	-0.66	45	0.51
Number of snags	34	0.46	0.31	0.60	6.28	<0.0001	0.11	0.69	0.55	0.78	-0.59	32	0.56
Elevation	19	-0.34	-0.49	-0.19	-4.50	<0.0001	0.05	0.55	0.24	0.73	-0.07	17	0.94
Canopy closure	33	-0.34	-0.56	-0.12	-3.00	<0.0001	0.35	0.83	0.77	0.87	-1.84	31	0.08
Distance to water	21	-0.20	-0.35	-0.06	-2.72	0.01	0.07	0.63	0.40	0.77	1.20	19	0.24
Tree density	24	-0.05	-0.26	0.15	-0.50	0.61	0.20	0.75	0.63	0.83	-2.23	22	0.05
Slope	19	-0.05	-0.21	0.11	-0.60	0.55	0.07	0.64	0.41	0.78	-0.44	17	0.66
Bark remaining on trunks	25	-0.07	-0.41	0.27	-0.43	0.67	0.68	0.95	0.94	0.96	0.51	23	0.61

K = number of datasets and SMD = standardized mean difference; τ2 and I2 indices indicate the severity of between-studies heterogeneity; t-tests are for funnel-plot asymmetry, with associated degrees-of-freedom and P-values. All values are rounded upward to two decimal places.

* Estimated by maximum likelihood

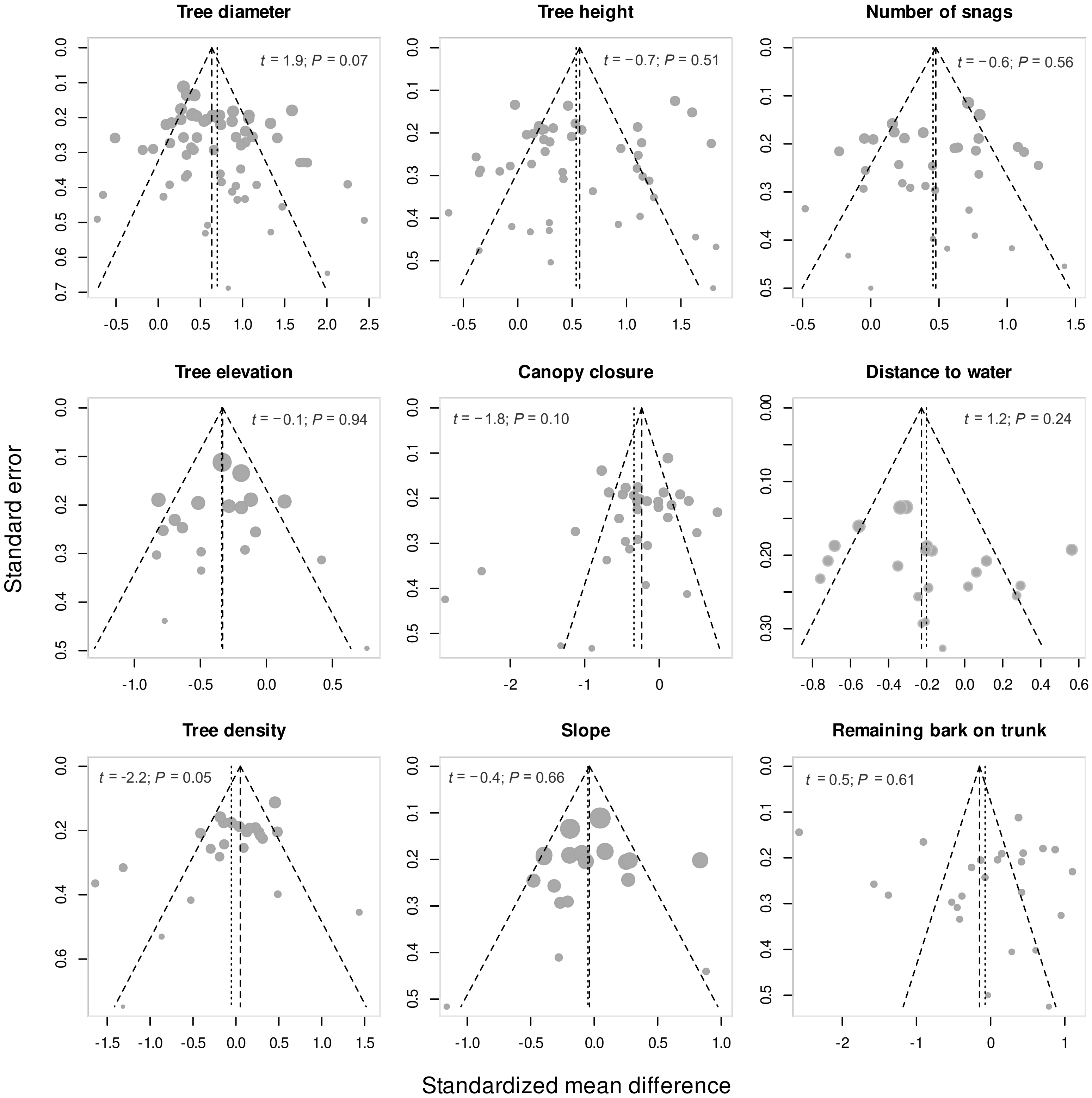

Fig 3. Funnel plots showing publication bias for each of the nine characteristics that were included in our quantitative meta-analysis. For each dataset, the effect size on the horizontal axis (standardized mean difference) is plotted against its standard error on the vertical axis. Dotted lines define the 95% CI limits around the mean effect size (vertical dotted line). The size of the circle varies according to the assigned random weight (inverse variance of the standardized mean differences) of each dataset. Funnel plot asymmetry t-test results and associated P-values are shown in each plot. In the absence of publication bias, studies should follow a symmetric funnel shape. Deviation from this shape may indicate publication bias.

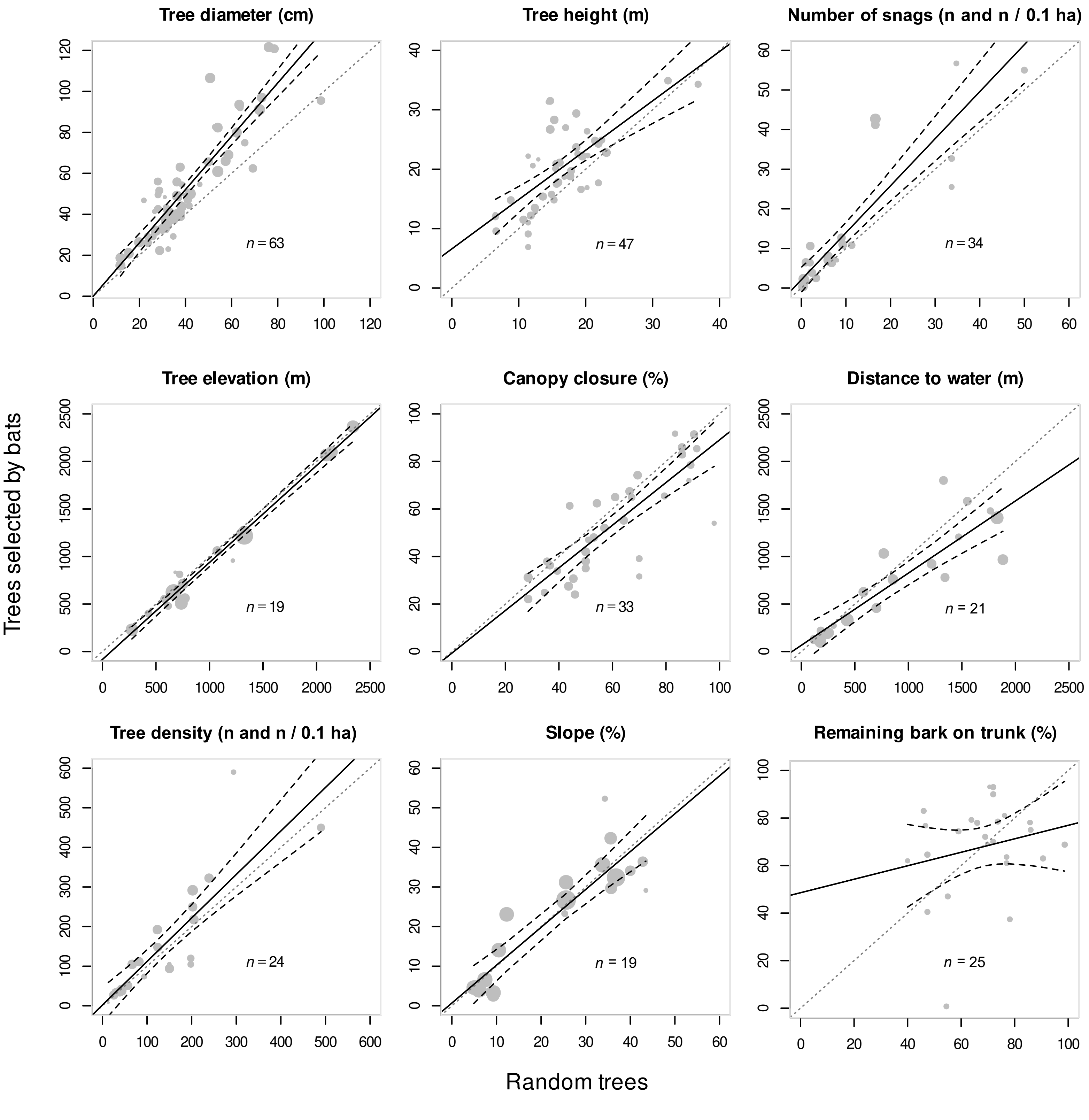

Fig 4. L'Abbé plots of the tree characteristics selected by bats (experimental groups) against the random tree characteristics (control group) with the 95% CI (black dashed lines) for each dataset, and for each characteristic (tree diameter, tree height, snag density, bark remaining on trunks, distance to water, canopy closure, elevation, slope, and stand density). The size of the circle varies according to the assigned random weight (inverse variance of the standardized mean differences) of each dataset. The diagonal (x = y) grey dotted line is the equality line (1:1) between both means (i.e., the zero effect line, for which the mean difference = 0). Above the x = y line, the experimental group mean is higher than the control group mean. Below the x = y line, the experimental group mean is lower than the control group mean. Tau-squared (τ^2) and Higgins' I^2 heterogeneity indices are shown in each plot. Higgins' I^2 index is expressed in percentage and is used to interpret the severity of heterogeneity.
