# Supplementary material for: Expression of Concern: Exploring Regional Variation in Roost Selection by Bats: Evidence from a Meta-Analysis
Source: PLoS One. 2024 Dec 18;19(12):e0316243. doi: 10.1371/journal.pone.0316243 (PMC11654921; doi:10.1371/journal.pone.0316243)
Supplement: S2 File — These files provide clarifications regarding sources, extraction and conversion of data; and descriptions of errors and their corrections provided by the corresponding author. Readers should also refer to the Expression of Concern notice section on dataset errors. (ZIP) [file pone.0316243.s002.zip › S1-S9 Table Correction Reports/S1_Table_correction_report.docx]

# S1_Table.docx (tree diameter)

I have made a complete review of all references used in the data table, and listed below are the errors I have found and all the points raised regarding this dataset:

- The data used for ([Arnett & Hayes 2009](#_ENREF_1)) were obtained from his PhD thesis (https://ir.library.oregonstate.edu/concern/graduate_thesis_or_dissertations/ff365816w).
- The data used for ([Carter 2003](#_ENREF_6))were obtained from his PhD thesis (reference 63).
- Mean selected trees reported in ([Clement & Castleberry 2013](#_ENREF_7)) is 93.5 cm and not 95.5 cm. The wrong number 93.5 was used in the analyses
- Number of selected trees reported in the S1_Table is 1. However, the number used in the analyses and reported for ([Brigham *et al.* 1997](#_ENREF_4)) is 19. The error is only in the S1_Table.
- The mean and SD values in ([Foster & Kurta 1999](#_ENREF_10)) for random trees were derived from the results given in Table 2.
- The data used for ([Broders & Forbes 2004](#_ENREF_5)) were obtained from his PhD thesis (reference 62).
- The data used for ([Fabianek *et al.* 2015](#_ENREF_9)) were obtained from his PhD thesis (https://library-archives.canada.ca/eng/services/services-libraries/theses/Pages/item.aspx?idNumber=1273433671).
- The data used for ([Grindal 1999](#_ENREF_11)) was provided by Matina Kalcounis-Rueppell in a database format and used in [Kalcounis-Rueppell, Psyllakis and Brigham (2005)](#_ENREF_13)
- The data used for ([Ormsbee & McComb 1998](#_ENREF_14)) were obtained from the published paper for the roost tree values, complemented from her thesis version (https://andrewsforest.oregonstate.edu/publications/2273) for the random tree values. However, the values were wrongly reported for both roost and random trees. The mean and SE reported for random trees in Ormsbee’s thesis (p. 53) have a mean of 73 cm with a SE of 3.54 while the values reported in the S1_Table are 66 cm with a SE of 2. The mean and SE reported for roost trees in Ormsbee’s thesis (p. 53) have a mean of 97 cm with a SE of 6.63, while the values reported in the S1_Table are 100 cm with a SE of 6. The values of 100 cm with a SE of 6 are also reported in ([Ormsbee & McComb 1998](#_ENREF_14)): *the mean dbh for all snags and trees used as day roosts was 100 ± 6 cm (range = 34-172 cm).*
- The data used for ([Perry & Thill 2007](#_ENREF_15)) were obtained from the published paper. Although only the selected tree values are reported in numbers in the published paper, I could use Fig. 1 to derivate the values reported for the available trees. Although the available trees were reported in percentage in Fig. 1, it is mentioned in the published paper that each roost tree was matched with its random location where they defined the availab**l**e trees. I thus assumed that Perry & Thill matched an equal number of available trees compared to the total number of roost trees used by males and females, which leads to n = 82 available trees. I approximated the number of available trees per snag class with a cross product from 100 % to n = 82. I then used the median value for each snag class and derived mean and SD from the number (n) of available trees obtained in each snag class using these median values. I obtained a mean DBH for available trees of 11.8 cm with a SD of 6.3. It was the best approximation I could obtain from this source. I preferred to rely on an approximate value for the available tree diameter than discard the results of this paper.
- The data used in ([Perry & Thill 2008](#_ENREF_16)) were obtained from the published paper. Although the number of random trees is not mentioned in number in the published paper, it is mentioned that they selected a random tree and surrounding 0.10-ha plot for comparison with each roost tree. Because all roosts were in snags, they selected only snags for random trees. I thus assumed that Perry & Thill matched an equal number of random trees compared to the total number of roost trees used by males and females, which leads to n = 34 trees.
- The data used in ([Hein 2009](#_ENREF_12)) were obtained from his PhD thesis.
- The number of selected trees reported in ([Boland *et al.* 2009](#_ENREF_3)) is 62 and not 60. The wrong number of 60 was reported and used in the analyses. Consequently, the new estimation of SD is 34.6, instead of the previous 34.1 reported with a n = 60.
- The data used in ([Barclay, Faure & Farr 1988](#_ENREF_2)) were obtained from the published paper. Mean and SE for selected and random trees are reported in meter and values were taken from the circumference of the trees. I converted the circumference values to diameter values and reported the results in cm.
- All the other values reported in the S1_Table that were not mentioned in the points raised above, were obtained from published papers. The mean values were all reported in cm along with corresponding SE or SD. All the values reported were carefully reviewed and no additional error was reported in the S1_Table.

To conclude, four errors were reported in the S1_Table due to clerical mistakes and were corrected as mentioned above.

I have re-run the meta-analysis on tree diameter (DBH) from the corrected S1_Table and obtained the following results:

SMD 95%-CI %W(fixed) %W(random)

Arnett_and_Hayes 1.0755 [ 0.6960; 1.4550] 2.6 1.9

Arnett_and_Hayes 0.9234 [ 0.4217; 1.4251] 1.5 1.7

Arnett_and_Hayes -0.1837 [-0.7570; 0.3895] 1.1 1.6

Arnett_and_Hayes 0.4285 [ 0.1630; 0.6939] 5.3 2.1

Arnett_and_Hayes -0.0596 [-0.6275; 0.5083] 1.1 1.6

Baker_and_Lacki 0.2996 [ 0.0806; 0.5186] 7.7 2.1

Baker_and_Lacki 1.3319 [ 0.9080; 1.7558] 2.1 1.8

Brigham_et_al 1.7697 [ 1.1242; 2.4152] 0.9 1.5

Broders_and_Forbes 0.2677 [-0.1342; 0.6697] 2.3 1.9

Broders_and_Forbes 0.3979 [ 0.0204; 0.7755] 2.6 1.9

Broders_and_Forbes 0.4073 [ 0.0362; 0.7783] 2.7 1.9

Callahan_et_al 0.5591 [-0.4817; 1.5998] 0.3 1.0

Callahan_et_al 1.0292 [ 0.1806; 1.8779] 0.5 1.2

Callahan_et_al 0.5854 [-0.4104; 1.5812] 0.4 1.0

Carter 0.1402 [-0.3949; 0.6753] 1.3 1.7

Carter 0.0940 [-0.3370; 0.5250] 2.0 1.8

Clement_and_Castleberry 0.8763 [ 0.4636; 1.2890] 2.2 1.9

Cryan_et_al 0.8779 [ 0.0706; 1.6852] 0.6 1.3

Cryan_et_al 0.6604 [ 0.0870; 1.2338] 1.1 1.6

Cryan_et_al 0.9212 [ 0.1457; 1.6966] 0.6 1.3

Cryan_et_al 0.9384 [ 0.0852; 1.7916] 0.5 1.2

Fabianek_et_al 2.0063 [ 0.7408; 3.2718] 0.2 0.8

Fabianek_et_al 1.0345 [ 0.5665; 1.5026] 1.7 1.8

Fleming_et_al 0.3466 [-0.3648; 1.0580] 0.7 1.4

Fleming_et_al 1.1669 [ 0.3972; 1.9365] 0.6 1.3

Foster_and_Kurta 1.1179 [ 0.6181; 1.6176] 1.5 1.7

Foster_and_Kurta 0.3004 [-0.2027; 0.8036] 1.5 1.7

Grindal_et_al 0.7363 [ 0.0289; 1.4437] 0.7 1.4

Grindal_et_al 0.3207 [-0.4060; 1.0474] 0.7 1.4

Herder_and_Jackson 0.6452 [ 0.2655; 1.0249] 2.6 1.9

Johnson_et_al 1.3357 [ 0.3013; 2.3702] 0.3 1.0

Jung_et_al 1.6798 [ 1.0349; 2.3248] 0.9 1.5

Jung_et_al 2.2465 [ 1.4802; 3.0128] 0.6 1.3

Kniowski_and_Gehrt 0.3924 [-0.1671; 0.9519] 1.2 1.6

Lacki_and_Baker 1.4708 [ 0.5781; 2.3634] 0.5 1.2

Menzel_et_al -0.6544 [-1.4798; 0.1710] 0.5 1.2

Miles_et_al 0.3431 [ 0.0780; 0.6081] 5.3 2.1

Miles_et_al 0.7263 [ 0.3468; 1.1057] 2.6 1.9

Ormsbee_and_McComb 0.7411 [ 0.3101; 1.1720] 2.0 1.8

Parsons_et_al 0.8306 [-0.5182; 2.1794] 0.2 0.7

Perry_and_Till 0.4539 [ 0.0714; 0.8363] 2.5 1.9

Perry_and_Till 1.0736 [ 0.6777; 1.4695] 2.4 1.9

Psyllakis_and_Brigham 0.3361 [-0.2649; 0.9372] 1.0 1.6

Psyllakis_and_Brigham 0.9787 [ 0.2983; 1.6590] 0.8 1.5

Rabe_et_al 0.5746 [ 0.1720; 0.9772] 2.3 1.9

Rabe_et_al 0.6852 [ 0.2967; 1.0738] 2.5 1.9

Sasse_and_Pekins 0.5435 [ 0.1315; 0.9555] 2.2 1.9

Vonhof_and_Gwilliam 0.8880 [ 0.5307; 1.2453] 2.9 1.9

Vonhof_and_Gwilliam 0.2712 [-0.0734; 0.6157] 3.1 2.0

Vonhof_and_Gwilliam 1.4127 [ 0.9065; 1.9190] 1.4 1.7

Weller_and_Zabel 1.0325 [ 0.5015; 1.5636] 1.3 1.7

Perry_and_Till 0.7514 [-0.0020; 1.5048] 0.7 1.3

Perry_and_Till 0.9810 [ 0.4331; 1.5289] 1.2 1.7

Boland_et_al 1.5863 [ 1.2345; 1.9381] 3.0 2.0

Boland_et_al 0.4600 [-0.0437; 0.9637] 1.5 1.7

Hein -0.5113 [-1.0178; -0.0049] 1.4 1.7

Hein 0.4211 [-0.1514; 0.9937] 1.1 1.6

Hein 0.0627 [-0.7733; 0.8987] 0.5 1.2

Hein -0.7225 [-1.6842; 0.2393] 0.4 1.1

Lacki_et_al 0.1357 [-0.6330; 0.9044] 0.6 1.3

Lacki_et_al 0.1576 [-0.2640; 0.5791] 2.1 1.9

Barclay_et_al 1.7200 [ 1.0765; 2.3636] 0.9 1.5

Barclay_et_al 2.4434 [ 1.4752; 3.4115] 0.4 1.1

Number of studies combined: k = 63

SMD 95%-CI z p-value

Random effects model 0.7003 [0.5615; 0.8391] 9.89 < 0.0001

Quantifying heterogeneity:

tau^2 = 0.2246; H = 2.02 [1.79; 2.28]; I^2 = 75.4% [68.7%; 80.7%]

Test of heterogeneity:

Q d.f. p-value

252.00 62 < 0.0001

From this new results, I can see that the reported SMD for the random effet model varied from the previously reported 0.71 in Table 1 ([Fabianek, Simard & Desrochers 2015](#_ENREF_8)) to 0.70 here (see results above). The reported 95%CI also varied from previous 0.57; 086 to 0.56; 0.84. The Z value varied from previous 9.87 to 9.89 with similar p-value. The r^2^ value varied from previous 0.24 to 0.23. The I^2^ varied from previous 0.76 to 75 % with previous 95%CI from previous 0.70; 0,81 to 0.69; 0.81.

The publication bias reported for tree diameter with funnel plots with the new corrected data give similar results than previously reported. Similarly, I have performed a new l’Abbé plot for tree diameter, and the resulting graph is similar. Despite these 3 minor modifications in the values, the overal results, their rank, their interpretation and the conlusions remain unchanged.

I have also performed a Moran’s I test for spatial autocorrelation and the values given by the test were slighty different than previously reported (because this test uses the new SMD values which are slightly different from those previously reported). Despite these minor modifications in the values, the overal results, their interpretation and the conlusion remain similar (i.e., no spatial correlation). Same remark with the meta-regression models and the delta AICc model ranking. Despite these minor modifications in the values, the overal results, their interpretation, and the conlusion remain similar:

# K AICc deltaAIC rel.LL weights

13 3 112.89 0.00 1.00 0.36

10 5 113.98 1.09 0.58 0.21

12 4 115.05 2.16 0.34 0.12

7 6 116.41 3.52 0.17 0.06

11 5 116.69 3.80 0.15 0.05

16 7 117.26 4.37 0.11 0.04

15 9 117.45 4.56 0.10 0.04

9 8 117.51 4.62 0.10 0.04

8 10 118.43 5.54 0.06 0.02

17 4 118.51 5.62 0.06 0.02

14 3 118.75 5.86 0.05 0.02

6 9 120.23 7.34 0.03 0.01

5 11 121.29 8.40 0.01 0.01

1 11 125.63 12.74 0.00 0.00

4 7 126.79 13.90 0.00 0.00

3 8 128.44 15.55 0.00 0.00

2 10 129.28 16.39 0.00 0.00

You can see, here the two best models are still Temperature (# 13), and Temperature + sex (#10), followed by Temperature + elevation (#12).

The main conclusion is that I have corrected for four clerical mistakes located in the published S1_Table, including one only reported in the S1_Table, but not included in the dataset used for the analyses. From these modifications, I have found slight different values in SMD and corresponding statistics. These slight differences were reported in other analyses but without consequences for the manuscript quality: despite these minor modifications in the values, the overal results, their ranking, their interpretation and the conlusions remain all similar for the “tree diameter” variable.

## References

Arnett, E.B. & Hayes, J.P. (2009) Use of conifer snags as roosts by female bats in western Oregon. *Journal of Wildlife Management,* **73,** 214-225.

Barclay, R.M.R., Faure, P.A. & Farr, D.R. (1988) Roosting behavior and roost selection by migrating silver-haired bats (*Lasionycteris noctivagans*). *Journal of Mammalogy,* **69,** 821-825.

Boland, J.L., Hayes, J.P., Smith, W.P. & Huso, M.M. (2009) Selection of day-roosts by Keen's myotis (*Myotis keenii*) at multiple spatial scales. *Journal of Mammalogy,* **90,** 222-234.

Brigham, R.M., Vonhof, M.J., Barclay, R.M.R. & Gwilliam, J.C. (1997) Roosting behavior and roost-site preferences of forest-dwelling California bats (*Myotis californicus*). *Journal of Mammalogy,* **78,** 1231-1239.

Broders, H.G. & Forbes, G.J. (2004) Interspecific and intersexual variation in roost-site selection of northern long-eared and little brown bats in the Greater Fundy National Park ecosystem. *Journal of Wildlife Management,* **68,** 602-610.

Carter, T.C. (2003) Summer habitat use of roost trees by the endangered Indiana bat *(Myotis sodalis*) in the Shawnee National Forest of southern Illinois. Ph.D., Carbondale University.

Clement, M.J. & Castleberry, S.B. (2013) Southeastern myotis (*Myotis austroriparius*) roost selection in cypress-gum swamps. *Acta Chiropterologica,* **15,** 133-141.

Fabianek, F., Simard, M.A. & Desrochers, A. (2015) Exploring regional variation in roost selection by bats: evidence from a meta-analysis. *PLoS ONE,* **10,** e0139126.

Fabianek, F., Simard, M.A., Racine B., E. & Desrochers, A. (2015) Selection of roosting habitat by male *Myotis* bats in a boreal forest. *Canadian Journal of Zoology***,** 539-546.

Foster, R.W. & Kurta, A. (1999) Roosting ecology of the northern bat (*Myotis septentrionalis*) and comparisons with the endangered Indiana bat (*Myotis sodalis*). *Journal of Mammalogy,* **80,** 659-672.

Grindal, S.D. (1999) Habitat use by bats, *Myotis* spp., in western Newfoundland. *Canadian Field-Naturalist,* **113,** 258-263.

Hein, C.D. (2009) Bat activity and roost-site selection on an intensively managed pine landscape with forested corridors in the lower coastal plain of South Carolina. Doctor of Philosophy, The University of Georgia.

Kalcounis-Rueppell, M.C., Psyllakis, J.M. & Brigham, R.M. (2005) Tree roost selection by bats: an empirical synthesis using meta-analysis. *Wildlife Society Bulletin,* **33,** 1123-1132.

Ormsbee, P.C. & McComb, W.C. (1998) Selection of day roosts by female long-legged myotis in the central Oregon Cascade range. *Journal of Wildlife Management,* **62,** 596-603.

Perry, R.W. & Thill, R.E. (2007) Roost selection by male and female northern long-eared bats in a pine-dominated landscape. *Forest Ecology and Management,* **247,** 220-226.

Perry, R.W. & Thill, R.E. (2008) Roost selection by big brown bats in forests of Arkansas: importance of pine snags and open forest habitats to males. *Southeastern Naturalist,* **7,** 607-618.
