# Supplementary material for: Expression of Concern: Exploring Regional Variation in Roost Selection by Bats: Evidence from a Meta-Analysis
Source: PLoS One. 2024 Dec 18;19(12):e0316243. doi: 10.1371/journal.pone.0316243 (PMC11654921; doi:10.1371/journal.pone.0316243)
Supplement: S2 File — These files provide clarifications regarding sources, extraction and conversion of data; and descriptions of errors and their corrections provided by the corresponding author. Readers should also refer to the Expression of Concern notice section on dataset errors. (ZIP) [file pone.0316243.s002.zip › S1-S9 Table Correction Reports/S2_Table_correction_report.docx]

# S2_Table.docx (tree Height)

I have made a complete review of all references used in the data table, and listed below are the errors I have found and all the points raised regarding this dataset:

- The data used for ([Arnett & Hayes 2009](#_ENREF_1)) were obtained from his PhD thesis (https://ir.library.oregonstate.edu/concern/graduate_thesis_or_dissertations/ff365816w).
- The data used for ([Carter 2003](#_ENREF_3)) were obtained from his PhD thesis (reference 63).
- The data used for ([Broders & Forbes 2004](#_ENREF_2)) were obtained from his PhD thesis (reference 62). Tree height is not given in the data table, but I could calculate it from the data table because canopy height and tree height relative to canopy is given. I added the tree height to canopy value to the canopy height value to find the tree height value.
- The data used for ([Fabianek *et al.* 2015](#_ENREF_5)) were obtained from his PhD thesis (thesis (https://library-archives.canada.ca/eng/services/services-libraries/theses/Pages/item.aspx?idNumber=1273433671).
- The data used for ([Ormsbee & McComb 1998](#_ENREF_7)) were obtained from her thesis version (https://andrewsforest.oregonstate.edu/publications/2273).
- The tree height value of selected trees reported in ([Psyllakis & Brigham 2006](#_ENREF_9)) is 26.4 and not 16.4. The wrong number of 26.4 was reported and used in the analyses.
- The data used in ([Perry & Thill 2008](#_ENREF_8)) were obtained from the published paper. Although the number of random trees is not mentioned in number in the published paper, it is mentioned that they selected a random tree and surrounding 0.10-ha plot for comparison with each roost tree. Because all roosts were in snags, they selected only snags for random trees. I thus assumed that Perry & Thill matched an equal number of random trees compared to the total number of roost trees used by males and females, which leads to n = 34 trees.
- The data used in ([Hein 2009](#_ENREF_6)) were obtained from his PhD thesis.
- All the other values reported in the S2_Table that were not mentioned in the points raised above, were obtained from published papers. The mean values were all reported in m along with corresponding SE or SD. All the values reported were carefully reviewed and no additional error was reported in the S2_Table.

To conclude, one error was reported in the S2_Table due to a clerical mistake and was corrected as mentionned above.

I have re-run the meta-analysis on tree height from the corrected S2_Table and obtained the following results:

SMD 95%-CI %W(fixed) %W(random)

Arnett_and_Hayes -0.0261 [-0.2886; 0.2363] 6.5 2.6

Arnett_and_Hayes -0.3549 [-0.9317; 0.2219] 1.3 2.1

Arnett_and_Hayes 0.1955 [-0.1636; 0.5545] 3.5 2.5

Arnett_and_Hayes 0.2514 [-0.2262; 0.7289] 2.0 2.3

Arnett_and_Hayes -0.1653 [-0.7341; 0.4035] 1.4 2.1

Baker_and_Lacki 1.4462 [ 1.2013; 1.6911] 7.4 2.6

Baker_and_Lacki 1.7791 [ 1.3384; 2.2198] 2.3 2.3

Brigham_et_al 1.1488 [ 0.5563; 1.7413] 1.3 2.1

Broders_and_Forbes 0.2421 [-0.1330; 0.6173] 3.2 2.4

Broders_and_Forbes 0.1907 [-0.1773; 0.5587] 3.3 2.4

Broders_and_Forbes 0.0818 [-0.3184; 0.4821] 2.8 2.4

Carter 0.2945 [-0.1387; 0.7277] 2.4 2.3

Carter 0.1273 [-0.4077; 0.6623] 1.6 2.2

Clement_and_Castleberry 0.4957 [ 0.0867; 0.9048] 2.7 2.4

Cryan_et_al -0.3412 [-0.9031; 0.2207] 1.4 2.1

Cryan_et_al 1.6350 [ 0.7638; 2.5061] 0.6 1.6

Cryan_et_al -0.6357 [-1.3953; 0.1240] 0.8 1.8

Cryan_et_al -0.0551 [-0.8779; 0.7677] 0.7 1.7

Fabianek_et_al 0.3029 [-0.6845; 1.2903] 0.5 1.5

Fabianek_et_al 0.9482 [ 0.4847; 1.4116] 2.1 2.3

Herder_and_Jackson 0.5880 [ 0.2099; 0.9660] 3.1 2.4

Johnson_et_al 1.7963 [ 0.6901; 2.9024] 0.4 1.3

Jung_et_al 1.2094 [ 0.5974; 1.8214] 1.2 2.0

Jung_et_al 1.2516 [ 0.5631; 1.9401] 0.9 1.9

Lacki_and_Baker 1.8233 [ 0.9069; 2.7396] 0.5 1.6

Lacki_et_al 1.6038 [ 1.3062; 1.9014] 5.0 2.5

Menzel_et_al 0.2903 [-0.5149; 1.0954] 0.7 1.7

Miles_et_al 0.4624 [ 0.1958; 0.7291] 6.3 2.6

Miles_et_al 0.3256 [-0.0441; 0.6952] 3.3 2.4

Psyllakis_and_Brigham 0.4201 [-0.1834; 1.0237] 1.2 2.1

Psyllakis_and_Brigham 0.6906 [ 0.0304; 1.3507] 1.0 2.0

Rabe_et_al 0.1425 [-0.2524; 0.5374] 2.9 2.4

Rabe_et_al 0.2230 [-0.1554; 0.6015] 3.1 2.4

Sasse_and_Pekins 1.1393 [ 0.7021; 1.5765] 2.3 2.3

Vonhof_and_Gwilliam 0.5303 [ 0.1820; 0.8786] 3.7 2.5

Vonhof_and_Gwilliam 1.1026 [ 0.7380; 1.4673] 3.4 2.4

Vonhof_and_Gwilliam 1.1071 [ 0.6124; 1.6019] 1.8 2.2

Perry_and_Till 1.1246 [ 0.3488; 1.9005] 0.7 1.8

Perry_and_Till 1.0969 [ 0.5413; 1.6524] 1.4 2.1

Hein -0.3828 [-0.8854; 0.1199] 1.8 2.2

Hein 0.4125 [-0.1598; 0.9848] 1.4 2.1

Hein 0.2900 [-0.5511; 1.1310] 0.6 1.7

Hein -0.3539 [-1.2870; 0.5793] 0.5 1.5

Lacki_et_al 0.9251 [ 0.1124; 1.7379] 0.7 1.7

Lacki_et_al 0.2405 [-0.1819; 0.6629] 2.5 2.4

Barclay_et_al -0.0688 [-0.6130; 0.4754] 1.5 2.2

Barclay_et_al 0.1157 [-0.7314; 0.9628] 0.6 1.7

Number of studies combined: k = 47

SMD 95%-CI z p-value

Fixed effect model 0.5696 [0.5028; 0.6364] 16.72 < 0.0001

Random effects model 0.5371 [0.3631; 0.7110] 6.05 < 0.0001

Quantifying heterogeneity:

tau^2 = 0.2872; H = 2.56 [2.26; 2.89]; I^2 = 84.7% [80.5%; 88.0%]

Test of heterogeneity:

Q d.f. p-value

300.92 46 < 0.0001

From this new results, I can see that the reported SMD for the random effect model varied from the previously reported 0.51 in Table 1 ([Fabianek, Simard & Desrochers 2015](#_ENREF_4)) to 0.54 here (see results above). The reported 95%CI also varied from previous 0.34; 0.69 to 0.36; 0.71. The Z value varied from previous 5.67 to 6.05 with similar p-value. The r^2^ value varied from previous 0.30 to 0.29. The I^2^ with 95%CI did not varied from previous 0.85 with 0.81; 0.88.

The publication bias reported for tree height with funnel plots with the new corrected data give similar results than previously reported. Similarly, I have performed a new l’Abbé plot for tree height, and the resulting graph is similar. Despite these minor modifications in the values, the overal results, their rank, their interpretation and the conlusions remain unchanged.

The main conclusion is that I have corrected for one clerical mistake located in the published S2_Table. From this modification, I have found slight different values in SMD and corresponding statistics, but without consequences for the manuscript quality: despite these minor modifications in the values, the overal results, their ranking, their interpretation and the conlusions remain all similar for the “tree height” variable.

## References

Arnett, E.B. & Hayes, J.P. (2009) Use of conifer snags as roosts by female bats in western Oregon. *Journal of Wildlife Management,* **73,** 214-225.

Broders, H.G. & Forbes, G.J. (2004) Interspecific and intersexual variation in roost-site selection of northern long-eared and little brown bats in the Greater Fundy National Park ecosystem. *Journal of Wildlife Management,* **68,** 602-610.

Carter, T.C. (2003) Summer habitat use of roost trees by the endangered Indiana bat *(Myotis sodalis*) in the Shawnee National Forest of southern Illinois. Ph.D., Carbondale University.

Fabianek, F., Simard, M.A. & Desrochers, A. (2015) Exploring regional variation in roost selection by bats: evidence from a meta-analysis. *PLoS ONE,* **10,** e0139126.

Fabianek, F., Simard, M.A., Racine B., E. & Desrochers, A. (2015) Selection of roosting habitat by male *Myotis* bats in a boreal forest. *Canadian Journal of Zoology***,** 539-546.

Hein, C.D. (2009) Bat activity and roost-site selection on an intensively managed pine landscape with forested corridors in the lower coastal plain of South Carolina. Doctor of Philosophy, The University of Georgia.

Ormsbee, P.C. & McComb, W.C. (1998) Selection of day roosts by female long-legged myotis in the central Oregon Cascade range. *Journal of Wildlife Management,* **62,** 596-603.

Perry, R.W. & Thill, R.E. (2008) Roost selection by big brown bats in forests of Arkansas: importance of pine snags and open forest habitats to males. *Southeastern Naturalist,* **7,** 607-618.

Psyllakis, J.M. & Brigham, R.M. (2006) Characteristics of diurnal roosts used by female *Myotis* bats in sub-boreal forests. *Forest Ecology and Management,* **223,** 93-102.
