# Supplementary material for: Expression of Concern: Exploring Regional Variation in Roost Selection by Bats: Evidence from a Meta-Analysis
Source: PLoS One. 2024 Dec 18;19(12):e0316243. doi: 10.1371/journal.pone.0316243 (PMC11654921; doi:10.1371/journal.pone.0316243)
Supplement: S2 File — These files provide clarifications regarding sources, extraction and conversion of data; and descriptions of errors and their corrections provided by the corresponding author. Readers should also refer to the Expression of Concern notice section on dataset errors. (ZIP) [file pone.0316243.s002.zip › S1-S9 Table Correction Reports/S3_Table_correction_report.docx]

# S3_Table.docx (number of snags or snag density)

I have made a complete review of all references used in the data table, and listed below are the errors I have found and all the points raised regarding this dataset:

- The data used for ([Arnett & Hayes 2009](#_ENREF_1)) were obtained from his PhD thesis (https://ir.library.oregonstate.edu/concern/graduate_thesis_or_dissertations/ff365816w).
- The data used for ([Carter 2003](#_ENREF_3)) were obtained from his PhD thesis (reference 63).
- The data used for ([Broders & Forbes 2004](#_ENREF_2)) were obtained from his PhD thesis (reference 62).
- The data used for ([Fabianek *et al.* 2015](#_ENREF_5)) were obtained from his PhD thesis thesis (https://library-archives.canada.ca/eng/services/services-libraries/theses/Pages/item.aspx?idNumber=1273433671).
- The data used for ([Ormsbee & McComb 1998](#_ENREF_10)) were obtained from her thesis version (https://andrewsforest.oregonstate.edu/publications/2273).
- The data used in ([Hein 2009](#_ENREF_6)) were obtained from his PhD thesis.
- All the other values reported in the S3_Table that were not mentioned in the points raised above, were obtained from published papers.
- The values in (basal area) m^2^/20 m diameter plot (for snags with DBH > 25 cm) reported in ([Lacki & Baker 2007](#_ENREF_7)) were actually converted in stems / 20 m diameter plot. I could not retrace the material I have used to converted this data, but I could perform the conversion again using a conversion table made by D. Coder (Warnell School of Forestry & Natural Resources, University of Georgia) untitled “*trees per acre: by diameter & besal area.*”. I could find somewhat similar values except for the derived mean and SE of stems in the selected stands. The new converted values for random plots are a mean of 8 stems / 20 m diameter plot for snags with DBH > 25 cm with a SD of 6.2, instead of a previous converted mean of 8.8 with a SD of 6.8. The new converted values for selected plots are a mean of 7 stems / 20 m diameter plot for snags with DBH > 25 cm with a SD of 3.1, instead of a previous converted mean of 17.2 with a SD of 9.3.
- Similarly, the values in (basal area) m^2^/0.03 ha plot (for snags) reported in ([Menzel *et al.* 2002](#_ENREF_9)), were converted in stems per / 0.03 ha plot with the information given in Table 2. Again, I could not retrace the way I converted this data, but I could perform a conversion again using with the information given in Table 2. The number of overstory trees in 0.03 ha plot is reported along with the percent of overstory composed of snags in 0.03 ha plot. I deduced the number of snags by applying a cross product between the number of overstory trees and the percent of overstory composed of snags. I could find somewhat similar values for the mean and SE for selected and random plots: the new converted values for random plots are a mean of 1.1 stems for snags with a SD of 1.7, instead of a previous converted mean of 1.1 stems with a SD of 1. The new converted values for selected plots are a mean of 1.8 stems for snags with a SD of 0.13, instead of a previous converted mean of 1.4 with a SD of 0.9.
- The values from ([Lacki *et al.* 2009](#_ENREF_8)) were all converted from 1 ha to 0.1 ha by dividing the reported values by a ratio of 10.

All values were reported in “number of snags”or “available snags per ha” or “snags per ha”, “snags per 0.1 ha” or “snags per 0.03 ha” or “snags per plot of 20 m diameter with DBH > 25 cm” or “the mean proportion of trees in early to advances stages of decay”, etc. with corresponding SE or SD. As I have already mentioned, all these papers used different methodologies to report this snag variable at the stand scale. Whenever possible, I tried to convert and report the values to a number of snags per 0.1 ha. However, I kept the original values when it was not possible to perform any change. I did not discard the studies that only used “the number of snags” without reporting the value to a given surface. I also did not discard the studies that reported “the number of snags” to a different surface values than 1 ha. I only did the conversion (i.e., dividing by ten) to 1 ha, when the number of snags was reported on 10 ha and I tried somehow to convert the basal area of snags reported in m^2^ in number of stems. Despite keeping studies with differences in reported measurements to increase our sample size of candidate studies, I also wanted to make sure that these differences did not unbalance too much the results by excluding “*studies with an effect size greater than 4 times the mean group standard deviation to meet criteria of effect size normality and variance homogeneity.”*

I think it was necessary and justified to keep these studies in the analyses and **for two main reasons**: First, instead of relying on a fixed effect model to estimate the SMD from this studies, I used a random effect model that partly account for these uncertainties and differences in the studies’ approaches. (See Borenstein et al. (2010) paper for further information: Borenstein M, Hedges LV, Higgins JPT, Rothstein HR. A basic introduction to fixed-effect and random-effects models for meta-analysis. Research Synthesis Methods. 2010; 1(2):97–111. doi: 10.1002/jrsm.12 PMID: 26061376):

Secondly, the idea of using heterogeneity indices was precisely to show the heterogeneity in the results and try to interpret these sources of heterogeneity in the discussion. In the discussion, I made it clear that the heterogeneity was also attributed to a difference in methodology and approaches in the studies I have reviewed: *We expected a high degree of heterogeneity because the studies that we included in our meta-analysis were conducted in various habitats, had included numerous bat species, and attempted to answer different questions (…). It is likely that the differences in results among studies were also influenced by measurement methods.”*

Of course, if the results are not reported with the same surface units that will obviously raise the heterogeneity among studies and influence the effect sizes (SMD). It would have been more accurate to discard this effect in order to obtain a SMD + heterogeneity indices that would only be linked to a difference in selected roosting habitats by bats VS random samples in the studies. **Now, the most important point to consider here is that excluding the influence of the different measurements methods from the estimated studies’ effect sizes was not the purpose of my meta-analysis.** The purpose was to rank the most common variables to use only the one with the highest value of effect size (SMD) which was **tree diameter**. In other words, the point was to discard the other variables, and use the one that was actually linked with thermal regulation (which was the main hypothesis of the paper) and that explained the most roost selection by cavity roosting bats at the continental scale.

I also recognize that writing “stems / 0.1 ha” in the S3 Table was misleading, because I have kept the values for a few publications (see above) that used a different surface unit (greater or lower than 0.1 ha) to report the number of stems in their publications.

To conclude, I have reconverted basal areas to stems per unit surface for two publications with different approaches that I have previously used. I have re-run the meta-analysis on the number of snags with the corrected S3_Table and obtained the following results:

SMD 95%-CI %W(fixed) %W(random)

Arnett_and_Hayes 0.7985 [ 0.5257; 1.0714] 7.7 4.0

Arnett_and_Hayes 0.4718 [-0.1088; 1.0523] 1.7 2.6

Arnett_and_Hayes 0.7911 [ 0.4211; 1.1610] 4.2 3.6

Arnett_and_Hayes 0.4530 [-0.0290; 0.9350] 2.5 3.1

Arnett_and_Hayes 0.2895 [-0.2815; 0.8605] 1.8 2.7

Baker_and_Lacki 0.7135 [ 0.4888; 0.9382] 11.3 4.2

Baker_and_Lacki 0.6389 [ 0.2321; 1.0457] 3.4 3.4

Brigham_et_al 0.2314 [-0.3210; 0.7839] 1.9 2.8

Broders_and_Forbes 0.0178 [-0.3559; 0.3916] 4.1 3.6

Broders_and_Forbes 0.2470 [-0.1216; 0.6156] 4.2 3.6

Broders_and_Forbes 0.6137 [ 0.2039; 1.0234] 3.4 3.4

Cryan_et_al 0.4006 [-0.1629; 0.9641] 1.8 2.7

Cryan_et_al 1.0339 [ 0.2163; 1.8515] 0.9 1.8

Cryan_et_al 0.7629 [-0.0031; 1.5289] 1.0 2.0

Cryan_et_al 1.4196 [ 0.5286; 2.3107] 0.7 1.7

Fabianek_et_al 0.0000 [-0.9800; 0.9800] 0.6 1.5

Fabianek_et_al 1.2287 [ 0.7489; 1.7085] 2.5 3.1

Herder_and_Jackson -0.0474 [-0.4174; 0.3226] 4.2 3.6

Jung_et_al -0.0533 [-0.6278; 0.5212] 1.7 2.7

Jung_et_al -0.4807 [-1.1369; 0.1755] 1.3 2.4

Lacki_and_Baker -0.1649 [-1.0123; 0.6825] 0.8 1.8

Menzel_et_al 0.5606 [-0.2581; 1.3793] 0.9 1.8

Psyllakis_and_Brigham 0.7201 [ 0.0582; 1.3820] 1.3 2.3

Rabe_et_al 1.1233 [ 0.6984; 1.5482] 3.2 3.3

Rabe_et_al 1.0787 [ 0.6740; 1.4833] 3.5 3.4

Sasse_and_Pekins 0.7703 [ 0.3507; 1.1900] 3.2 3.4

Vonhof_and_Gwilliam 0.1717 [-0.1720; 0.5155] 4.8 3.7

Vonhof_and_Gwilliam 0.3842 [ 0.0383; 0.7301] 4.8 3.7

Vonhof_and_Gwilliam 0.2058 [-0.2707; 0.6822] 2.5 3.1

Weller_and_Zabel -0.0396 [-0.5402; 0.4609] 2.3 3.0

Boland_et_al 0.1591 [-0.1540; 0.4721] 5.8 3.9

Boland_et_al 0.7920 [ 0.2761; 1.3079] 2.1 2.9

Lacki_et_al 0.4558 [-0.3231; 1.2346] 0.9 2.0

Lacki_et_al -0.2315 [-0.6538; 0.1907] 3.2 3.3

Number of studies combined: k = 34

SMD 95%-CI z p-value

Fixed effect model 0.4770 [0.4015; 0.5526] 12.38 < 0.0001

Random effects model 0.4563 [0.3140; 0.5987] 6.28 < 0.0001

Quantifying heterogeneity:

tau^2 = 0.1114; H = 1.78 [1.49; 2.13]; I^2 = 68.6% [55.2%; 77.9%]

Test of heterogeneity:

Q d.f. p-value

104.95 33 < 0.0001

From these new results, I can see that the reported SMD for the random effect model varied from the previously reported 0.47 in Table 1 ([Fabianek, Simard & Desrochers 2015](#_ENREF_4)) to 0.46 here (see results above). The reported 95%CI also varied from previous 0.33; 0.62 to 0.31; 0.60. The Z value varied from previous 6.49 to 6.28 with similar p-value. The varied from previous 0.30 to 0.11. The r^2^ and I^2^  with 95%CI values did not varied from previous values.

The publication bias reported for tree height with funnel plots with the new corrected data give similar results than previously reported. Similarly, I have performed a new l’Abbé plot for tree diameter, and the resulting graph is similar. Despite these minor modifications in the values, the overall results, their rank, their interpretation and the conclusions remain unchanged.

The main conclusion is that I have made the conversion again from m^2^ to stems per unit surface for two studies using different approaches than used in the published S3_Table. From these modifications, I have found slight different values in SMD and corresponding statistics, but without consequences for the manuscript quality: despite these minor modifications in the values, the overall results, their ranking, their interpretation and the conclusions remain all similar for the “number of snags” variable.

## References

Arnett, E.B. & Hayes, J.P. (2009) Use of conifer snags as roosts by female bats in western Oregon. *Journal of Wildlife Management,* **73,** 214-225.

Broders, H.G. & Forbes, G.J. (2004) Interspecific and intersexual variation in roost-site selection of northern long-eared and little brown bats in the Greater Fundy National Park ecosystem. *Journal of Wildlife Management,* **68,** 602-610.

Carter, T.C. (2003) Summer habitat use of roost trees by the endangered Indiana bat *(Myotis sodalis*) in the Shawnee National Forest of southern Illinois. Ph.D., Carbondale University.

Fabianek, F., Simard, M.A. & Desrochers, A. (2015) Exploring regional variation in roost selection by bats: evidence from a meta-analysis. *PLoS ONE,* **10,** e0139126.

Fabianek, F., Simard, M.A., Racine B., E. & Desrochers, A. (2015) Selection of roosting habitat by male *Myotis* bats in a boreal forest. *Canadian Journal of Zoology***,** 539-546.

Hein, C.D. (2009) Bat activity and roost-site selection on an intensively managed pine landscape with forested corridors in the lower coastal plain of South Carolina. Doctor of Philosophy, The University of Georgia.

Lacki, M.J. & Baker, M.D. (2007) Day roosts of female fringed myotis (*Myotis thysanodes*) in xeric forests of the Pacific Northwest. *Journal of Mammalogy,* **88,** 967-973.

Lacki, M.J., Cox, D.R., Dodd, L.E. & Dickinson, M.B. (2009) Response of Northern bats (*Myotis septentrionalis*) to prescribed fires in eastern Kentucky forests. *Journal of Mammalogy,* **90,** 1165-1175.

Menzel, M.A., Owen, S.F., Ford, W.M., Edwards, J.W., Wood, P.B., Chapman, B.R. & Miller, K.V. (2002) Roost tree selection by northern long-eared bat (*Myotis septentrionalis*) maternity colonies in an industrial forest of the central Appalachian mountains. *Forest Ecology and Management,* **155,** 107-114.

Ormsbee, P.C. & McComb, W.C. (1998) Selection of day roosts by female long-legged myotis in the central Oregon Cascade range. *Journal of Wildlife Management,* **62,** 596-603.
