# Supplementary material for: Expression of Concern: Exploring Regional Variation in Roost Selection by Bats: Evidence from a Meta-Analysis
Source: PLoS One. 2024 Dec 18;19(12):e0316243. doi: 10.1371/journal.pone.0316243 (PMC11654921; doi:10.1371/journal.pone.0316243)
Supplement: S2 File — These files provide clarifications regarding sources, extraction and conversion of data; and descriptions of errors and their corrections provided by the corresponding author. Readers should also refer to the Expression of Concern notice section on dataset errors. (ZIP) [file pone.0316243.s002.zip › S1-S9 Table Correction Reports/S4_Table_correction_report.docx]

# S4_Table.docx (elevation)

I have made a complete review of all references used in the data table, and listed below are the errors I have found and all the points raised regarding this dataset:

- The data used for (Arnett & Hayes 2009) were obtained from his PhD thesis (https://ir.library.oregonstate.edu/concern/graduate_thesis_or_dissertations/ff365816w).
- All the other values reported in the S4_Table that were not mentioned in the points raised above, were obtained from published papers.
- The number of selected and random trees reported in Fleming et al. (2013) for live trees were 33 and 139 and not 8 and 157, respectively. Similarly, the number of selected and random trees reported in Fleming et al. (2013) for snags were 19 and 144 and not 7 and 147, respectively. Because of these corrected *n* values, the converted SD (from reported SE in the paper) is 95 instead of 45 for the random snags; and 30.5 instead of 63.6 for the selected live trees. Other SD values remained unaffected.

Because of this clerical error, I rerun the SMD analysis with the new *n* and SD values in the S4_Table:

SMD 95%-CI %W(fixed) %W(random)

Arnett_and_Hayes -0.1912 [-0.4543; 0.0718] 13.2 8.3

Arnett_and_Hayes -0.1619 [-0.7348; 0.4110] 2.8 4.2

Arnett_and_Hayes -0.8172 [-1.1879; -0.4465] 6.6 6.6

Arnett_and_Hayes -0.7819 [-1.2763; -0.2874] 3.7 5.0

Arnett_and_Hayes -0.8315 [-1.4252; -0.2378] 2.6 4.0

Baker_and_Lacki -0.3353 [-0.5546; -0.1159] 19.0 9.0

Baker_and_Lacki -0.1893 [-0.5912; 0.2127] 5.6 6.1

Fabianek_et_al -0.6949 [-1.1468; -0.2429] 4.5 5.5

Fleming_et_al -0.5164 [-0.8999; -0.1329] 6.2 6.4

Fleming_et_al -0.6358 [-1.1193; -0.1523] 3.9 5.1

Herder_and_Jackson -0.1176 [-0.4879; 0.2527] 6.7 6.6

Johnson_et_al 0.7614 [-0.2092; 1.7319] 1.0 1.9

Johnson_et_al 0.4179 [-0.1957; 1.0315] 2.4 3.8

Jung_et_al -0.4942 [-1.0751; 0.0867] 2.7 4.1

Jung_et_al -0.4938 [-1.1504; 0.1627] 2.1 3.5

Lacki_and_Baker -0.7703 [-1.6300; 0.0893] 1.2 2.3

Rabe_et_al -0.2826 [-0.6790; 0.1139] 5.8 6.2

Rabe_et_al 0.1376 [-0.2401; 0.5153] 6.4 6.5

Weller_and_Zabel -0.0817 [-0.5825; 0.4190] 3.6 4.9

Number of studies combined: k = 19

SMD 95%-CI z p-value

Fixed effect model -0.3309 [-0.4264; -0.2354] -6.79 < 0.0001

Random effects model -0.3392 [-0.4869; -0.1914] -4.50 < 0.0001

Quantifying heterogeneity:

tau^2 = 0.0506; H = 1.48 [1.15; 1.92]; I^2 = 54.6% [23.9%; 73.0%]

From these new results, I can see that the reported SMD for the random effects model varied from the previously reported -0.35 in Table 1 (Fabianek, Simard & Desrochers 2015) to -0.34 here (see results above). The reported 95%CI also varied from previous -0.51; -0.18 to -0.49; -0.19. The Z value varied from previous -4.11 to -4.50 with similar p-value. The r^2^ value varied from previous 0.07 to 0.05. The I^2^ varied from previous 58 to 55 % with previous 95%CI from previous 0.31; 0.75 to 0.24; 0.73.

The publication bias reported for elevation with funnel plots with the new corrected data give similar results than previously reported. Similarly, I have performed a new l’Abbé plot for this variable, and the resulting graph is similar. Despite these minor modifications in the values due to a clerical mistake, the overall results, their interpretation, their ranking in Table 1 and the conclusions remain unchanged.

## References

Arnett, E.B. & Hayes, J.P. (2009) Use of conifer snags as roosts by female bats in western Oregon. *Journal of Wildlife Management,* **73,** 214-225.

Fabianek, F., Simard, M.A. & Desrochers, A. (2015) Exploring regional variation in roost selection by bats: evidence from a meta-analysis. *PLoS ONE,* **10,** e0139126.

Fleming HL, Jones JC, Belant JL, Richardson DM. Multi-scale roost site selection by Rafinesque's big-eared bat (Corynorhinus rafinesquii) and southeastern myotis (Myotis austroriparius) in Mississippi. American Midland Naturalist. 2013;**169**(1):43–55.
