# Supplementary material for: Expression of Concern: Exploring Regional Variation in Roost Selection by Bats: Evidence from a Meta-Analysis
Source: PLoS One. 2024 Dec 18;19(12):e0316243. doi: 10.1371/journal.pone.0316243 (PMC11654921; doi:10.1371/journal.pone.0316243)
Supplement: S2 File — These files provide clarifications regarding sources, extraction and conversion of data; and descriptions of errors and their corrections provided by the corresponding author. Readers should also refer to the Expression of Concern notice section on dataset errors. (ZIP) [file pone.0316243.s002.zip › S1-S9 Table Correction Reports/S5_Table_correction_report.docx]

# S5_Table.docx (Canopy closure)

I have made a complete review of all references used in the data table, and listed below are the errors I have found and all the points raised regarding this dataset:

- The data used for (Arnett & Hayes 2009) were obtained from his PhD thesis (https://ir.library.oregonstate.edu/concern/graduate_thesis_or_dissertations/ff365816w).
- The data used for (Carter 2003) were obtained from his PhD thesis (reference 63).
- The data used for (Broders & Forbes 2004) were obtained from his PhD thesis (reference 62). Three height is not given in the data table, but I could calculate it from the data table because canopy height and tree height relative to canopy is given. I added the tree height to canopy value to the canopy height value to find the tree height value.
- The data used for (Fabianek *et al.* 2015) were obtained from his PhD thesis (https://library-archives.canada.ca/eng/services/services-libraries/theses/Pages/item.aspx?idNumber=1273433671).
- All the other values reported in the S5_Table that were not mentioned in the points raised above, were obtained from published papers.
- When authors used Canopy opening in % instead of Canopy closure in %, canopy opening (GAP) data was converted in canopy closure by subtracting 100 % to the canopy opening values. This conversion was performed with (Fabianek *et al.* 2015) and (Johnson *et al.* 2008) datasets.
- The mean random trees reported in Arnett & Hayes (2009) for MYVO was 39.4 and not 29.4 .
- The mean selected trees reported in Fabianek *et al.* (2015) after conversion in canopy closure was 24 % and not 44 %.
- The mean selected trees reported in Lacki *et al.* (2009) for prescribed fires was 45 % and not 47 %.
- Because of these clerical errors, I rerun the SMD analysis with the corrected mean values in the S5_Table:

SMD 95%-CI %W(fixed) %W(random)

Arnett_and_Hayes -0.7718 [-1.0440; -0.4997] 7.6 3.5

Arnett_and_Hayes -0.4535 [-1.0333; 0.1264] 1.7 2.9

Arnett_and_Hayes -0.6757 [-1.0425; -0.3088] 4.2 3.3

Arnett_and_Hayes 0.1162 [-0.3598; 0.5922] 2.5 3.1

Arnett_and_Hayes -0.2916 [-0.8626; 0.2794] 1.7 2.9

Baker_and_Lacki 0.1172 [-0.1007; 0.3352] 11.9 3.5

Baker_and_Lacki -0.2982 [-0.7008; 0.1045] 3.5 3.3

Broders_and_Forbes 0.2823 [-0.0934; 0.6580] 4.0 3.3

Broders_and_Forbes 0.0598 [-0.3074; 0.4270] 4.2 3.3

Broders_and_Forbes 0.3975 [-0.0067; 0.8016] 3.5 3.3

Carter -0.0101 [-0.4409; 0.4206] 3.0 3.2

Carter 0.5067 [-0.0353; 1.0487] 1.9 3.0

Clement_and_Castleberry -0.0124 [-0.4197; 0.3949] 3.4 3.3

Fabianek_et_al -0.9041 [-1.9484; 0.1402] 0.5 2.0

Fabianek_et_al -0.2924 [-0.7332; 0.1483] 2.9 3.2

Herder_and_Jackson -0.4893 [-0.8649; -0.1137] 4.0 3.3

Johnson_et_al -1.3229 [-2.3556; -0.2902] 0.5 2.0

Johnson_et_al -0.3980 [-1.0111; 0.2150] 1.5 2.9

Jung_et_al -2.3835 [-3.0927; -1.6743] 1.1 2.7

Jung_et_al -2.8741 [-3.7058; -2.0424] 0.8 2.4

Lacki_and_Schwierjohann 0.7826 [ 0.3295; 1.2357] 2.8 3.2

Menzel_et_al 0.3745 [-0.4340; 1.1830] 0.9 2.5

Psyllakis_and_Brigham -0.1573 [-0.7548; 0.4402] 1.6 2.9

Psyllakis_and_Brigham -0.7030 [-1.3639; -0.0422] 1.3 2.8

Rabe_et_al -0.2567 [-0.6528; 0.1393] 3.6 3.3

Rabe_et_al -0.3377 [-0.7177; 0.0423] 3.9 3.3

Sasse_and_Pekins -0.1628 [-0.5678; 0.2422] 3.4 3.3

Vonhof_and_Gwilliam -0.2855 [-0.6302; 0.0592] 4.8 3.4

Vonhof_and_Gwilliam -0.4497 [-0.7966; -0.1028] 4.7 3.4

Vonhof_and_Gwilliam -0.5390 [-1.0193; -0.0586] 2.4 3.1

Weller_and_Zabel -1.1250 [-1.6616; -0.5885] 2.0 3.0

Lacki_et_al -0.1793 [-0.9487; 0.5901] 1.0 2.5

Lacki_et_al 0.1654 [-0.2562; 0.5870] 3.2 3.2

Number of studies combined: k = 33

SMD 95%-CI z p-value

Fixed effect model -0.2343 [-0.3094; -0.1591] -6.11 < 0.0001

Random effects model -0.3378 [-0.5589; -0.1168] -3.00 0.0027

Quantifying heterogeneity:

tau^2 = 0.3483 [0.2292; 0.8274];  I^2 = 83.0% [77.0%; 87.4%]

From these new results, I can see that the reported SMD for the random effects model varied from the previously reported -0.32 in Table 1 (Fabianek, Simard & Desrochers 2015) to -0.34 here (see results above). The reported 95%CI also varied from previous -0.54; -0.09 to -0.56; -0.12. The Z value varied from previous -2.77 to -3.00 with close p-values. The r^2^ value varied from previous 0.36 to 0.35. The I^2^ did not varied from the previous 83 % and previous 95%CI from ranging from 77; 87 %.

The publication bias reported for elevation with funnel plots with the new corrected data give similar results than previously reported. Similarly, I have performed a new l’Abbé plot for this variable, and the resulting graph is similar. Despite these minor modifications in the values due to a clerical mistake, the overall results, their interpretation, their ranking in Table 1 and the conclusions remain unchanged.

## References

Arnett, E.B. & Hayes, J.P. (2009) Use of conifer snags as roosts by female bats in western Oregon. *Journal of Wildlife Management,* **73,** 214-225.

Fabianek, F., Simard, M.A. & Desrochers, A. (2015) Exploring regional variation in roost selection by bats: evidence from a meta-analysis. *PLoS ONE,* **10,** e0139126.

Fleming HL, Jones JC, Belant JL, Richardson DM. Multi-scale roost site selection by Rafinesque's big-eared bat (Corynorhinus rafinesquii) and southeastern myotis (Myotis austroriparius) in Mississippi. American Midland Naturalist. 2013;**169**(1):43–55.
