# Supplementary material for: Expression of Concern: Exploring Regional Variation in Roost Selection by Bats: Evidence from a Meta-Analysis
Source: PLoS One. 2024 Dec 18;19(12):e0316243. doi: 10.1371/journal.pone.0316243 (PMC11654921; doi:10.1371/journal.pone.0316243)
Supplement: S2 File — These files provide clarifications regarding sources, extraction and conversion of data; and descriptions of errors and their corrections provided by the corresponding author. Readers should also refer to the Expression of Concern notice section on dataset errors. (ZIP) [file pone.0316243.s002.zip › S1-S9 Table Correction Reports/S6_Table_correction_report.docx]

# S6_Table.docx (Distance to water)

I have made a complete review of all references used in the data table, and listed below are the errors I have found and all the points raised regarding this dataset:

- The data used for (Arnett & Hayes 2009) were obtained from his PhD thesis (https://ir.library.oregonstate.edu/concern/graduate_thesis_or_dissertations/ff365816w).
- The data used for (Fabianek *et al.* 2015) were obtained from his PhD thesis (https://library-archives.canada.ca/eng/services/services-libraries/theses/Pages/item.aspx?idNumber=1273433671).
- All the other values reported in the S6_Table that were not mentioned in the points raised above, were obtained from published papers.
- All SE reported in the papers were converted in SD using the following formula SD=SE*sqrt(*n*)
- All distances that were not reported in meters (mostly in km) were converted in meters.
- The number of selected trees reported in Fleming *et al.* (2013) for spring was 33 and not 8. The number of random trees reported in Fleming *et al.* (2013) for spring was 139 and not 157. The number of selected trees reported in Fleming *et al.* (2013) for winter was 19 and not 7. The number of random trees reported in Fleming *et al.* (2013) for winter was 144 and not 147. Thus SD values have been recalculated from SE according to the new sample sizes.
- In Jung *et al.* (2004) there was several types of distances from water structures that were reported, such as small ponds, large ponds, marsh, rivers, etc. I could not find which distances I kept or left away for the previous calculations, nor where I obtained the data for each group of bats (MYSE and MYSP). Because of this, I recalculated all the distances entirely, from the data available in Table 2 for MYSP only (*n* = 26), I extracted mean and SD for each distance to water, from which I generated new random values (according to each sample size given in the paper) by applying a normal distribution with the above mentioned estimates and from these new data, I recalculated an overall mean and SD that I converted in meter. Therefore, for MYSP the estimated mean selected trees (and SD) was 1800 (1940) and not 2283 (871). The estimated mean random trees (and SD) was 1328 (1388) and not 1262 (1388).
- In Boland (2009) the *n* size for the selected trees was 62 and not 60. Therefore, the new SD is 142 and not 139.
- Because of these errors, I rerun the SMD analysis with the corrected values in the S6_Table:

|  | **Selected trees** | | | **Random trees** | | |  |  |
| --- | --- | --- | --- | --- | --- | --- | --- | --- |
| **Study** | ***N*** | **Mean** | **SD** | ***N*** | **Mean** | **SD** | **SMD** | **95 % CI** |
| [[1](#_ENREF_1)] | 105 | 1407 | 1332 | 119 | 1829 | 1385 | -0.30 | -0.57; -0.05 |
| [[1](#_ENREF_1)] | 24 | 1205 | 926 | 23 | 1470 | 1352 | -0.20 | -0.80; 0.35 |
| [[1](#_ENREF_1)] | 42 | 967 | 1056 | 104 | 1883 | 1428 | -0.70 | -1.05; -0.32 |
| [[1](#_ENREF_1)] | 35 | 1582 | 1935 | 33 | 1551 | 1338 | 0.02 | -0.46; 0.49 |
| [[1](#_ENREF_1)] | 22 | 1480 | 1599 | 26 | 1766 | 1127 | -0.20 | -0.78; 0.36 |
| [[2](#_ENREF_2)] | 25 | 627 | 469 | 314 | 580 | 400 | 0.11 | -0.29; 0.52 |
| [[3](#_ENREF_3)] | 40 | 779 | 799 | 40 | 1342 | 663 | -0.80 | -1.21; -0.30 |
| [[4](#_ENREF_4)] | 33 | 197 | 476 | 157 | 256 | 365 | -0.20 | -0.87; 0.55 |
| [[4](#_ENREF_4)] | 19 | 177 | 229 | 147 | 244 | 174 | -0.40 | -1.14; 0.38 |
| [[5](#_ENREF_5)] | 52 | 1032 | 413 | 61 | 770 | 500 | 0.56 | 0.19; 0.94 |
| [[6](#_ENREF_6)] | 26 | 1800 | 1940 | 52 | 1328 | 1388 | 0.30 | -0.26; 0.87 |
| [[6](#_ENREF_6)] | - | - | - | - | - | - | - | - |
| [[7](#_ENREF_7)] | 57 | 127 | 100 | 31 | 121 | 89.1 | 0.06 | -0.38; 0.50 |
| [[8](#_ENREF_8)] | 111 | 333 | 247 | 111 | 429 | 312 | -0.30 | -0.60; -0.07 |
| [[8](#_ENREF_8)] | 57 | 164 | 139 | 57 | 192 | 146 | -0.20 | -0.57; 0.17 |
| [[9](#_ENREF_9)] | 33 | 923 | 925 | 66 | 1217 | 780 | -0.40 | -0.77; 0.07 |
| [[10](#_ENREF_10)] | 17 | 272 | 288 | 21 | 301 | 206 | -0.10 | -0.76; 0.52 |
| [[11](#_ENREF_11)] | 43 | 458 | 315 | 58 | 701 | 350 | -0.70 | -1.13; -0.31 |
| [[11](#_ENREF_11)] | 54 | 759 | 353 | 54 | 855 | 558 | -0.20 | -0.58; 0.17 |
| [[12](#_ENREF_12)] | 23 | 117 | 131 | 46 | 150 | 130 | -0.20 | -0.75; 0.26 |
| [[13](#_ENREF_13)] | 62 | 101 | 142 | 114 | 179 | 139 | -0.56 | -0.87; -0.24 |
| [[13](#_ENREF_13)] | 24 | 219 | 137 | 44 | 182 | 133 | 0.27 | -0.23; 0.77 |
| **Fixed effect** | | |  |  |  |  | **-0.23** | **-0.31; -0.14** |
| **Random effects** | | |  |  |  |  | **-0.20** | **-0.35; -0.06** |
| **Prediction range** | | |  |  |  |  | **-** | **-0.77; 0.37** |

- First, with the merging of both datasets (MYSE and MYSP) reported in Table 2 in Jung *et al.* (2004) the sample size *K* (number of datasets) is now of *K* = 21 instead of 22 in Table 1 (Fabianek, Simard & Desrochers 2015).
- From these new results, I can see that the reported SMD for the random effects model varied from the previously reported -0.16 in Table 1 (Fabianek, Simard & Desrochers 2015) to -0.23 here (see results above). The reported 95%CI also varied from previous -0.33 to 0.00 to -0.35; -0.06. The Z value varied from previous -1.95 to -2.58 with p-values passing from 0.05 to 0.01. The r^2^ value varied from previous 0.10 to 0.07. The I^2^ varied from the previous 68 % with 95%CI (%) ranging from 50 to 79 % to a new 62 % and 95%CI ranging from 39 to 76 %.
- I have recalculated the publication bias reported for this variable with new funnel plots provided, which gave me somewhat similar results than previously reported: t-test for publication bias previously reported was 1.61 with 20 degrees of freedom and a p-value of 0.12. New corresponding values are *t* = 1.2; df = 19; *p* = 0.25. All these values are provided in a new Table 1 provided.
- Similarly, I have performed a new l’Abbé plot for this variable, and the resulting graph is similar (see new results). Despite these modifications in the original values, the overall results, their interpretation, their ranking in Table 1 remain unchanged. However since the p-value of this variable has passed from 0.05 to 0.01 it is now among the five other tree characteristics with significant SMD. The modifications improved the overall results for this variable (which were already nearly significant at 0.05) and this has indeed to be corrected and mentioned properly in the paper, however the main conclusion provided in Fabianek, Simard & Desrochers 2015 (which concerned mainly the tree diameter variable and the following meta-regressions) are clearly not affected be these changes.

##

# References

1. Arnett EB, Hayes JP. Use of conifer snags as roosts by female bats in western Oregon. Journal of Wildlife Management. 2009;73(2):214-25. doi: 10.2193/2007-532.

2. Clement MJ, Castleberry SB. Southeastern myotis (*Myotis austroriparius*) roost selection in cypress-gum swamps. Acta Chiropterologica. 2013;15(1):133-41. doi: 10.3161/150811013x667939.

3. Fabianek F, Simard MA, Racine B. E, Desrochers A. Selection of roosting habitat by male *Myotis* bats in a boreal forest. Canadian Journal of Zoology. 2015;(0):539-46. doi: 10.1139/cjz-2014-0294.

4. Fleming HL, Jones JC, Belant JL, Richardson DM. Multi-scale roost site selection by Rafinesque's big-eared bat (*Corynorhinus rafinesquii*) and southeastern myotis (*Myotis austroriparius*) in Mississippi. American Midland Naturalist. 2013;169(1):43-55. doi: 10.1674/0003-0031-169.1.43.

5. Herder MJ, Jackson JG. Roost preferences of long-legged myotis in northern Arizona. Transactions of the Western Section of the Wildlife Society. 2000;36:1-7.

6. Jung TS, Thompson ID, Titman RD. Roost site selection by forest-dwelling male *Myotis* in central Ontario, Canada. Forest Ecology and Management. 2004;202(1-3):325-35. doi: 10.1016/j.foreco.2004.07.043.

7. Lacki MJ, Schwierjohann JH. Day-roost characteristics of northern bats in mixed mesophytic forest. Journal of Wildlife Management. 2001;65(3):482-8. doi: 10.2307/3803101.

8. Miles AC, Castleberry SB, Miller DA, Conner LM. Multi-scale roost-site selection by evening bats on pine-dominated landscapes in southwest Georgia. Journal of Wildlife Management. 2006;70(5):1191-9. doi: 10.2193/0022-541x(2006)70[1191:mrsbeb]2.0.co;2.

9. Ormsbee PC, McComb WC. Selection of day roosts by female long-legged myotis in the central Oregon Cascade range. Journal of Wildlife Management. 1998;62(2):596-603. doi: 10.2307/3802335.

10. Psyllakis JM, Brigham RM. Characteristics of diurnal roosts used by female *Myotis* bats in sub-boreal forests. Forest Ecology and Management. 2006;223(1-3):93-102. doi: 10.1016/j.foreco.2005.03.071.

11. Rabe MJ, Morrell TE, Green H, Devos JJC, Miller CR. Characteristics of ponderosa pine snag roosts used by reproductive bats in northern Arizona. Journal of Wildlife Management. 1998;62:612-21. doi: 10.2307/3802337.

12. Weller TJ, Zabel CJ. Characteristics of fringed myotis day roosts in northern California. Journal of Wildlife Management. 2001;65(3):489-97. doi: 10.2307/3803102.

13. Boland JL, Hayes JP, Smith WP, Huso MM. Selection of day-roosts by Keen's myotis (*Myotis keenii*) at multiple spatial scales. Journal of Mammalogy. 2009; 90(1):222-34. doi: 10.1644/07-MAMM-A-369.1.
