# Supplementary material for: Expression of Concern: Exploring Regional Variation in Roost Selection by Bats: Evidence from a Meta-Analysis
Source: PLoS One. 2024 Dec 18;19(12):e0316243. doi: 10.1371/journal.pone.0316243 (PMC11654921; doi:10.1371/journal.pone.0316243)
Supplement: S2 File — These files provide clarifications regarding sources, extraction and conversion of data; and descriptions of errors and their corrections provided by the corresponding author. Readers should also refer to the Expression of Concern notice section on dataset errors. (ZIP) [file pone.0316243.s002.zip › S1-S9 Table Correction Reports/S7_Table_correction_report.docx]

# S7_Table.docx (Tree density)

I have made a complete review of all references used in the data table, and listed below are all the errors I have found including all the points raised regarding this dataset:

- The data used for (Broders & Forbes 2004) were obtained from his PhD thesis (reference 62).
- The data used for (Fabianek *et al.* 2015) were obtained from his PhD thesis (https://library-archives.canada.ca/eng/services/services-libraries/theses/Pages/item.aspx?idNumber=1273433671).
- All the other values reported in the S7_Table that were not mentioned in the points raised above, were obtained from published papers.
- All SE reported in the papers were converted in SD using the following formula SD=SE*sqrt(*n*)
- Tree densities reported as the number of stems and stems / 0.1 ha were judged at a comparable scale and as such, were used without conversion (more on this later). All other densities were however converted in stems / 0.01 using cross-products, *i.e*., mean and SE provided for a 0.03 ha plot or a 1 ha plot were converted to 0.1 ha plots so that these reports could be compared on a somewhat similar scale. I am aware this is not perfect, but that was the best approach I could use to maximise the number of studies. Moreover, the fact that the number of stems is not exactly at the same scale of measurement than a surface plot of 0.1 ha is not much of an issue since we are working here with mean differences which weights depend mostly on the size of the SD and the number of samples in each study which are not scale dependant. Moreover, since we relied on random weights based on study-to-study differences, we are taking into account these differences of measurements from studies-to-studies and the resulting weights applied to mean differences are thus smoothed across studies (as one can see in each table when comparing fixed and random weights). So at the end, the mean difference value (which is scale dependant) has only a small impact on the SMD (as long as they remain small) and what really matters at the end is the direction of the effect (i.e., negative or positive), the size of the SD and the sample size used in the study. That is why meta-analyses with random effects are quite robust to these small differences of scale measurements (again, as long as they remain small enough) and that is why I have judged that the benefit of including these studies with different measurements (*i.e*, with the number of stems and stems / 0.1 ha) and having more studies to work with was greater than the prejudice linked to small variations of scale measurement. Finally, I would like to highlight the fact that the goal of these meta-analyses was only to classify tree characteristics based on SMD size and heterogeneity, no matter the source of heterogeneity. The fact that some heterogeneity among studies might result from different scale of measurements was part of the original design and was mentioned clearly in the “limitation and research perspective” section of the discussion: “It is likely that the differences in results among studies were also influenced by measurement methods [64].”
- Due to an error of table header on the previous dataset, all values (N, mean, and SD) given for the selected trees were inversed with those provided for the random trees. Proper corrections have been made and values belonging to each group (selected and random) have been restored to their respective order. Since this tree characteristic is represented by studies with opposed trends (i.e., effects with opposed directions), the SMD of the meta-analysis tends towards zero (with 95%CI crossing the zero value) and thus, the difference from the previous (SMD = 0.06) and the actual SMD (= -0.05) is minimal.
- In Menzel *et al.* (2002), I have used overstory trees instead of understory trees and values were converted from stems / 0.03 ha to stems / 0.1 ha. Therefore, means (SD) for selected and random trees was 74 (40.5) and 93.7 (31.2) and not 83(48.5) and 108(69.3), respectively.
- In Parsons *et al.* (2003), a 100m radius sphere was used to count every tree which represents 12.57 ha that was converted to 0.1 ha by a cross product. Previous values were assumed to be taken from a 100 m x 100m plot which would give 1 ha instead of 12.57 ha. Therefore, means (SD) for selected and random trees was 8(4.2) and 13.1(1.8) and not 100(52,2) and 165(34,2), respectively.
- In Vonhof and Gwilliam (2007) stems were counted under a 0.1 ha plot and did not need to be converted as it was previously. The values reported in Table 1 for each 3 species and at the random stand were reported for deciduous, coniferous and available trees. These values were added to obtain an overall mean and SD values for each species and the random stands. Thus, resulting means (SD) were 41(35), 46(38) and 20(38) for LANO, EPFU and MYCA respectively and 43(37.1) for the random stands, and not 123,4(100,2), 108,9(82,8), 112,5(98,7) and 134(116,5) for the random stands.
- In Boland (2009) the *n* size for the selected trees was 62 and not 60. Therefore, the new SD is 14.2 and not 13.9.

|  | **Selected trees** | | | **Random trees** | | |  |  |
| --- | --- | --- | --- | --- | --- | --- | --- | --- |
| **Study** | ***N*** | **Mean** | **SD** | ***N*** | **Mean** | **SD** | **SMD** | **95 % CI** |
| [[1](#_ENREF_1)] | 164 | 291.7 | 224.1 | 160 | 202 | 161.9 | -0.46 | -0.68; -0.23 |
| [[1](#_ENREF_1)] | 28 | 249.7 | 245.5 | 160 | 202 | 161.9 | -0.27 | -0.67; 0.13 |
| [[2](#_ENREF_2)] | 19 | 450.0 | 158.0 | 38 | 490 | 227.0 | 0.19 | -0.36; 0.74 |
| [[3](#_ENREF_3)] | 55 | 192.3 | 423.5 | 55 | 123 | 61.6 | -0.23 | -0.60; 0.15 |
| [[3](#_ENREF_3)] | 57 | 217.6 | 373.0 | 57 | 205 | 248.4 | -0.04 | -0.41; 0.33 |
| [[3](#_ENREF_3)] | 48 | 322.5 | 805.1 | 48 | 239 | 474.6 | -0.13 | -0.53; 0.27 |
| [[4](#_ENREF_4)] | 25 | 94.0 | 49.3 | 314 | 150 | 140.9 | 0.41 | 0.00; 0.82 |
| [[5](#_ENREF_5)] | 8 | 105.0 | 59.3 | 8 | 150 | 36.3 | 0.87 | -0.17; 1.91 |
| [[5](#_ENREF_5)] | 40 | 147.8 | 68.7 | 40 | 124 | 81.9 | -0.31 | -0.75; 0.13 |
| [[6](#_ENREF_6)] | 15 | 120.0 | 75.1 | 52 | 198 | 53.4 | 1.31 | 0.70; 1.93 |
| [[6](#_ENREF_6)] | 11 | 104.6 | 69.7 | 52 | 198 | 53.4 | 1.64 | 0.92; 2.35 |
| [[7](#_ENREF_7)] | 6 | 590.0 | 279.2 | 50 | 294 | 193.8 | -1.44 | -2.33; -0.55 |
| [[8](#_ENREF_8)] | 12 | 74 | 40.5 | 12 | 93.7 | 31.2 | 0.40 | -0.41; 1.21 |
| [[9](#_ENREF_9)] | 6 | 8 | 4.2 | 4 | 13.1 | 1.8 | 1.27 | -0.18; 2.72 |
| [[10](#_ENREF_10)] | 43 | 111.4 | 73.6 | 58 | 82 | 48.7 | -0.48 | -0.88; -0.08 |
| [[10](#_ENREF_10)] | 54 | 104.7 | 74.2 | 54 | 66.6 | 323.0 | -0.16 | -0.54; 0.22 |
| [[11](#_ENREF_11)] | 46 | 41 | 35 | 112 | 43 | 37.1 | 0.10 | -0.25; 0.44 |
| [[11](#_ENREF_11)] | 46 | 38 | 28.4 | 112 | 43 | 37.1 | 0.23 | -0.11; 0.58 |
| [[11](#_ENREF_11)] | 20 | 38 | 30.9 | 112 | 43 | 37.1 | 0.19 | -0.29; 0.67 |
| [[12](#_ENREF_12)] | 23 | 50.6 | 17.3 | 46 | 57.7 | 26.5 | 0.29 | -0.21; 0.80 |
| [[13](#_ENREF_13)] | 62 | 36.1 | 14.2 | 114 | 38.8 | 15.0 | 0.18 | -0.13; 0.50 |
| [[13](#_ENREF_13)] | 24 | 34.0 | 13.7 | 44 | 32.7 | 14.6 | -0.09 | -0.59; 0.41 |
| [[14](#_ENREF_14)] | 16 | 36.3 | 20.0 | 11 | 28 | 9.0 | -0.49 | -1.27; 0.29 |
| [[14](#_ENREF_14)] | 35 | 27.2 | 5.9 | 57 | 25.2 | 7.6 | -0.28 | -0.71; 0.14 |
| **Fixed effect** | | |  |  |  |  | **0.05** | **-0.04; 0.14** |
| **Random effects** | | |  |  |  |  | **-0.05** | **-0.26; 0.15** |
| **Prediction range** | | |  |  |  |  | - | **-1.00; 0.89** |

- From this new results, and despite all these changes brought in the dataset, I can see that the reported SMD for the random effects model varied from the previously reported 0.06 in Table 1 (Fabianek, Simard & Desrochers 2015) to -0.05 here (see results above). The reported 95%CI also varied from previous -0.15; 0.27 to -0.26; 0.15. The Z value varied from previous 0.55 to -0.50 with p-values passing from 0.58 to 0.61. The r^2^ stayed unchanged. The I^2^ varied from the previous 76 % with 95%CI (%) ranging from 64 to 84 % to a new 75 % and 95%CI ranging from 63 to 83 %.
- I have recalculated the publication bias reported for this variable with new funnel plots provided, which gave me somewhat similar results than previously reported: t-test for publication bias previously reported was -2.13 with 22 degrees of freedom and a p-value of 0.05. New corresponding values are *t* = 2.23; df = 22; *p* = 0.05. All these values are provided in a new Table 1 provided.
- Similarly, I have performed a new l’Abbé plot for this variable, and the resulting graph is similar (see new results). Again, it appears that despite all these modifications in the original values, the overall results, their interpretation, their ranking in Table 1, and the conclusions provided in Fabianek, Simard & Desrochers 2015 remain unchanged.

##

# References

1. Baker MD, Lacki MJ. Day-roosting habitat of female long-legged myotis in ponderosa pine forests. Journal of Wildlife Management. 2006;70(1):207-15. doi: 10.2307/3803562.

2. Brigham RM, Vonhof MJ, Barclay RMR, Gwilliam JC. Roosting behavior and roost-site preferences of forest-dwelling California bats (*Myotis californicus*). Journal of Mammalogy. 1997;78(4):1231-9. doi: 10.2307/1383066.

3. Broders HG, Forbes GJ. Interspecific and intersexual variation in roost-site selection of northern long-eared and little brown bats in the Greater Fundy National Park ecosystem. Journal of Wildlife Management. 2004;68(3):602-10. doi: 10.2193/0022-541x(2004)068[0602:iaivir]2.0.co;2.

4. Clement MJ, Castleberry SB. Southeastern myotis (*Myotis austroriparius*) roost selection in cypress-gum swamps. Acta Chiropterologica. 2013;15(1):133-41. doi: 10.3161/150811013x667939.

5. Fabianek F, Simard MA, Racine B. E, Desrochers A. Selection of roosting habitat by male *Myotis* bats in a boreal forest. Canadian Journal of Zoology. 2015;(0):539-46. doi: 10.1139/cjz-2014-0294.

6. Jung TS, Thompson ID, Titman RD. Roost site selection by forest-dwelling male *Myotis* in central Ontario, Canada. Forest Ecology and Management. 2004;202(1-3):325-35. doi: 10.1016/j.foreco.2004.07.043.

7. Lacki MJ, Baker MD. Day roosts of female fringed myotis (*Myotis thysanodes*) in xeric forests of the Pacific Northwest. Journal of Mammalogy. 2007;88(4):967-73. doi: 10.1644/06-MAMM-A-255R.1.

8. Menzel MA, Owen SF, Ford WM, Edwards JW, Wood PB, Chapman BR, et al. Roost tree selection by northern long-eared bat (*Myotis septentrionalis*) maternity colonies in an industrial forest of the central Appalachian mountains. Forest Ecology and Management. 2002;155(1):107-14. doi: 10.1016/S0378-1127(01)00551-5.

9. Parsons S, Lewis KJ, Psyllakis JM. Relationships between roosting habitat of bats and decay of aspen in the sub-boreal forests of British Columbia. Forest Ecology and Management. 2003;177(1–3):559-70. doi: 10.1016/S0378-1127(02)00448-6.

10. Rabe MJ, Morrell TE, Green H, Devos JJC, Miller CR. Characteristics of ponderosa pine snag roosts used by reproductive bats in northern Arizona. Journal of Wildlife Management. 1998;62:612-21. doi: 10.2307/3802337.

11. Vonhof MJ, Gwilliam JC. Intra- and interspecific patterns of day roost selection by three species of forest-dwelling bats in southern British Columbia. Forest Ecology and Management. 2007;252(1-3):165-75. doi: 10.1016/j.foreco.2007.06.046.

12. Weller TJ, Zabel CJ. Characteristics of fringed myotis day roosts in northern California. Journal of Wildlife Management. 2001;65(3):489-97. doi: 10.2307/3803102.

13. Boland JL, Hayes JP, Smith WP, Huso MM. Selection of day-roosts by Keen's myotis (*Myotis keenii*) at multiple spatial scales. Journal of Mammalogy. 2009; 90(1):222-34. doi: 10.1644/07-MAMM-A-369.1.

14. Lacki MJ, Cox DR, Dodd LE, Dickinson MB. Response of Northern bats (*Myotis septentrionalis*) to prescribed fires in eastern Kentucky forests. Journal of Mammalogy. 2009;90(5):1165-75. doi: 10.1644/08-MAMM-A-349.1.
