# Supplementary material for: Expression of Concern: Exploring Regional Variation in Roost Selection by Bats: Evidence from a Meta-Analysis
Source: PLoS One. 2024 Dec 18;19(12):e0316243. doi: 10.1371/journal.pone.0316243 (PMC11654921; doi:10.1371/journal.pone.0316243)
Supplement: S2 File — These files provide clarifications regarding sources, extraction and conversion of data; and descriptions of errors and their corrections provided by the corresponding author. Readers should also refer to the Expression of Concern notice section on dataset errors. (ZIP) [file pone.0316243.s002.zip › S1-S9 Table Correction Reports/S8_Table_correction_report.docx]

# S8_Table.docx (slope)

I have made a complete review of all references used in the data table, and listed below are all the errors I have found including all the points raised regarding this dataset:

- The data used for (Arnett & Hayes 2009) were obtained from his PhD thesis (https://ir.library.oregonstate.edu/concern/graduate_thesis_or_dissertations/ff365816w).
- The data used for (Broders & Forbes 2004) were obtained from his PhD thesis (reference 62).
- The data used for (Fabianek *et al.* 2015) were obtained from his PhD thesis (https://library-archives.canada.ca/eng/services/services-libraries/theses/Pages/item.aspx?idNumber=1273433671).
- All the other values reported in the S8_Table that were not mentioned in the points raised above, were obtained from published papers.
- All SE reported in the papers were converted in SD using the following formula SD=SE*sqrt(*n*)
- Slopes were mostly reported in percent of slope or degrees in the literature and all slopes reported in degrees were converted in % of slope using the following formula: % slope = (Tan(slope in degree))*100. This was done for most of the studies included in the meta-analysis; however the conversions were forgotten in Fleming *et al.* (2013).
- In Rabe *et al.* (1998) the converted angle value for the mean selected trees was 26.1 and not 29.1 and ne number of selected trees was 53 and not 54.
- After a careful reading of the methodology used by Boland *et al*. (2009), it appears that there was indeed confusion with the meaning of “slope” provided in the paper. In Boland *et al*. (2009), slope-height was used to describe the measurement of the height of the tree including the slope of the terrain and was therefore a tree characteristic and not a stand characteristic for slope terrain. Consequently, the values provided in Boland *et al*. (2009) were removed from the new meta-analyses and the new number of datasets (*K* in Table 1) is now 19 and not 21.

|  | **Selected trees** | | | **Random trees** | | |  |  |
| --- | --- | --- | --- | --- | --- | --- | --- | --- |
| **Study** | ***N*** | **Mean** | **SD** | ***N*** | **Mean** | **SD** | **SMD** | **95 % CI** |
| [[1](#_ENREF_1)] | 105 | 32.4 | 20.5 | 119 | 36.8 | 25.1 | -0.19 | -0.45; 0.07 |
| [[1](#_ENREF_1)] | 24 | 36.4 | 23.5 | 23 | 42.8 | 23.5 | -0.27 | -0.84; 0.31 |
| [[1](#_ENREF_1)] | 42 | 35.6 | 22.7 | 104 | 33.8 | 20.4 | 0.08 | -0.27; 0.44 |
| [[1](#_ENREF_1)] | 35 | 42.3 | 28.4 | 33 | 35.6 | 20.1 | 0.27 | -0.21; 0.75 |
| [[1](#_ENREF_1)] | 22 | 34.1 | 18.8 | 26 | 40.0 | 33.7 | -0.21 | -0.78; 0.36 |
| [[2](#_ENREF_2)] | 164 | 26.7 | 25.6 | 160 | 25.6 | 21.5 | 0.05 | -0.17; 0.26 |
| [[2](#_ENREF_2)] | 28 | 31.2 | 25.9 | 160 | 25.6 | 21.5 | 0.25 | -0.15; 0.65 |
| [[3](#_ENREF_3)] | 55 | 6.6 | 3.7 | 55 | 7.4 | 4.5 | -0.19 | -0.57; 0.18 |
| [[3](#_ENREF_3)] | 57 | 4.6 | 4.5 | 57 | 5.4 | 10.6 | -0.10 | -0.47; 0.27 |
| [[3](#_ENREF_3)] | 48 | 4.6 | 4.2 | 48 | 4.9 | 4.9 | -0.07 | -0.47; 0.33 |
| [[4](#_ENREF_4)] | 33 | 3.3 | 5.0 | 139 | 9.3 | 16.5 | -0.35 | -1.06; 0.37 |
| [[4](#_ENREF_4)] | 19 | 2.6 | 3.0 | 144 | 9.3 | 14.7 | -0.46 | -1.22; 0.30 |
| [[5](#_ENREF_5)] | 52 | 4.0 | 5.1 | 61 | 6.1 | 5.5 | -0.39 | -0.77; -0.02 |
| [[6](#_ENREF_6)] | 16 | 29.1 | 11.7 | 6 | 43.5 | 12.5 | -1.16 | -2.17; -0.15 |
| [[7](#_ENREF_7)] | 6 | 52.3 | 15.3 | 50 | 34.3 | 20.6 | 0.88 | 0.02; 1.74 |
| [[8](#_ENREF_8)] | 12 | 23.3 | 7.9 | 12 | 25.3 | 6.1 | -0.28 | -1.08; 0.53 |
| [[9](#_ENREF_9)] | 43 | 14.1 | 11.5 | 58 | 10.5 | 13.3 | 0.28 | -0.12; 0.68 |
| [[9](#_ENREF_9)] | 53 | 23.1 | 12.9 | 54 | 12.3 | 12.9 | 1.30 | 0.88; 1.71 |
| [[10](#_ENREF_10)] | 23 | 29.7 | 24.9 | 46 | 35.7 | 14.9 | -0.32 | -0.82; 0.19 |
| [[11](#_ENREF_11)] | - | - | - | - | - | - | - | - |
| [[11](#_ENREF_11)] | - | - | - | - | - | - | - | - |
| **Fixed effect** | | |  |  |  |  | **-0.04** | **-0.04; 0.05** |
| **Random effects** | | |  |  |  |  | **-0.05** | **-0.21; 0.11** |
| **Prediction range** | | |  |  |  |  | **-** | **-0.64; 0.55** |

- From this new results, I can see that the reported SMD for the random effects model varied from the previously reported 0.03 in Table 1 (Fabianek, Simard & Desrochers 2015) to -0.05 here (see results above). The reported 95%CI also varied from previous -0.16; 0.21 to -0.21; 0.11. The Z value varied from previous 0.30 to -0.60 with p-values passing from 0.78 to 0.55. The r^2^ varied from previous 0.12 to 0.07. The I^2^ varied from the previous 72 % with 95%CI (%) ranging from 56 to 82 % to a new 64 % and 95%CI ranging from 41 to 78 %.
- I have recalculated the publication bias reported for this variable with new funnel plots provided, which gave me somewhat similar results than previously reported: t-test for publication bias previously reported was -0.50 with 19 degrees of freedom and a p-value of 0.62. New corresponding values are *t* = -0.44; df = 17; *p* = 0.66. All these values are provided in a new Table 1 provided.
- Similarly, I have performed a new l’Abbé plot for this variable, and the resulting graph is similar (see new results). Again, it appears that despite these modifications in the original values, the overall results, their interpretation, their ranking in Table 1, and the conclusions provided in Fabianek, Simard & Desrochers 2015 remain unchanged.

##

# References

1. Arnett EB, Hayes JP. Use of conifer snags as roosts by female bats in western Oregon. Journal of Wildlife Management. 2009;73(2):214-25. doi: 10.2193/2007-532.

2. Baker MD, Lacki MJ. Day-roosting habitat of female long-legged myotis in ponderosa pine forests. Journal of Wildlife Management. 2006;70(1):207-15. doi: 10.2307/3803562.

3. Broders HG, Forbes GJ. Interspecific and intersexual variation in roost-site selection of northern long-eared and little brown bats in the Greater Fundy National Park ecosystem. Journal of Wildlife Management. 2004;68(3):602-10. doi: 10.2193/0022-541x(2004)068[0602:iaivir]2.0.co;2.

4. Fleming HL, Jones JC, Belant JL, Richardson DM. Multi-scale roost site selection by Rafinesque's big-eared bat (*Corynorhinus rafinesquii*) and southeastern myotis (*Myotis austroriparius*) in Mississippi. American Midland Naturalist. 2013;169(1):43-55. doi: 10.1674/0003-0031-169.1.43.

5. Herder MJ, Jackson JG. Roost preferences of long-legged myotis in northern Arizona. Transactions of the Western Section of the Wildlife Society. 2000;36:1-7.

6. Johnson JB, Ford WM, Rodrigue JL, Edwards JW, Johnson CM. Roost selection by male Indiana myotis following forest fires in Central Appalachian hardwood forests. Journal of Fish and Wildlife Management. 2010;1(2):111-21. doi: 10.3996/042010-JFWM-007.

7. Lacki MJ, Baker MD. Day roosts of female fringed myotis (*Myotis thysanodes*) in xeric forests of the Pacific Northwest. Journal of Mammalogy. 2007;88(4):967-73. doi: 10.1644/06-MAMM-A-255R.1.

8. Menzel MA, Owen SF, Ford WM, Edwards JW, Wood PB, Chapman BR, et al. Roost tree selection by northern long-eared bat (*Myotis septentrionalis*) maternity colonies in an industrial forest of the central Appalachian mountains. Forest Ecology and Management. 2002;155(1):107-14. doi: 10.1016/S0378-1127(01)00551-5.

9. Rabe MJ, Morrell TE, Green H, Devos JJC, Miller CR. Characteristics of ponderosa pine snag roosts used by reproductive bats in northern Arizona. Journal of Wildlife Management. 1998;62:612-21. doi: 10.2307/3802337.

10. Weller TJ, Zabel CJ. Characteristics of fringed myotis day roosts in northern California. Journal of Wildlife Management. 2001;65(3):489-97. doi: 10.2307/3803102.

11. Boland JL, Hayes JP, Smith WP, Huso MM. Selection of day-roosts by Keen's myotis (*Myotis keenii*) at multiple spatial scales. Journal of Mammalogy. 2009; 90(1):222-34. doi: 10.1644/07-MAMM-A-369.1.
