# Supplementary material for: Expression of Concern: Exploring Regional Variation in Roost Selection by Bats: Evidence from a Meta-Analysis
Source: PLoS One. 2024 Dec 18;19(12):e0316243. doi: 10.1371/journal.pone.0316243 (PMC11654921; doi:10.1371/journal.pone.0316243)
Supplement: S2 File — These files provide clarifications regarding sources, extraction and conversion of data; and descriptions of errors and their corrections provided by the corresponding author. Readers should also refer to the Expression of Concern notice section on dataset errors. (ZIP) [file pone.0316243.s002.zip › S1-S9 Table Correction Reports/S9_Table_correction_report.docx]

# S9_Table.docx (Remaining bark)

I have made a complete review of all references used in the data table, and listed below are all the errors I have found including all the points raised regarding this dataset:

- The data used for (Broders & Forbes 2004) were obtained from his PhD thesis (reference 62).
- The data used for (Carter 2003) were obtained from his PhD thesis (reference 63).
- The data used for (Fabianek *et al.* 2015) were obtained from his PhD thesis (https://library-archives.canada.ca/eng/services/services-libraries/theses/Pages/item.aspx?idNumber=1273433671).
- All the other values reported in the S8_Table that were not mentioned in the points raised above, were obtained from published papers.
- All SE reported in the papers were converted in SD using the following formula SD=SE*sqrt(*n*).
- In Backer and Lacki (2006) the values of remaining bark were reported in cm^2^. Since the total size of the trees was not reported, I had to convert the remaining bark in percent using a mean estimation of the total of area (in cm^2^) of each group of snags (large-flyout, small-flyout, and random). To do so, and since trees have a coning shape, I have first used the Pythagorean equation ((snag mean DBH /200)^2^ + (snag mean height)^2^ = sqrt(snag mean hypotenuse in meter)) to calculate the hypotenuse of each group of snags. From the hypotenuse (in m), I could estimate the mean area of each group of snags, by using the following formula: snag area (cm^2^) = (pi*(snag mean DBH /200)*mean snag hypotenuse)*100. Then from the estimated mean area of snags in cm^2^ (*i.e.*, 4072 cm^2^ for large-flyout, 2554 cm^2^ for small-flyout, and 1247 cm^2^ for random), I could calculate the percentage of remaining bark using the snag bark cover values (cm^2^) provided in table 3: (mean snag bark cover / mean area of snags)*100 = remaining bark in %. The previous mean remaining bark in % for the small-flyout group was correctly calculated, but the wrong mean area of snags were inadvertently used for the two other groups of snags (i.e., large-flyout, and random). Thus, the new estimated means (and SD) for each group of snags (large-flyout, small-flyout, and random) should be 64.6 (34.9) for small-flyout, 40.5 (21.9) for large-flyout and 47.4 (54.6) for random trees, and not 64.6 (44.5) for small-flyout, 82.5 (47.1) for large-flyout and 29.6 (34.0) for random trees.
- In Lacki *et al.* (2010), I was not able to retrieve the original values provided in the dataset. I am not sure if these values were coming from another paper or not. Therefore, I have decided to remove these values from the dataset and consequently the new number of datasets (*K* in Table 1) is now 25 and not 26.
- In Boland (2009) the *n* size for the selected trees was 62 and not 60. Therefore, the new SD is 29.1 and not 28.7.

|  | **Selected trees** | | | **Random trees** | | |  |  |
| --- | --- | --- | --- | --- | --- | --- | --- | --- |
| **Study** | ***N*** | **Mean** | **SD** | ***N*** | **Mean** | **SD** | **SMD** | **95 % CI** |
| [[1](#_ENREF_1)] | 164 | 64.6 | 34.9 | 160 | 47.4 | 54.6 | 0.38 | 0.16; 0.60 |
| [[1](#_ENREF_1)] | 28 | 40.5 | 21.9 | 160 | 47.4 | 54.6 | -0.13 | -0.54; 0.27 |
| [[2](#_ENREF_2)] | 19 | 75.0 | 29.6 | 38 | 86.0 | 27.3 | -0.39 | -0.94; 0.17 |
| [[3](#_ENREF_3)] | 55 | 78.5 | 30.4 | 55 | 73.7 | 33.4 | 0.15 | -0.22; 0.52 |
| [[3](#_ENREF_3)] | 57 | 74.4 | 31.7 | 57 | 59.0 | 37.8 | 0.44 | 0.07; 0.81 |
| [[3](#_ENREF_3)] | 48 | 72.1 | 31.2 | 48 | 69.0 | 34.6 | 0.09 | -0.31; 0.49 |
| [[4](#_ENREF_4)] | 11 | 81.1 | 16.9 | 14 | 76.2 | 16.5 | 0.28 | -0.51; 1.08 |
| [[4](#_ENREF_4)] | 20 | 69.1 | 18.3 | 5 | 69.8 | 23.0 | -0.04 | -1.02; 0.94 |
| [[5](#_ENREF_5)] | 47 | 47.0 | 26.7 | 37 | 55.0 | 35.3 | -0.26 | -0.69; 0.18 |
| [[5](#_ENREF_5)] | 19 | 79.2 | 30.9 | 46 | 63.8 | 38.4 | 0.42 | -0.12; 0.96 |
| [[6](#_ENREF_6)] | 8 | 93.1 | 15.8 | 8 | 70.6 | 34.9 | 0.79 | -0.24; 1.81 |
| [[6](#_ENREF_6)] | 40 | 0.7 | 26.7 | 40 | 54.5 | 39.7 | -1.57 | -2.08; -1.07 |
| [[7](#_ENREF_7)] | 34 | 76.8 | 31.0 | 15 | 46.7 | 31.8 | 0.95 | 0.31; 1.59 |
| [[8](#_ENREF_8)] | 15 | 61.0 | 19.8 | 52 | 77.0 | 32.5 | -0.52 | -1.11; 0.06 |
| [[8](#_ENREF_8)] | 11 | 63.6 | 27.2 | 52 | 77.0 | 32.5 | -0.42 | -1.07; 0.24 |
| [[9](#_ENREF_9)] | 56 | 68.8 | 33.1 | 1438 | 98.7 | 9.9 | -2.58 | -2.86; -2.30 |
| [[10](#_ENREF_10)] | - | - | - | - | - | - | - | - |
| [[11](#_ENREF_11)] | 19 | 78.1 | 17.5 | 25 | 85.8 | 16.0 | -0.45 | -1.06; 0.15 |
| [[12](#_ENREF_12)] | 47 | 78.0 | 38.4 | 47 | 66.0 | 13.0 | 0.42 | 0.01; 0.82 |
| [[13](#_ENREF_13)] | 46 | 93.0 | 17.6 | 112 | 72.0 | 26.2 | 0.87 | 0.51; 1.23 |
| [[13](#_ENREF_13)] | 46 | 90.0 | 23.3 | 112 | 72.0 | 26.2 | 0.71 | 0.35; 1.06 |
| [[13](#_ENREF_13)] | 20 | 70.0 | 28.0 | 112 | 72.0 | 26.2 | -0.08 | -0.55; 0.40 |
| [[14](#_ENREF_14)] | 62 | 63.0 | 29.1 | 114 | 90.6 | 31.0 | -0.91 | -1.23; -0.58 |
| [[14](#_ENREF_14)] | 24 | 37.4 | 29.4 | 44 | 78.2 | 29.2 | -1.38 | -1.93; -0.83 |
| [[15](#_ENREF_15)] | 16 | 62.0 | 36.0 | 11 | 40.0 | 33.8 | 0.61 | -0.18; 1.39 |
| [[15](#_ENREF_15)] | 35 | 83.0 | 26.0 | 57 | 46.0 | 37.0 | 1.10 | 0.65; 1.55 |
| **Fixed effect** | | |  |  |  |  | **-0.15** | **-0.24; -0.06** |
| **Random effects** | | |  |  |  |  | **-0.07** | **-0.41; 0.27** |
| **Prediction range** | | |  |  |  |  | - | **-1.82; 1.67** |

- From these new results, I can see that the reported SMD for the random effects model varied from the previously reported 0.05 in Table 1 (Fabianek, Simard & Desrochers 2015) to -0.07 here (see results above). The reported 95%CI also varied from previous -0.31; 0.41 to -0.41; 0.27. The Z value varied from previous 0.28 to -0.43 with p-values passing from 0.78 to 0.67. The r^2^ changed from 0.80 to 0.68 and the I^2^ [with corresponding 95%CI] changed from 96 % [95; 97] to 95 % [94; 96].
- I have recalculated the publication bias reported for this variable with new funnel plots provided, which gave me somewhat similar results than previously reported: t-test for publication bias previously reported was -0.17 with 24 degrees of freedom and a p-value of 0.86. New corresponding values are *t* = 0.51; df = 23; *p* = 0.61. All these values are provided in a new Table 1 provided.
- Similarly, I have performed a new l’Abbé plot for this variable, and the resulting graph is similar (see new results). Again, it appears that despite these modifications in the original values, the overall results, their interpretation, their ranking in Table 1, and the conclusions provided in Fabianek, Simard & Desrochers 2015 remain unchanged.

##

# References

1. Baker MD, Lacki MJ. Day-roosting habitat of female long-legged myotis in ponderosa pine forests. Journal of Wildlife Management. 2006;70(1):207-15. doi: 10.2307/3803562.

2. Brigham RM, Vonhof MJ, Barclay RMR, Gwilliam JC. Roosting behavior and roost-site preferences of forest-dwelling California bats (*Myotis californicus*). Journal of Mammalogy. 1997;78(4):1231-9. doi: 10.2307/1383066.

3. Broders HG, Forbes GJ. Interspecific and intersexual variation in roost-site selection of northern long-eared and little brown bats in the Greater Fundy National Park ecosystem. Journal of Wildlife Management. 2004;68(3):602-10. doi: 10.2193/0022-541x(2004)068[0602:iaivir]2.0.co;2.

4. Callahan EV, Drobney RD, Clawson RL. Selection of summer roosting sites by Indiana bats (*Myotis sodalis*) in Missouri. Journal of Mammalogy. 1997;78(3):818-25. doi: 10.2307/1382939.

5. Carter TC. Summer habitat use of roost trees by the endangered Indiana bat *(Myotis sodalis*) in the Shawnee National Forest of southern Illinois. Southern Illinois: Carbondale University; 2003.

6. Fabianek F, Simard MA, Racine B. E, Desrochers A. Selection of roosting habitat by male *Myotis* bats in a boreal forest. Canadian Journal of Zoology. 2015;(0):539-46. doi: 10.1139/cjz-2014-0294.

7. Johnson JB, Ford WM, Rodrigue JL, Edwards JW, Johnson CM. Roost selection by male Indiana myotis following forest fires in Central Appalachian hardwood forests. Journal of Fish and Wildlife Management. 2010;1(2):111-21. doi: 10.3996/042010-JFWM-007.

8. Jung TS, Thompson ID, Titman RD. Roost site selection by forest-dwelling male *Myotis* in central Ontario, Canada. Forest Ecology and Management. 2004;202(1-3):325-35. doi: 10.1016/j.foreco.2004.07.043.

9. Kniowski AB, Gehrt SD. Summer ecology of Indiana bats in Ohio. Columbus, OH, USA: 2011.

10. Lacki MJ, Baker MD, Johnson JS. Geographic variation in roost-site selection of long-legged myotis in the Pacific Northwest. Journal of Wildlife Management. 2010;74(6):1218-28. doi: 10.2307/40801116.

11. Psyllakis JM, Brigham RM. Characteristics of diurnal roosts used by female *Myotis* bats in sub-boreal forests. Forest Ecology and Management. 2006;223(1-3):93-102. doi: 10.1016/j.foreco.2005.03.071.

12. Sasse DB, Pekins PJ. Summer roosting ecology of northern long-eared bats (*Myotis septentrionalis*) in the White Mountain National Forest. In: Barclay RMR, Brigham RM, editors. Bats and Forests Symposium; October 19-21, 1995; Organized by the British Columbia Ministry of Forests. Victoria, BC1996. p. 91-101.

13. Vonhof MJ, Gwilliam JC. Intra- and interspecific patterns of day roost selection by three species of forest-dwelling bats in southern British Columbia. Forest Ecology and Management. 2007;252(1-3):165-75. doi: 10.1016/j.foreco.2007.06.046.

14. Boland JL, Hayes JP, Smith WP, Huso MM. Selection of day-roosts by Keen's myotis (*Myotis keenii*) at multiple spatial scales. Journal of Mammalogy. 2009; 90(1):222-34. doi: 10.1644/07-MAMM-A-369.1.

15. Lacki MJ, Cox DR, Dodd LE, Dickinson MB. Response of Northern bats (*Myotis septentrionalis*) to prescribed fires in eastern Kentucky forests. Journal of Mammalogy. 2009;90(5):1165-75. doi: 10.1644/08-MAMM-A-349.1.
